# Synthetic Strategy for mRNA Encapsulation and Gene Delivery with Nanoscale Metal-Organic Frameworks

**DOI:** 10.1002/adfm.202504465

**Published:** 2025-05-19

**Authors:** Harrison Douglas Lawson, Huy Hoang Nguyen, Keng-Jung Lee, Nattarat Wongsuwan, Ayesha Tupe, Mengrou Lu, Mariah Lynn Arral, Anne Behre, Zihan Ling, Kathryn Ann Whitehead, Adam Walter Feinberg, Xi Ren, Si-Yang Zheng

**Affiliations:** Biomedical Engineering Department, Carnegie Mellon University, 5000 Forbes Avenue, Pittsburgh, PA 15213, USA; Chemical Engineering Department, Carnegie Mellon University, 5000 Forbes Avenue, Pittsburgh, PA 15213, USA; Biomedical Engineering Department, Carnegie Mellon University, 5000 Forbes Avenue, Pittsburgh, PA 15213, USA; Biomedical Engineering Department, Carnegie Mellon University, 5000 Forbes Avenue, Pittsburgh, PA 15213, USA; Biomedical Engineering Department, Carnegie Mellon University, 5000 Forbes Avenue, Pittsburgh, PA 15213, USA; Biomedical Engineering Department, Carnegie Mellon University, 5000 Forbes Avenue, Pittsburgh, PA 15213, USA; Chemical Engineering Department, Carnegie Mellon University, 5000 Forbes Avenue, Pittsburgh, PA 15213, USA; Biomedical Engineering Department, Carnegie Mellon University, 5000 Forbes Avenue, Pittsburgh, PA 15213, USA; Chemical Engineering Department, Carnegie Mellon University, 5000 Forbes Avenue, Pittsburgh, PA 15213, USA; Biomedical Engineering Department, Carnegie Mellon University, 5000 Forbes Avenue, Pittsburgh, PA 15213, USA; Biomedical Engineering Department, Carnegie Mellon University, 5000 Forbes Avenue, Pittsburgh, PA 15213, USA; Biomedical Engineering Department, Carnegie Mellon University, 5000 Forbes Avenue, Pittsburgh, PA 15213, USA; Chemical Engineering Department, Carnegie Mellon University, 5000 Forbes Avenue, Pittsburgh, PA 15213, USA; Biomedical Engineering Department, Carnegie Mellon University, 5000 Forbes Avenue, Pittsburgh, PA 15213, USA; Material Science and Engineering Department, Carnegie Mellon University, 5000 Forbes Avenue, Pittsburgh, PA 15213, USA; Biomedical Engineering Department, Carnegie Mellon University, 5000 Forbes Avenue, Pittsburgh, PA 15213, USA; Biomedical Engineering Department, Carnegie Mellon University, 5000 Forbes Avenue, Pittsburgh, PA 15213, USA; Electrical and Computer Engineering Department, Carnegie Mellon University, 5000 Forbes Avenue, Pittsburgh, PA 15213, USA

**Keywords:** drug delivery, in vivo, metal-organic frameworks, mRNA, nucleic acid, stability

## Abstract

Metal-organic frameworks (MOFs) have evolved from uses in catalysis and gas storage to exciting applications in biomedicine, particularly in drug delivery. Initially, MOFs are primarily used to deliver small molecules, recent innovations have shifted focus toward more complex nucleic acids like DNA, short guide RNA (sgRNA), and short interfering RNA (siRNA). Remarkably, no studies to date have demonstrated the encapsulation and delivery of messenger RNA (mRNA) via MOFs in vitro and in vivo. This study addresses that gap by identifying synthetic conditions to encapsulate and deliver mRNA using zeolitic imidazole framework-8 (ZIF-8). Early attempts show mRNA loading in ZIF-8 but loss of mRNA in biological media. To overcome this challenge, polyethyleneimine (PEI) is incorporated into the formulation, forming a robust polymer complex core-MOF shell particle. This system stabilizes mRNA complexes and delays their release, resulting in effective protein expression in multiple cell lines and mice, performing on par with commercial lipid-based systems. Here, the first investigation into thermally stable mRNA storage using ZIF-8 demonstrates successful protein expression after three months of room-temperature storage in vitro and one month in vivo. These findings broaden the scope of MOF-based therapeutic delivery and open new avenues for long-term mRNA storage and transport.

## 1. Introduction

Since 2020, the concept of nucleic acid delivery has permeated households, thanks to the introduction of mRNA vaccines during the COVID-19 pandemic. Nucleic acid therapies, including vaccines, employ vectors to transport nucleic acid cargo, influencing gene expression or function. Naked DNAs and RNAs, as therapeutic agents, are charged and too large and fragile to traverse cellular membranes. They are also susceptible to degradation by nucleases in tissue and body fluids.^[1,2]^ Consequently, the design of gene therapy vectors plays a pivotal role in ensuring the safety and efficacy of the treatment.^[3–7]^

Viral vectors have proven effective for nucleic acid. However, the clinical translation of viral vectors has been impeded by adverse immune responses, high production costs, off-target insertions, limited cargo gene size, and potential pathogenicity.^[4,8]^ Given these limitations of viral vectors, attention has pivoted towards non-viral vectors for nucleic acid delivery. Non-viral carriers are easier to produce, have better biocompatibility, and provide larger gene-carrying capacity than viral vectors.^[5,6]^

Further, non-viral vectors can be designed with a wide variety of chemistries, and this tunability enables unique functionalities at varied stages of the development and delivery process. For example, these vectors have taken the form of both organic vectors (i.e., liposome and polymer particles) and inorganic vectors (i.e., mesoporous silica, gold, carbon nanotubes, etc.).^[7]^ This variety of vectors provides access to stimuli-responsive release mechanisms (i.e., photo- and thermal-irradiation) and co-delivery of lysosomotropic reagents which improve nucleic acid release into the cytosol.^[7]^ Although non-viral vectors mark progress and improvements in design and desired properties for nucleic acid delivery vehicles, challenges remain.

Cumbersome syntheses, low loading capacities, and nucleic acid stability still limit the applicability of current non-viral vectors.^[3,4,9,10]^ Polymer systems and some lipid systems use techniques that can destabilize nucleic acids or struggle with phase separation of the nucleic acid.^[4,9]^ Additionally, residual catalysts and impurities from syntheses may contaminate vectors and degrade the nucleic acid therapeutic.^[11]^ Lipid-based carriers are common for nucleic acid delivery. Unfortunately, lipid nanoparticles (LNP) have reportedly low loading capacity, ≈2–3 RNA strands per LNP.^[10]^ Of significant concern is the lack of efficient gene expression from current non-viral vectors caused by nucleic acid degradation due to physiological and thermal storage conditions^[6]^ The limitations of current nucleic acid therapy carriers warrant ongoing efforts to design improved formulations. MOFs emerge as a promising solution to address challenges faced by current non-viral vectors, offering gentle loading methods,^[8,12–14]^ high loading capacities,^[8,15,16]^ and the potential to protect and stabilize nucleic acids chemically and physically.^[14]^

MOFs are porous coordinated polymers formed from metal ions such as zirconium (IV), iron (III), and zinc (II) and organic ligands that feature multiple amine and carboxyl groups.^[17]^ The self-assembled architecture of MOFs is highly porous and tunable, which has led to wide applications of MOFs in fields such as catalysis, gas absorption and separation, sensors, energy storage, water treatment, and drug delivery.^[17]^ Specific to drug delivery, MOFs have high drug loading capacity, biodegradability, biocompatibility, and a unique ability to deliver a variety of therapeutic modalities, including small molecules,^[18–21]^ gaseous transmitter molecules,^[22]^ biomolecules,^[8,14,23–26]^ and whole cell therapies.^[13,27]^ MOF-based drug loading can be achieved through surface attachment, post-synthetic pore encapsulation, one-pot coprecipitation, and biomimetic mineralization, reviewed in more detail by Lawson et al.^[17]^ Interestingly, Biomimetic mineralization has been demonstrated with proteins and nucleic acids, stabilizing proteins chemically and physically.^[14,17]^

To date, only a few MOF-based delivery platforms have been designed for nucleic acid delivery, briefly reviewed in Table 1. Early works focused on the surface loading of short interfering RNA (siRNA) onto UiO-66^[28]^ and MIL-101(Fe)^[29]^ for multimodal therapies combating multidrug-resistant cancers. A later study completed by Hidalgo et al. studied the dependency of post-synthetic, siRNA pore encapsulation on MOF physiochemical properties through pH adjustments and ligand functionalization.^[30]^ Along with the successful encapsulation of siRNA, this study demonstrated the delivery of microRNA-145 (miRNA-145) and gene knockdown in colorectal cancer cells.^[30]^ Also using post-synthetic pore encapsulation, Peng et al. demonstrated the stabilization of single-stranded DNA (ssDNA, 11–53 nucleotides) in Ni-IRMOF-74, emphasizing the tailorable nature of MOFs with isoreticular chemistry. Interestingly, Ni-IRMOF-74 exhibited ssDNA delivery that rivaled the performance of current commercial transfection reagents in immune cells.^[31]^ Altogether, these studies show the success of MOFs in delivering short nucleic acid therapies via surface loading and pore encapsulation. While these encapsulation strategies work for small nucleic acids, it is acknowledged that alternative methods are better suited for larger nucleic acids such as plasmid DNA (pDNA) and mRNA. This is due to factors like their stability, physical size, and structure, which can hinder their incorporation using the above-mentioned encapsulation approaches.^[16,32]^

Biomimetic mineralization and one-pot coprecipitation strategies have demonstrated successful encapsulation and delivery of large nucleic acids. These methods create a self-assembled cage around their nucleic acid cargo, engulfing large nucleic acids and protecting them from physiological conditions.^[8,14]^ Poddar et al. demonstrated the biomimetic mineralization of ZIF-8 and delivery of a pDNA (≈6.5 kilo-base pairs(kbp)) expressing a proof-of-concept enhanced Green Fluorescent Protein (eGFP) in pancreatic islet cells and human epithelial prostate cancer cells.^[8,32]^ Pushing their work further, a 9.2-kbp plasmid therapeutic was delivered as prostate cancer therapy.^[33]^ Coincidingly, Li et al. described the one-pot coprecipitation of pDNA with nanoscale ZIF-8 and a polycationic polymer, polyethyleneimine. Improvements in loading efficiency were demonstrated over biomimetic mineralization with comparable transfection efficiency to commercial lipid-based reagents.^[16]^ Interestingly, one group did report successful surface loading and delivery of a 10-kbp plasmid; however, their loading capacity was two to three-fold lower.

Unsurprisingly, little literature exists on the delivery of mRNA with MOFs, possibly due to the fragility of mRNA compared to its shorter counterparts (siRNA and miRNA).^[34]^ Furthermore, no studies have delivered mRNA using MOFs in vivo. mRNA is extremely fragile, readily susceptible to intramolecular catalysis of strand breakage, oxidation, or reactions with impurities such as aldehydes, metals, and peroxides, and degradation by ubiquitous RNases, rendering the mRNA inactive.^[11]^ To our knowledge, the coprecipitation and delivery of mRNA by MOFs remain underexplored, and our work seeks to fill this gap.

## 2. Overview of the Study Design

This study describes the encapsulation synthesis development, integration, and delivery of mRNA with ZIF-8, which is a MOF that is widely utilized in biomedical applications due to its low in vitro and in vivo toxicity.^[17]^ An innovative one-pot coprecipitation method is employed for this purpose. Given the inherent fragility of mRNA, this investigation commences by delving into synthetic strategies and handling techniques that preserve the mRNA’s expressibility. Specifically, the stability of the mRNA and its expression in vitro is systematically evaluated, considering the impact of ZIF-8 synthesis conditions, ZIF-8 incubation, and encapsulation within ZIF-8.

**Scheme 1.**
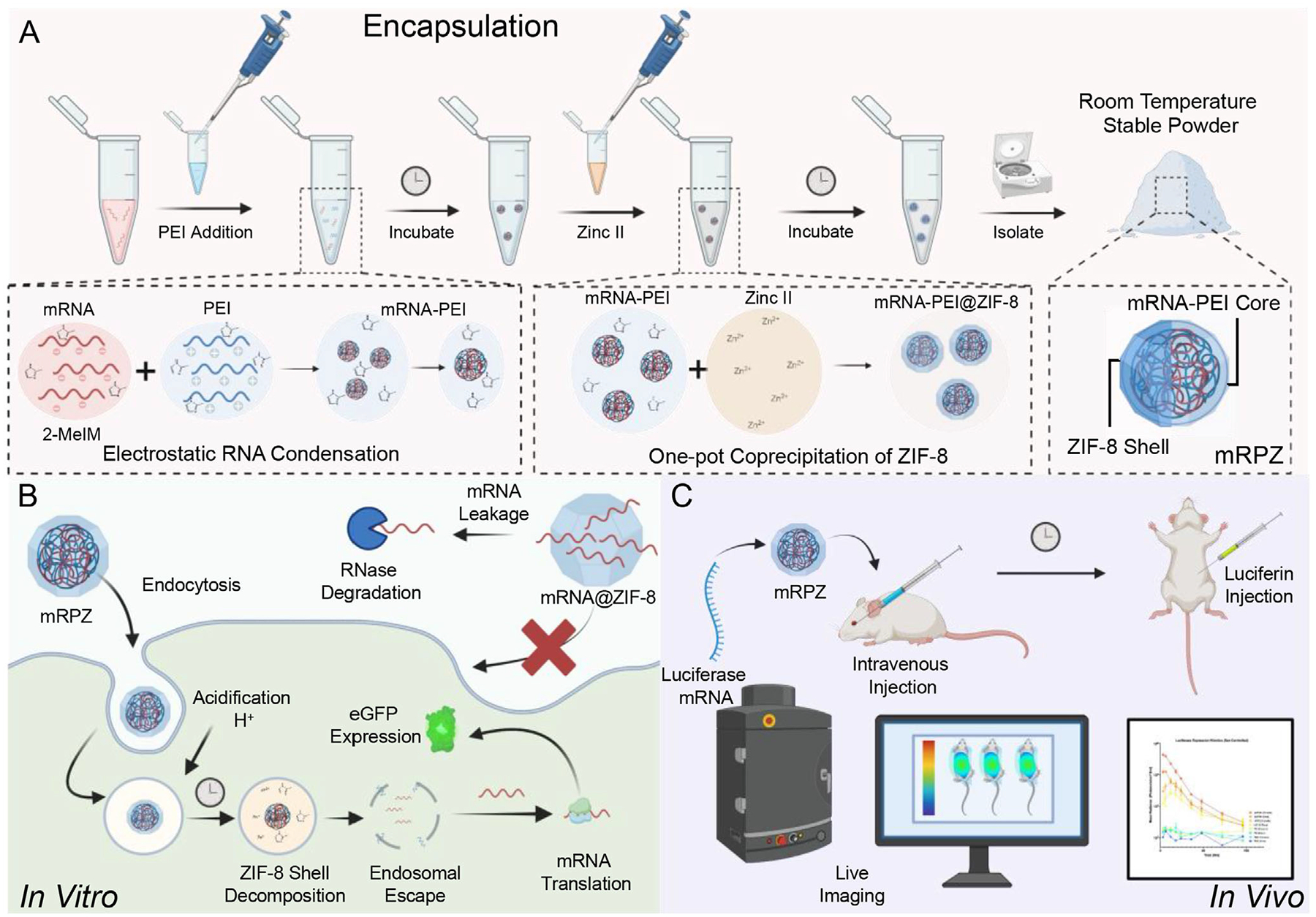
Schematic illustration of electrostatic condensation of mRNA with PEI and one-pot coprecipitation of mRNA complexes inside ZIF-8 (mRPZ), creating a polymer core-MOF shell particle **(A)**. mRNA@ZIF-8 is unable to deliver mRNA due to leakage, while delivery with mRPZ retains mRNA and leads to the successful, delayed expression of eGFP in vitro **(B)** and firefly luciferase in vivo **(C)**.

Recognizing the “leaky” nature of ZIF-8,^[39,40]^ this approach turned towards a ZIF-8-based polymer composite mRNA delivery system, which involves initially complexing mRNA with PEI in a 2-methyl imidazole (2-MeIM) precursor solution, followed by zinc addition and encapsulation within ZIF-8 to form mRNA-PEI@ZIF-8 (mRPZ) nanoparticles (Scheme 1A). Extensive characterization of the mRPZ nanoparticles confirms the encapsulation of complexed mRNA within ZIF-8. This robust polymer complex core-MOF shell particle effectively retains and delivers mRNA, producing eGFP expression in vitro (Scheme 1B) and firefly luciferase expression in vivo (Scheme 1C). Comparative analyses were performed against commercial transfection reagents and mRNA-PEI alone. Remarkably, our findings demonstrate improved performance over mRNA-PEI, delayed expression kinetics, and the stabilization of mRNA complexes within mRPZ nanoparticles, with viable transfection maintained even after 3 months of room-temperature storage in vitro and 1 month in vivo.

This research expands upon MOF-based nucleic acid loading methods and furthers our understanding of MOF-mRNA compatibility. It extends the applications of MOFs in drug delivery, showcasing the potential of ZIF-8 as a versatile platform for enhancing mRNA delivery system stability and transfection efficacy in vivo and in vivo.

## 3. Results and Discussion

### 3.1. Synthesis Development

ZIF-8 emerges as a promising choice for mRNA delivery, given its extensive exploration in biomedical and drug delivery studies. The unique ability of ZIF-8 to mineralize under gentle aqueous conditions, a process known as biomimetic mineralization which has been utilized to encapsulate biomolecules and cells,^[8,14,32]^ and degrade at acidic pH makes it an ideal candidate for mRNA delivery, compared to other MOFs.^[8,15,32]^ Recognizing the delicate nature of mRNA, our investigation focused on identifying synthetic conditions for ZIF-8 encapsulation that preserve mRNA stability, considering mRNA length and expressibility in vitro. Initially, we assessed mRNA length using a native agarose gel, and expressibility was evaluated with treated mRNA and Lipofectamine Messenger Max (Lipo MM) in HEK293t cells.

**Figure 1.**
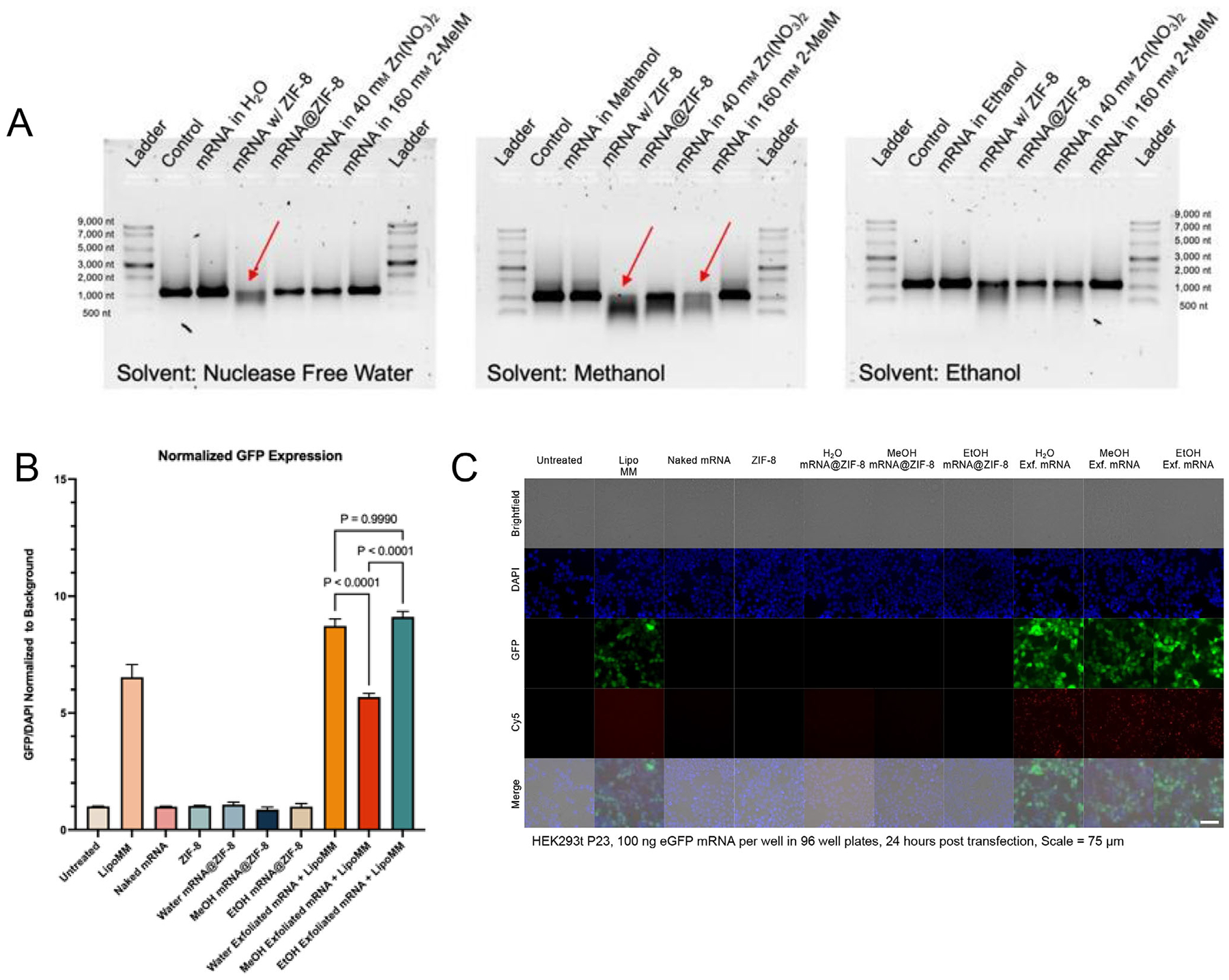
The stability of the mRNA exposed to different experimental conditions was assessed using A) native RNA gel electrophoresis. Clear mRNA degradation (reduction in length) is noted with red arrows. The expressibility of the encapsulated Cy5-eGFP-mRNA was assessed by transfection in HEK293t cells. The resulting eGFP expression was quantified with a B) plate reader and normalized to the background. C) Fluorescence microscopy was used to visualize cell nuclei (DAPI), cell mRNA uptake (Cy5), and mRNA expression (eGFP). Scale bar is 75 μm. Data presented as mean ± standard error of the mean (StdEM), sample sizes N = 16, and statistical significance (P) was assessed with one-way ANOVA.

For encapsulation, biomimetic concentrations of 160 mM 2-MeIM and 40 mM Zn (NO_3_)_2_·6H_2_O were employed, along with common solvents for ZIF-8 synthesis, namely nuclease-free water, ethanol, and methanol. Note that the biomimetic mineralization of ZIF-8 has not been demonstrated to occur in ethanol or methanol; hence, these synthetic methods are considered coprecipitation here. Individual samples of mRNA underwent the following experimental conditions: a 2-hour incubation in synthesis conditions for encapsulation, a 2-hour co-incubation of mRNA with synthesized ZIF-8 previously reported in the literature,^[41]^ or an encapsulation within ZIF-8 (mRNA@ZIF-8) using nuclease-free water, ethanol, and methanol. ZIF-8 and mRNA@ZIF-8 generated were characterized using powder x-ray diffraction (PXRD) and scanning electron microscopy (SEM), as shown in Figure S1 (Supporting Information). After isolating or exfoliating mRNA from ZIF-8 using EDTA (exfoliation described in more detail in Figure S2, Supporting Information), the samples were analyzed on a native RNA gel. From Figure 1A, the native RNA gel reveals evident mRNA degradation, indicated by red arrows, when incubated with ZIF-8 in methanol and subjected to 40 mM Zn (NO_3_)_2_·6H_2_O in methanol. Similarly, mRNA incubation with ZIF-8 in nuclease-free water exhibits visible degradation, whereas incubation with precursor solutions and encapsulated mRNA shows no degradation. The water-synthesized samples lacked matching PXRD patterns to simulated ZIF-8 and featured a variety of morphologies Figure S1 (Supporting Information). Ethanol demonstrates minimal to no mRNA degradation under all tested conditions, and mRNA@ZIF-8 generated in ethanol produced PXRD patterns agreeing with simulated ZIF-8 and monodispersed particles.

After evaluating mRNA length, we conducted in vitro transfections to assess mRNA expressibility. Briefly, Cy5-eGFP-mRNA samples encapsulated within ZIF-8 were prepared with nuclease-free water, methanol, and ethanol and isolated with centrifugation. The particles were then introduced to HEK293t cells. As controls, mRNA samples exfoliated from mRNA@ZIF-8 (Exf. mRNA@ZIF-8), prepared with all mentioned solvents, were applied to transfect the same cells using Lipo MM. Following a 24-h incubation, cells were fixed, and fluorescence was observed under a microscope, with quantification performed using a plate reader (Figure 1B,C). The presence of Cy5 (red) indicated mRNA uptake, while eGFP (green) indicated expressed mRNA. Interestingly, mRNA@ZIF-8 synthesized with any of the mentioned solvents did not induce eGFP expression, and no Cy5 signal was visible. The lack of Cy5 signal suggests a delivery issue with mRNA@ZIF-8 as exfoliated mRNA delivered with Lipo MM exhibited eGFP expression. Additionally, eGFP expression levels were compared among exfoliated mRNA samples. While particles synthesized with water and ethanol showed statistically similar eGFP expression, samples synthesized with methanol exhibited significantly lower eGFP expression, consistent with our earlier findings that mRNA is incompatible with the synthetic conditions in methanol.

mRNA@ZIF-8 synthesized with any solvent maintained mRNA length and expressibility, but significant mRNA degradation occurred with methanol-based synthesis conditions and when mRNA was incubated with ZIF-8 in water or methanol. Interestingly, ZIF-8-mRNA co-incubation causes degradation in all solvents tested except PBS and EtOH for up to 4 h (Figure S3, Supporting Information). Moreover, the deviation of mRNA@ZIF-8 particles synthesized in water from simulated ZIF-8 PXRD patterns and morphology indicated that these biomimetic mineralization concentrations were insufficient for uniform ZIF-8 nanoparticle formation. Based on these findings, ethanol-based coprecipitation was identified as the optimal solvent for mRNA encapsulation in ZIF-8. Ethanol-based coprecipitation maintained mRNA length better than tested solvents when dried and stored for up to 7 days (Figure S4, Supporting Information) and was stable in PBS and EtOH post-synthesis (Figure S5, Supporting Information). Having identified a compatible synthetic strategy for one-pot coprecipitation of mRNA@ZIF-8, we spearheaded the delivery issue of mRNA@ZIF-8.

**Figure 2.**
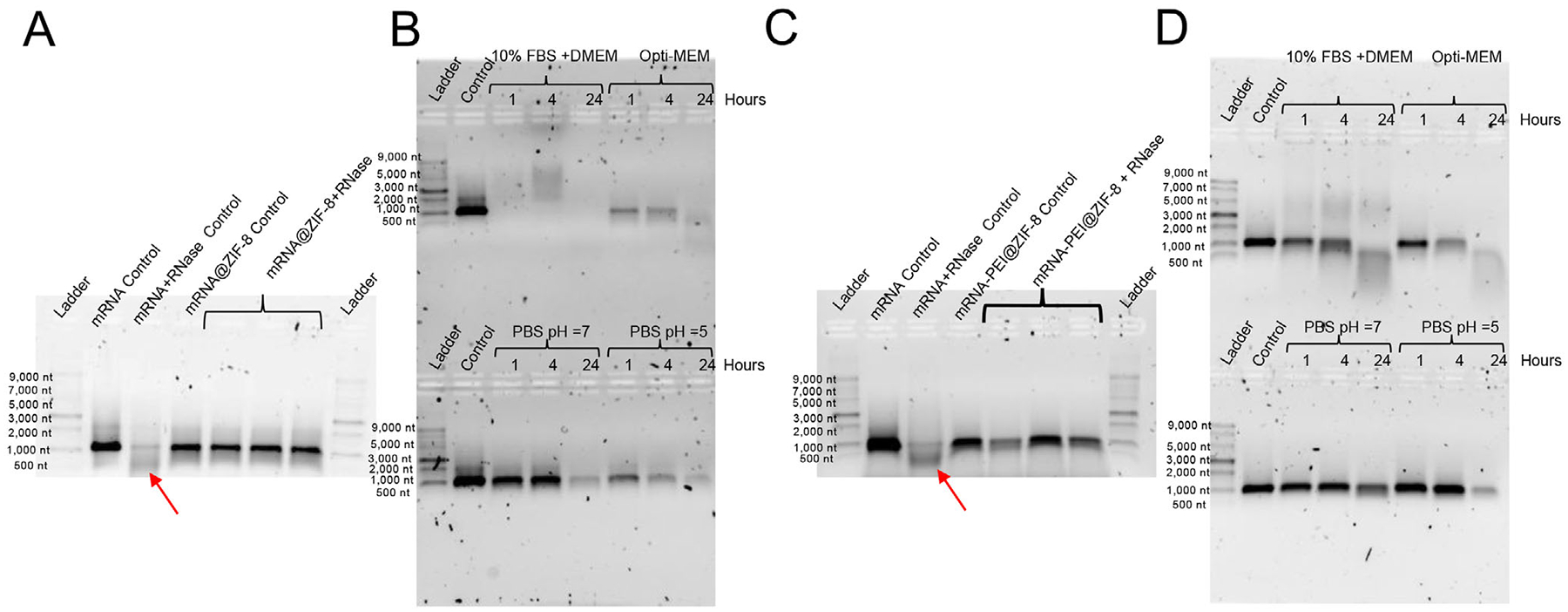
Particle protection against RNase and stability in biological mediums. A) Native RNA gel of mRNA@ZIF-8 after exposure to RNase A, compared with controls of mRNA, mRNA treated with RNase, and mRNA@ZIF-8. mRNA@ZIF-8 samples were exfoliated using EDTA before loading onto the gel. B) Native RNA gel of mRNA@ZIF-8 incubated for 1, 4, and 24 h with various biological media including DMEM culture medium with 10% FBS, Opti-MEM culture medium, and PBS at pH 7 and 5. C) Native RNA gel of mRNA-PEI@ZIF-8 after exposure to RNase A, compared with controls of mRNA, mRNA treated with RNase and mRNA-PEI@ZIF-8. mRNA-PEI@ZIF-8 samples were exfoliated using EDTA and heparin before loading onto the gel. D) Native RNA gel of mRNA-PEI@ZIF-8 incubated for 1, 4, and 24 h with various biological media including DMEM culture medium with 10% FBS, Opti-MEM culture medium, and PBS at pH 7 and 5.

As no Cy5-mRNA uptake was visible with mRNA@ZIF-8, we tested the particle’s protection against RNase and stability in biological media. After exposure to RNase A for 2 h or biological media over 24 h, particles were isolated with centrifugation. ZIF-8 was exfoliated off mRNA, and the mRNA was run on a native RNA gel (Figure 2A,B). When exposed to RNase A for 2 h suspended in PBS, mRNA@ZIF-8 demonstrates clear protection of the mRNA as the isolated mRNA matches the control band, and no degradation is visible when compared to mRNA treated with RNase A (Figure 2A). However, when mRNA@ZIF-8 is exposed to biological media, the isolated mRNA@ZIF-8 pellet yields no mRNA bands in the 10% FBS-supplemented media after 1 h of incubation (Figure 2B). When mRNA@ZIF-8 was exposed to other biological media, such as Opti-MEM and PBS (pH 7 and pH 5), mRNA isolated from the mRNA@ZIF-8 pellet showed retention of mRNA for at least 1 h. However, Opti-MEM and PBS pH 5 treatments showed significant mRNA leakage/degradation over 24 h. The medium supernatants were also tested, as in Figure S6A (Supporting Information), and confirmed that mRNA@ZIF-8 incubation with 10% FBS DMEM resulted in mRNA leakage. These findings corroborate earlier studies indicating the instability of ZIF-8 in diverse biological media. In one of those studies, Luzuriaga and collaborators demonstrated ZIF-8′s leakage in serum and serum cell culture media.^[40]^ Additionally, Spit-syna and colleagues further revealed that high salt concentrations and exposure to specific amino acids also contribute to therapeutic leakage from ZIF-8.^[39]^ Collectively, these ZIF-8 leakage studies have identified that strong inorganic, organic, and proteinaceous zinc binders cause ZIF-8 leakage and breakdown, this includes amino acids, bicarbonate, phosphates, and serum proteins.

To address the issue of mRNA leakage from ZIF-8, we introduced linear 20 kDa, polycationic PEI into the synthetic process. PEI has been utilized in several studies to improve the material loading of mRNA^[42–44]^ as well as a stand-alone commercial reagent for transfection with mixed results.^[45–50]^ PEI is also a well-known metal chelator, especially of Zn^2+^, and a ZnO and ZIF-8 particle capping agent.^[16,51–54]^ The linear 20 kDa PEI isoform was chosen because lower molecular weight and branched isoforms have proven less efficient at gene delivery for various documented reasons^[55–58]^ and are supported by our findings (Figure S7, Supporting Information). The minimum amount of PEI needed to bind all mRNA was determined through a retardation assay, where PEI and mRNA were combined at various weight ratios. The minimum binding ratio of mRNA and PEI occurred at a weight ratio of (RNA: PEI) 1:0.8, which represents a nitrogen-to-phosphate (N/P) ratio of ≈6 (Figure S8, Supporting Information, Calculation S1, Supporting Information).

With the addition of PEI, the synthetic protocol is carried out by first mixing mRNA with 2-MeIM, then adding an optimized amount of PEI (Figure S9, Supporting Information) allowing a 15-min incubation, mixing in zinc nitrate, and incubating for 2 h. This approach resembles previously employed one-pot coprecipitation methods^[16,24]^ with the distinction that PEI is electrostatically complexed with mRNA before ZIF-8 encapsulation, creating a ZIF-8 shell around the mRNA-PEI complex (mRNA-PEI@ZIF-8) (Scheme 1A). By creating an mRNA-PEI complex core, the mRNA is retained in the core while PEI aids in the stability of the ZIF-8 coating, possibly due to its strong binding of Zn ions with uncomplexed or partially complexed PEI.

The generated composite particles, mRNA-PEI@ZIF-8 (mRPZ), underwent treatment with RNase A and exposure to biological media (Figure 2C,D). When comparing mRPZ to RNase-treated mRNA, minimal to no degradation of the mRNA was observed, as the band remained approximately the same length as the RNA control. This validates that mRPZ shields the mRNA from RNase as well as mRNA@ZIF-8, a crucial requirement for effective gene delivery. Following exposure to biological media, the isolated mRPZ pellet was analyzed on a native RNA gel, revealing a distinct band of mRNA present for all biological media for up to 4 h. This confirms that mRPZ is stable longer than mRNA@ZIF-8 in biological media. This improved stability can be attributed to the incorporation of PEI as the mRNA@ZIF-8 alone was not stable.

To further assess particle stability under physiologically relevant conditions, mRNA@ZIF-8 and mRPZ encapsulating Cy5-tagged mRNA were suspended in phosphate-buffered saline (PBS) supplemented with 10% fetal bovine serum (FBS) to simulate serum-containing environments. Samples were incubated over a 24 h period, and supernatants were collected at defined time points to quantify mRNA release, as measured by the Cy5 fluorescence signal. The cumulative release profiles (Figure S10, Supporting Information) reveal that mRNA@ZIF-8 undergoes rapid degradation, with over 75% of the encapsulated mRNA released within the first 4 h. In contrast, mRPZ exhibited a markedly slower release, with only 19% of total mRNA released over 24 h. These findings highlight the limited stability of mRNA@ZIF-8 in serum-containing media and demonstrate that PEI incorporation in mRPZ significantly enhances particle stability and mitigates premature mRNA release.

The development of an effective mRNA delivery platform using ZIF-8 required a thorough evaluation of synthesis conditions compatible with mRNA stability, integrity, and expressibility. Our findings highlight the unique challenges associated with encapsulating large, sensitive nucleic acids such as mRNA within MOFs. While ZIF-8 has been widely studied for drug delivery, our study reveals that standard biomimetic mineralization conditions using aqueous solvents are insufficient for consistent nanoparticle formation and do not adequately preserve mRNA structure. Methanol-based conditions led to significant degradation of mRNA, while water-based encapsulation resulted in particles with inconsistent morphology and crystalline structure. In contrast, ethanol-based coprecipitation was identified as the most suitable approach, yielding uniform particles that preserved mRNA length and expression capacity. However, despite successful encapsulation and structural retention, mRNA@ZIF-8 failed to mediate transfection, likely due to instability in biological environments and insufficient protection against premature cargo release.

To overcome these limitations, we incorporated linear 20 kDa PEI into the synthetic process, generating a composite system (mRPZ). PEI not only forms electrostatic complexes with mRNA to improve encapsulation but also acts as a stabilizing agent for the ZIF-8 shell, potentially through strong coordination with Zn^2+^ ions. This modification markedly improved and prolonged particle stability in serum-containing media. Compared to mRNA@ZIF-8, which released over 75% of its cargo within 4 h, mRPZ demonstrated a controlled release profile with less than 20% mRNA release over 24 h. These enhancements suggest that PEI may mitigate destabilizing effects of serum proteins and other zinc-coordinating biomolecules that typically compromise ZIF-8 integrity. This is consistent with prior reports that identify phosphate, bicarbonate, amino acids, and serum proteins as key drivers of ZIF-8 degradation in physiological conditions.^[39,40]^

In summary, our synthesis optimization established a robust one-pot coprecipitation strategy that supports mRNA integrity, facilitates encapsulation, and enhances stability in biological media. Ethanol was identified as the optimal solvent, and PEI incorporation proved critical to overcoming the inherent limitations of ZIF-8 for mRNA delivery. The resulting mRPZ particles demonstrate promising material properties for therapeutic application. With this optimized formulation in hand, we next characterized the physicochemical structure of mRPZ and evaluated its biological performance in vitro and in vivo.

### 3.2. Characterization

**Figure 3.**
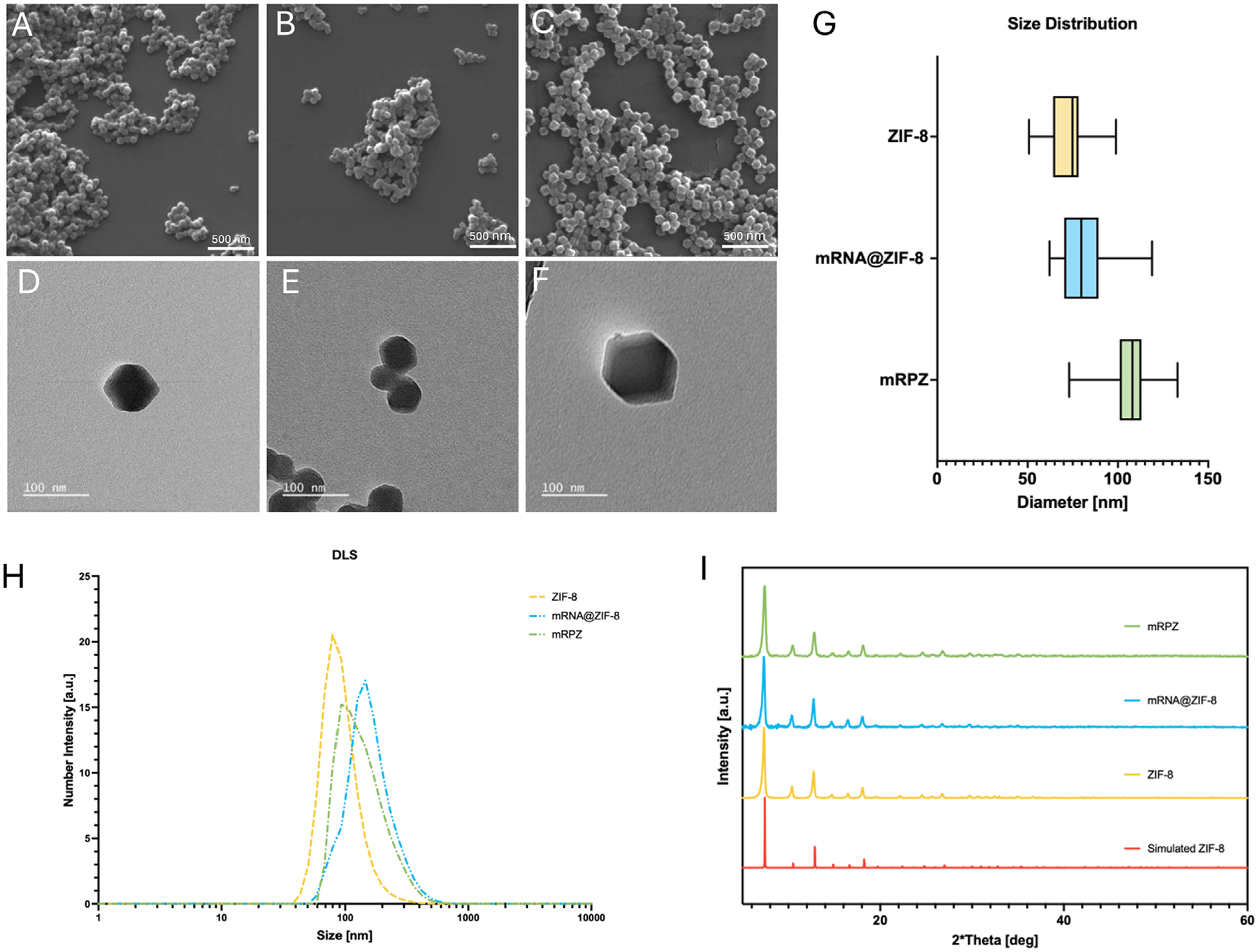
Characterization of ZIF-8, mRNA@ZIF-8, and mRPZ nanoparticles. SEM and TEM images of A,D) ZIF-8, B,E) mRNA@ZIF-8, and C,F) mRPZ (scale bars = 500 and 100 nm, respectively). Size distribution as measured by G) SEM and H) hydrodynamic diameter measured by DLS. I) Powder X-ray Diffraction of synthesized particles. Box and whisker plot represents the minimum, 25^th^ percentile, median, 75^th^ percentile, and maximum with a sample size of N = 25 for ZIF-8 and N = 50 for mRNA@ZIF-8 and mRPZ.

Synthesized mRPZ was imaged with SEM and TEM along with literature-synthesized ZIF-8 and mRNA@ZIF-8 (Figure 3A–F; Figure S11, Supporting Information). mRPZ features the distinct dodecahedron morphology that is present in the ZIF-8 literature. Using imaging analysis on acquired SEM images, mRPZ was ≈107 nm, larger than literature-synthesized ZIF-8 and mRNA@ZIF-8, 74 and 80 nm, respectively (Figure 3G). Some variation in size occurred from batch to batch, see Figure S12 (Supporting Information). The mean hydrodynamic diameter, as determined by dynamic light scattering (DLS), of mRPZ was 146 nm (Figure 3H) which may be explained by uncomplexed PEI. Interestingly, the hydrodynamic diameter of mRNA@ZIF-8 was larger than mRPZ and literature-synthesized ZIF-8, contrary to the size obtained with SEM (Δd = 80 nm larger). The difference in diameter may indicate that mRNA is loosely associated with the surface of the mRNA@ZIF-8 particle, which increases its hydrodynamic diameter and is later confirmed with a molecular probing technique. PXRD confirmed the synthesis of the ZIF-8 particles. The diffraction patterns of mRPZ and mRNA@ZIF-8 match well with simulated ZIF-8 and literature-synthetized ZIF-8 peaks (Figure 3I).

**Figure 4.**
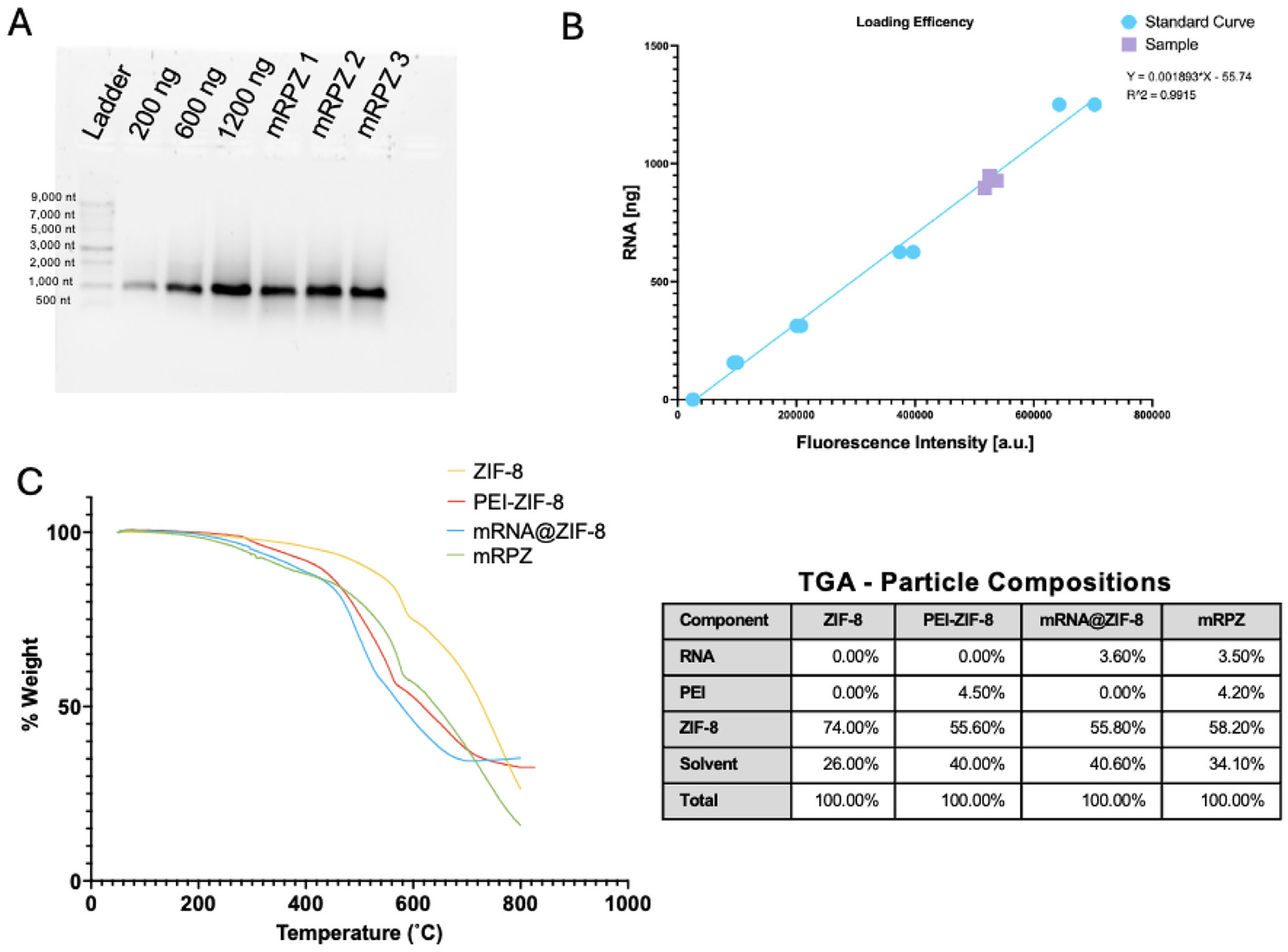
Evaluation of mRNA loading efficiency and capacity of mRPZ. The loading efficiency of mRPZ was determined by image analysis of A) a native RNA gel and B) a fluorescent probe. The particle composition and loading capacity were further elucidated through C) thermogravimetric analysis (TGA).

Further characterization of this novel particle was undertaken to ascertain mRNA loading within mRPZ and to assess the particle’s composition. Loading efficiency and loading capacity are crucial parameters in nucleic acid delivery, representing the efficiency of mRNA encapsulation and the quantity of mRNA the material can accommodate, respectively. Elevated loading efficiencies and capacities minimize the waste of unloaded therapeutic material, reduce the required exogenous materials for mRNA delivery, and lower the risk of systemic toxicity. The loading efficiency of mRPZ was measured as the amount of RNA released from nanoparticles after exfoliation of ZIF-8 with EDTA and dissociation from PEI with heparin sulfate (Figure S2B, Supporting Information) divided by the mRNA used in synthesis. Gel electrophoresis and ImageJ analysis were utilized to compare band intensities of released RNA to standards. The loading efficiency of mRPZ and mRNA@ZIF-8 was found to be 91.1% ± 7.7% and 91.5% ± 9.5%, respectively (Figure 4A; Figures S13 and S14, Supporting Information). While Li et al. reported increased loading efficiency with the addition of PEI, our study did not yield statistically different results. The loading efficiency of mRPZ was further validated using a fluorescent probe assay, confirming a similar loading capacity of 92.3% ± 2.5% (Figure 4B).

The loading capacity of mRPZ was determined through thermogravimetric analysis, where the organic components of mRPZ exhibit lower burning temperatures than ZIF-8.^[24,59]^ Differential mass loss over a temperature range helped identify the weight composition of each particle component. Weight compositions of mRNA@ZIF-8, PEI-ZIF-8, and ZIF-8 were employed to confirm the decomposition of each component (Figure 4C; Figure S15, Supporting Information). From the literature RNA, PEI, and ZIF-8, decompose between 100–300 °C,^[60]^ 300–400 °C,^[61]^ and 400 °C-600 °C^[59]^ under nitrogen, respectively. The mRNA loading capacity of mRPZ was calculated to be 5.3%, which is considered high compared to previous work loading high molecular weight nucleic acids in MOFs.^[15,16]^ The loading capacity for mRNA@ZIF-8 was slightly higher at 6.0%, possibly due to the absence of PEI contributing to the overall weight. Intriguingly, the incorporation of mRNA and PEI within ZIF-8 reduced its decomposition temperature, evidenced by shifts in the composite ZIF-8 materials. This phenomenon aligns with observations made by Kang et al. in their study involving coprecipitation of PVP and Urease with ZIF-8.^[24]^ FTIR was also performed to ensure chemical consistency between tested samples and ZIF-8 (Figure S16, Supporting Information).

**Figure 5.**
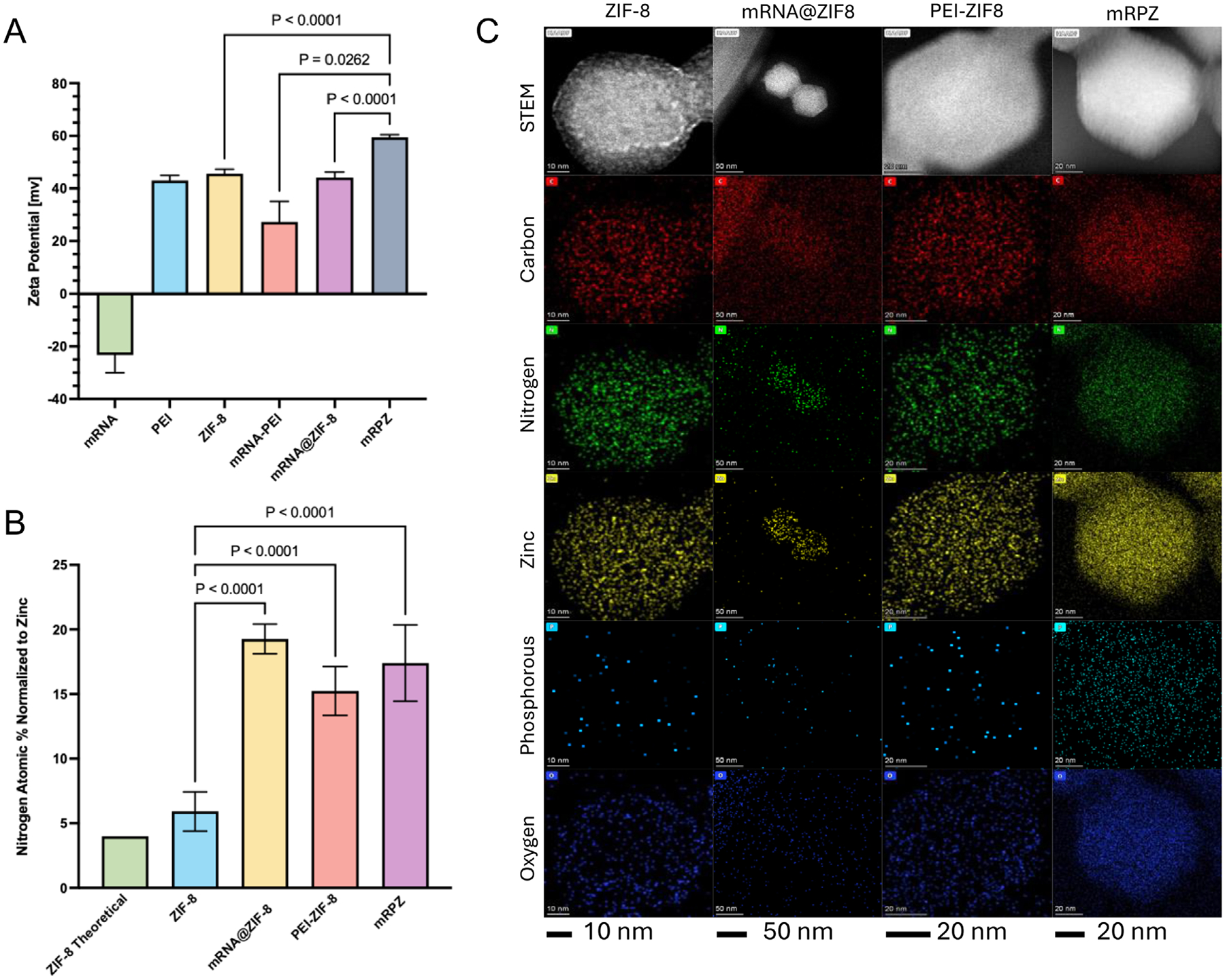
The incorporation of mRNA inside ZIF-8 was confirmed using A) zeta potential differences and B) EDS. Element mapping with STEM/EDS demonstrates mRNA encapsulation within mRPZ C). Scale bars for STEM/EDS are 10, 50, 20, and 20 nm respectively. Zeta potential data presented as mean ± StdEM, sample sizes are N ≥ 3, and statistical significance (P) was assessed with a Browne-Forsythe and Welch one-way ANOVA. EDS data presented as mean ± standard deviation (SD), sample sizes are N ≥ 10, and statistical significance (P) was assessed with one-way ANOVA.

To validate the integration of mRNA with ZIF-8, a comprehensive analysis involving zeta potential measurements, energy dispersive spectroscopy (EDS), and a molecular probe assay was conducted. Zeta potential assessments were carried out for individual components, as well as the composite mRPZ and mRNA-PEI nanoparticles (Figure 5A; Figure S17, Supporting Information). The binding of PEI with mRNA to form mRNA-PEI (27.3 mV) resulted in a discernible reduction in zeta potential compared to PEI alone (43.1 mV). Subsequent coating with ZIF-8 led to a significant increase in the zeta potential of the particle composite (mRPZ), reaching 59.5 mV, which is greater than ZIF-8 alone (45.63 mV). This observation provides compelling evidence of the incorporation of mRNA-PEI within a ZIF-8 shell.

EDS was employed to affirm the presence of PEI and mRNA within ZIF-8, manifested by an increase in the nitrogen: zinc atomic weight percent, as the composite particles contain PEI and mRNA, which add to the nitrogen content of the entire particle. The atomic weight compositions were determined at various locations within the bulk sample (Figure S18, Supporting Information). Our findings indicate a distinct rise in nitrogen atomic percent when comparing ZIF-8 with mRPZ, signifying the incorporation of mRNA and PEI into the bulk material (Figure 5B). Other tested composite materials, namely PEI-ZIF-8 and mRNA@ZIF-8, also showed increased nitrogen: zinc weight percent compared to ZIF-8. Qualitative element mapping was performed using an EDS-equipped STEM/TEM (Figure 5C). Due to the sensitive nature of ZIF-8 to the electron beam damage, the measured atomic percentages obtained with TEM were not consistent with SEM or literature (Figure S19A, Supporting Information). However, there is still a notable increase in the zinc nitrogen ratio of mRPZ compared to ZIF-8 as well as visible signatures of phosphorous in mRPZ.

**Figure 6.**
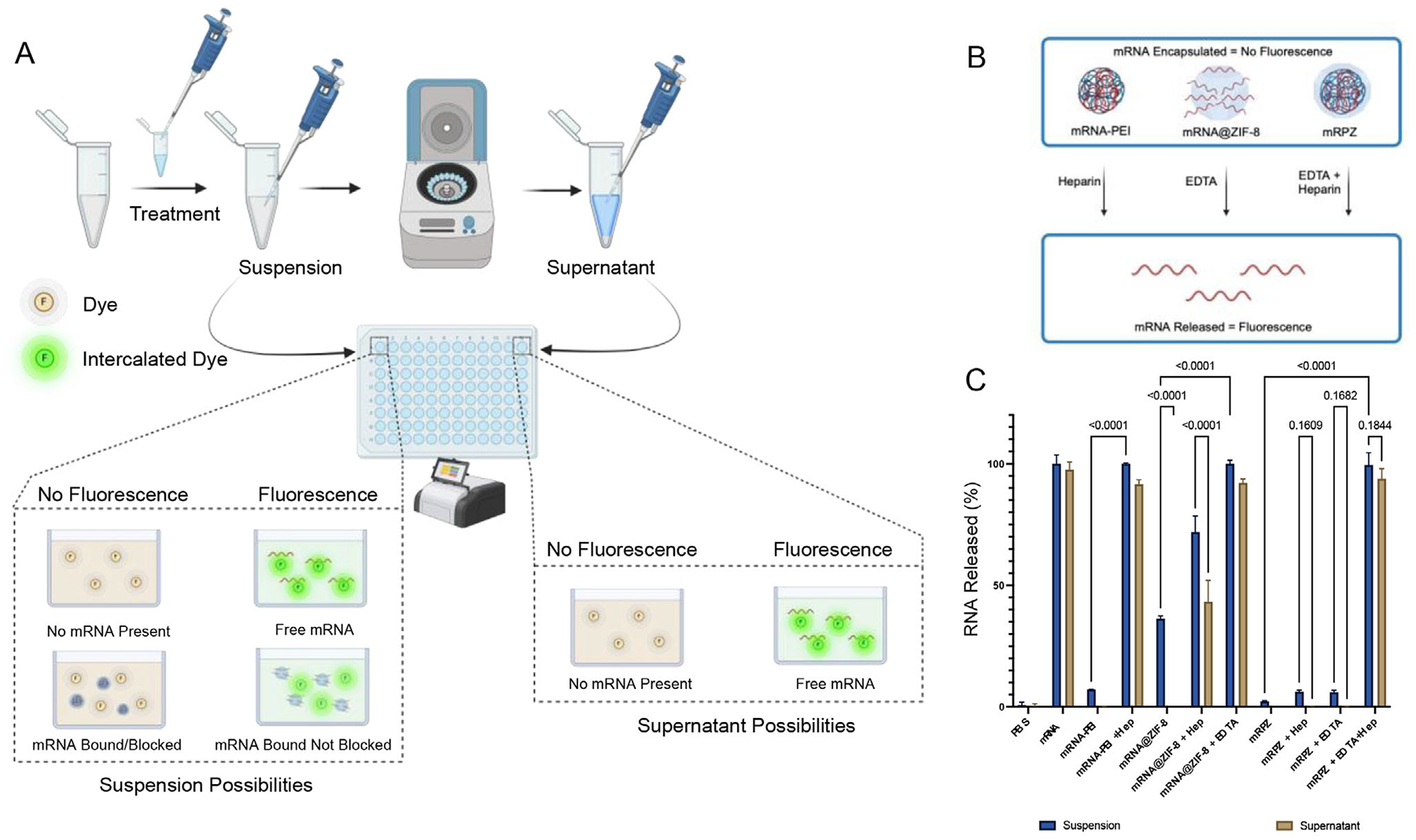
Schematic of molecular probe assay A), schematic of treatments to particles B), and resulting mRNA release measured by fluorescent probe C). Data presented as mean ± StdEM, sample sizes are N = 6, and statistical significance (P) was assessed with two-way ANOVA.

To further establish the incorporation of mRNA-PEI within ZIF-8, a molecular probe assay utilizing an intercalating dye was employed, emitting fluorescence upon contact with free mRNA or bound mRNA which is not blocked from the intercalating dye (Figure 6A). By treating mRPZ, mRNA-PEI, and mRNA@ZIF-8 with EDTA and heparin, followed by probing with the intercalating dye, how mRNA is incorporated into mRPZ could be elucidated (Figure 6B). EDTA served to exfoliate ZIF-8, while heparin displaced or released mRNA from electrostatically binding surfaces or molecules, the surface of ZIF-8 (+45.6 mV) or PEI in this case. Briefly, particles were suspended in PBS and treated with EDTA and heparin, separately or together, or received no treatment. The suspension was probed with dye and then centrifuged to pellet any suspended particles. After centrifugation, the supernatant fluid, which could contain free mRNA, was probed with dye. By probing both the suspension and the supernatant after centrifugation, it was possible to determine if mRNA was released from the particle or bound to the particle. The data (Figure 6C) indicate that mRNA is encapsulated within mRPZ, as treatment with heparin alone did not fully release RNA into the supernatant or suspension, akin to mRNA-PEI. Conversely, treatment with EDTA alone did not cause the release of mRNA from mRPZ, as observed with mRNA@ZIF-8. Only the combined treatment of mRPZ with EDTA and heparin resulted in the unbinding and release of mRNA into the supernatant. Interestingly, mRNA@ZIF-8 did have a signal without treatment and with heparin treatment. This alludes to some mRNA loosely associating with the surface of mRNA@ZIF-8, which could explain its larger hydrodynamic diameter. Additionally, this loosely associated mRNA appears to dislodge from mRNA@ZIF-8 when treated with heparin, explaining the signal increase in the heparin-treated suspension and supernatant fractions. Controls confirm no fluorescence is caused by interactions with ZIF-8, PEI, EDTA, and Heparin (Figure S20, Supporting Information).

The successful synthesis and characterization of mRPZ demonstrate the development of a structurally distinct and compositionally robust nanoparticle platform for mRNA delivery. Imaging by SEM and TEM confirmed that mRPZ maintains the characteristic dodecahedral morphology associated with ZIF-8, while displaying a larger average particle size (107 nm) compared to literature-synthesized ZIF-8 and mRNA@ZIF-8. This size increase likely reflects the incorporation of the polymer-RNA core within the ZIF-8 shell. Notably, dynamic light scattering measurements revealed that mRPZ exhibited a smaller hydrodynamic diameter than mRNA@ZIF-8, suggesting a more compact internal structure and reduced surface-bound mRNA. This structural distinction is further supported by molecular probe assays indicating that, unlike mRNA@ZIF-8, mRPZ tightly encapsulates its mRNA cargo rather than permitting surface association.

Crystallinity analysis by PXRD confirmed that the ZIF-8 structure is preserved in mRPZ, while thermogravimetric analysis revealed a substantial mRNA loading capacity of 5.3%, among the highest reported for MOF-based nucleic acid carriers.^[8,15,16]^ Gel electrophoresis and fluorescent probe assays further validated high loading efficiencies exceeding 90%, comparable to or better than previous systems.^[8,16]^ Elemental analysis via EDS and zeta potential measurements provided additional confirmation of successful PEI and mRNA incorporation into the ZIF-8 framework. An increased nitrogen-to-zinc ratio, along with the detection of phosphorous signals in mRPZ, and a significant rise in surface charge relative to ZIF-8 alone, support the conclusion that a positively charged mRNA-PEI complex is fully embedded within the MOF structure.

The molecular probe release study provided critical insight into the nature of mRNA incorporation. While mRNA@ZIF-8 exhibited substantial mRNA release upon heparin treatment alone, consistent with loosely bound surface mRNA, mRPZ required the combined action of EDTA (to chelate and dissolve the ZIF-8 shell) and heparin (to dissociate mRNA from PEI) to release the RNA. This confirms a true core-shell architecture in which the mRNA-PEI complex is protected within the ZIF-8 matrix.

Together, these results establish mRPZ as a uniquely structured, high-loading mRNA delivery system that overcomes several limitations of conventional ZIF-8 and PEI carriers. Its compact morphology, efficient encapsulation, protective ZIF-8 coating, and high loading capacity suggest improved performance in biological environments. Given these promising material properties, we next evaluated the biological activity of mRPZ through in vitro transfection studies in multiple mammalian cell lines, followed by in vivo investigations to explore its potential for therapeutic mRNA delivery applications.

### 3.3. In Vitro Application

**Figure 7.**
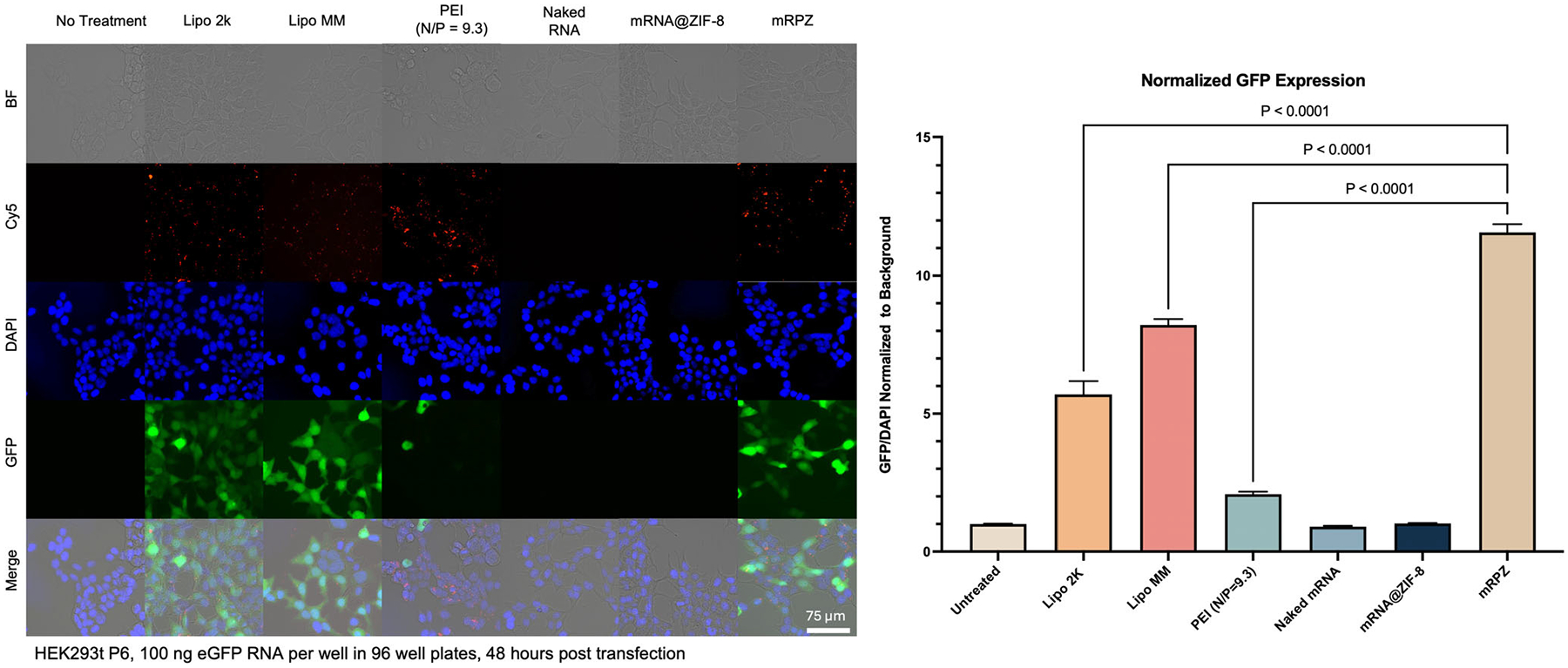
Uptake and eGFP expression of mRPZ by HEK293t cells are illustrated through Cy5 and eGFP signals. Fluorescence microscopy was used for visualization **(Left)** and fluorescence signals were further quantified **(Right)**. Scale bar = 75 μm. Data presented as mean ± StdEM, sample sizes are N ≥ 32, and statistical significance (P) was assessed using a one-way ANOVA.

Along with rigorous characterization, mRPZ was applied for in vitro cell transfection. Briefly, Cy5-tagged, eGFP-encoding mRNA-loaded mRPZ nanoparticles were suspended in Opti-MEM and administered to HEK293t in serum-containing media. After 48 h, cells were fixed and stained with a nuclear dye (DAPI), and the resulting fluorescence was imaged with a microscope and quantified with a plate reader (Figure 7; Figure S21, Supporting Information). The presence of Cy5 located within the fixed cells, coupled with the expression of eGFP, indicates the successful delivery of mRNA by mRPZ and the subsequent expression of its corresponding protein. For comparison, cells treated with mRNA@ZIF-8 are absent of Cy5 and eGFP signals. Given that PEI is a known transfection reagent, mRNA-PEI transfection was also performed as a comparison, utilizing the same nitrogen-to-phosphate ratio of 9.3 as mRPZ. Interestingly, mRPZ performs five to ten times better than mRNA-PEI (PEI N/P = 9.3) in HEK293t, HeLa, and CHO cells (Figure S22, Supporting Information). Specifically in HEK cells, mRPZ utilizing an N/P ratio of 9.3 outperforms mRNA-PEI complexes with an optimized N/P ratio of 28–30 (Figure S7, Supporting Information), which is consistent across PEI isoforms (Figure S23, Supporting Information). This performance increase may be attributed to the release of ZIF-8 precursors during endosomal compartment acidification, which shield the polycationic charge of PEI and enhance mRNA release.^[56]^ Moreover, PEI’s ability to disrupt endosomal membranes by forming stable pores and defects likely facilitates cytosolic delivery and improves transfection efficiency of the mRPZ platform.^[62]^ Additionally, across all three cell lines, mRPZ outperforms Lipofectamine 2000 (Lipo 2k), and mRPZ performs better than or similar to Lipo MM, except in CHO cells.

**Figure 8.**
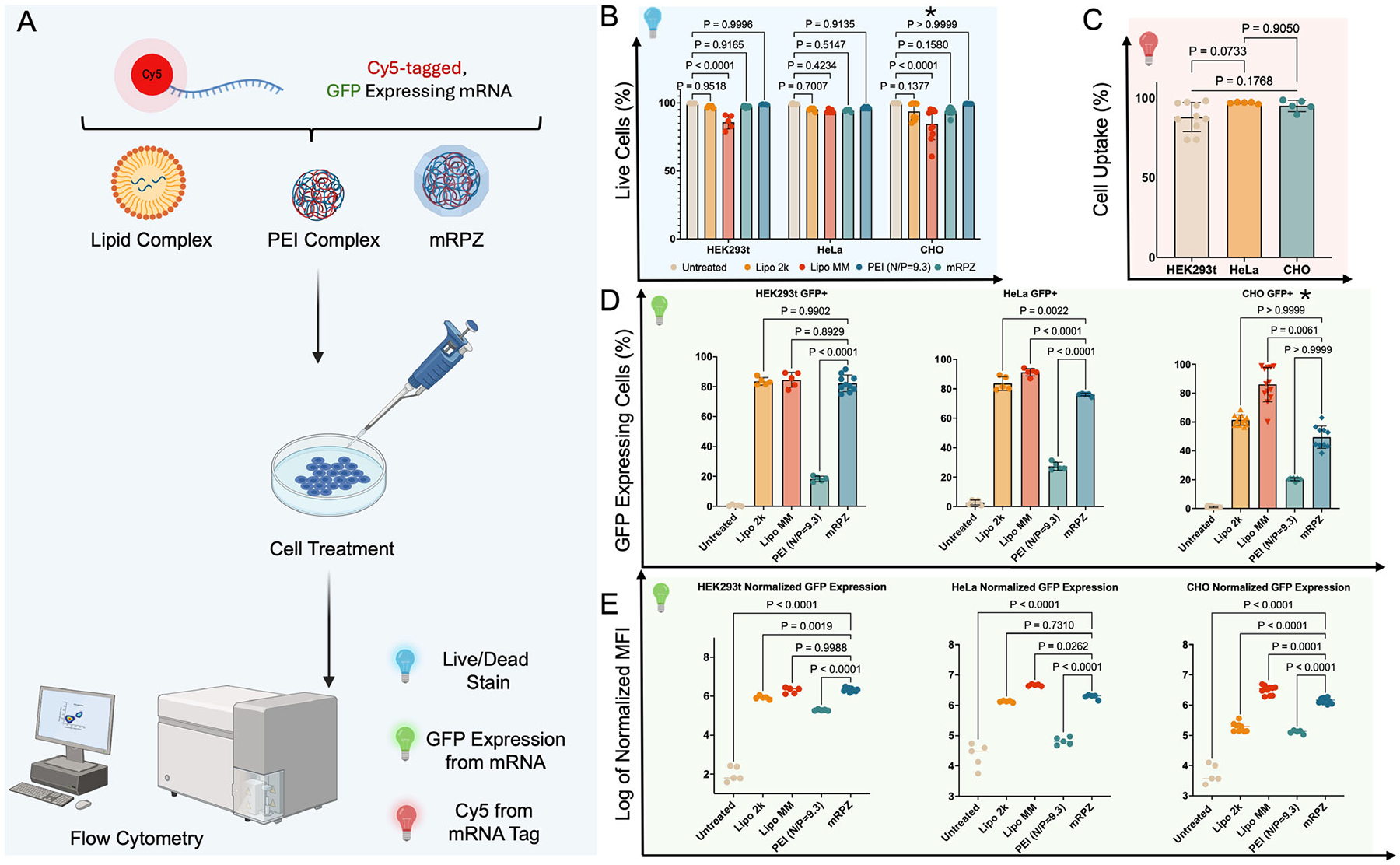
Schematic of flow cytometry workflow A). Quantitative single-cell measurements using flow cytometry for B) cell viability, C) cellular uptake, D) cellular expression mRNA in live cells, and E) Normalized median fluorescence index (MFI). Data presented as mean ± SD, samples sizes were N ≥ 5, and statistical significance (P) assessed with one-way ANOVA unless noted. Asterisks (*) denote statistical significance tests that were run with parametric comparisons due to non-normally distributed data. Biological replicates performed.

To more quantitatively assess the uptake and expression of mRPZ particles, Cy5-labeled, eGFP-encoding mRPZ nanoparticles and Lipofectamine were incubated with HEK293t, HeLa, and CHO cells for 48 h, and the resultant cells were evaluated by flow cytometry and imaged (Figures S24–S27, Supporting Information). Cell viability was assessed using an amine-reactive dye, LIVE/DEAD^™^ Fixable Blue, and cell uptake was assessed using the Cy5 mRNA tag (Figure 8A). As shown in Figure 8B,C, the cellular viability and uptake of all cell lines were above 92% (except Lipo MM) and 88%, respectively. This demonstrates that mRPZ is efficiently taken up by cells and causes minimal toxicity. Comparing the relative eGFP expression in all cell lines, mRPZ induces eGFP expression in 82.2%, 76.1%, and 49.5% of live cells for HEK293t, HeLa, and CHO cells, respectively (Figure 8D). In comparison to positive Lipofectamine controls, mRPZ performs equally well or slightly worse depending on the cell line and the specific Lipofectamine control. Interestingly, the Median Fluorescence Index (MFI), normalized with the percentage of GFP-positive cells, shows mRPZ performs similarly to Lipofectamine controls on a single-cell basis, except in comparison to Lipo MM in CHO cells (Figure 8E). mRPZ also outperforms mRNA-PEI at an N/P ratio of 9.3 and outperforms or equivalent performs to mRNA-PEI at an N/P ratio of 28 (PEI N/P = 28) in terms of normalized MFI and percentage of GFP positive cells (Figures S25–S27, Supporting Information). The normalized MFI measurement captures the intensity of eGFP expression on a single-cell basis. The increased MFI caused by mRPZ may allude to more mRNA strands being delivered into a single cell. Using nanoparticle tracking to obtain particle concentrations, we backcalculated the number of mRNA strands per nanoparticle to be ≈33 (Figure S28, Supporting Information). Compared to known lipid nanoparticles, this is 11 to 16-fold more strands per particle,^[10]^ providing some reasoning as to why the percentage of Cy5 and GFP positive cells are lower for mRPZ, but normalized MFI is higher or equivalent. Additional cell lines, RAW264.7, A549, and 231, were tested using mRPZ (Figures S29–S31, Supporting Information). Generally, we find mRPZ underperforms compared to commercial lipid complexes, except in RAW 264.7 mouse macrophages where they perform equivalently to Lipo 2k. In comparison to mRNA-PEI, mRPZ outperforms or equivalently performs mRNA-PEI at both N/P ratios of 9.3 and 28.

The toxicity of mRPZ, components, and commercial transfection reagents were assessed using a Resazurin assay which measures cell metabolic activity. The viability of the cells after transfection with Lipo 2K, Lipo MM, mRNA-PEI (N/P = 28), and mRPZ were not statistically different from the untreated cells (Figure S32A, Supporting Information). Cells treated with Linear 20 kDa PEI (Figure S32B, Supporting Information) showed no signs of toxicity up to a concentration of 3.6 μg mL^−1^, which corresponds to transfecting with PEI at an N/P ratio of ≈28. At higher concentrations, concentrations exceeding 12 μg mL^−1^, PEI significantly reduces the metabolic activity of the cells. Interestingly, cells treated with 3.6 μg mL^−1^ of PEI and transfected with mRNA-PEI at N/P ratios of ≈28 showed visible signs of reduced growth, as final cell confluency was lower compared to other treatments. Cells treated with ZIF-8 (Figure S32C, Supporting Information) showed no significant metabolic change up to 50 μg mL^−1^. For reference, mRPZ used to transfect one 96-well plate well would correspond to 16.62 μg mL^−1^ of ZIF-8. Higher concentrations of ZIF-8 cause a significant drop in metabolic activity and cause cell morphology to become round. The combination of PEI and ZIF-8 interestingly shows better transfection over PEI alone and reduces the amount of PEI needed.

We further studied the kinetics of eGFP expression after transfection by treating HEK293t cells with mRPZ and monitoring the eGFP fluorescence in phenol red-free media for 96 h with a plate reader (Figure S33, Supporting Information). mRPZ reaches a maximum eGFP signal at 72 h and reaches 80% of its maximum expression at 44 h. Lipo 2k and Lipo MM reach maximum expression ≈48 h and reach 80% of their maximum expression ≈24 h. mRNA-PEI (PEI N/P = 9.3) reaches its maximum expression at 96 h and 80% of its maximum expression at ≈21 h. This delayed expression may be explained by the slow dissociation of the ZIF-8 coating from mRPZ. Similar delayed expression has been observed in studies with pDNA and ZIF-8 bio-composites.^[8,16,32]^ Interestingly, our synthetic methodology for mRNA encapsulation also works for pDNA. After encapsulation in ZIF-8 using our coprecipitation method, pDNA-PEI@ZIF-8 (pDPZ) was imaged using SEM (Figure S34A, Supporting Information) and applied for the transfection of HEK293t cells (Figure S34B, Supporting Information). The eGFP expression in the cells was observed at 48 and 96 h (Figure S34C, Supporting Information). Interestingly, similar to mRPZ and previous reports, the expression of the eGFP-pDNA was also delayed, this time by 48 h. The success of our synthetic methodology with pDNA (6.1 kbp) delivery may translate for larger mRNA.

**Figure 9.**
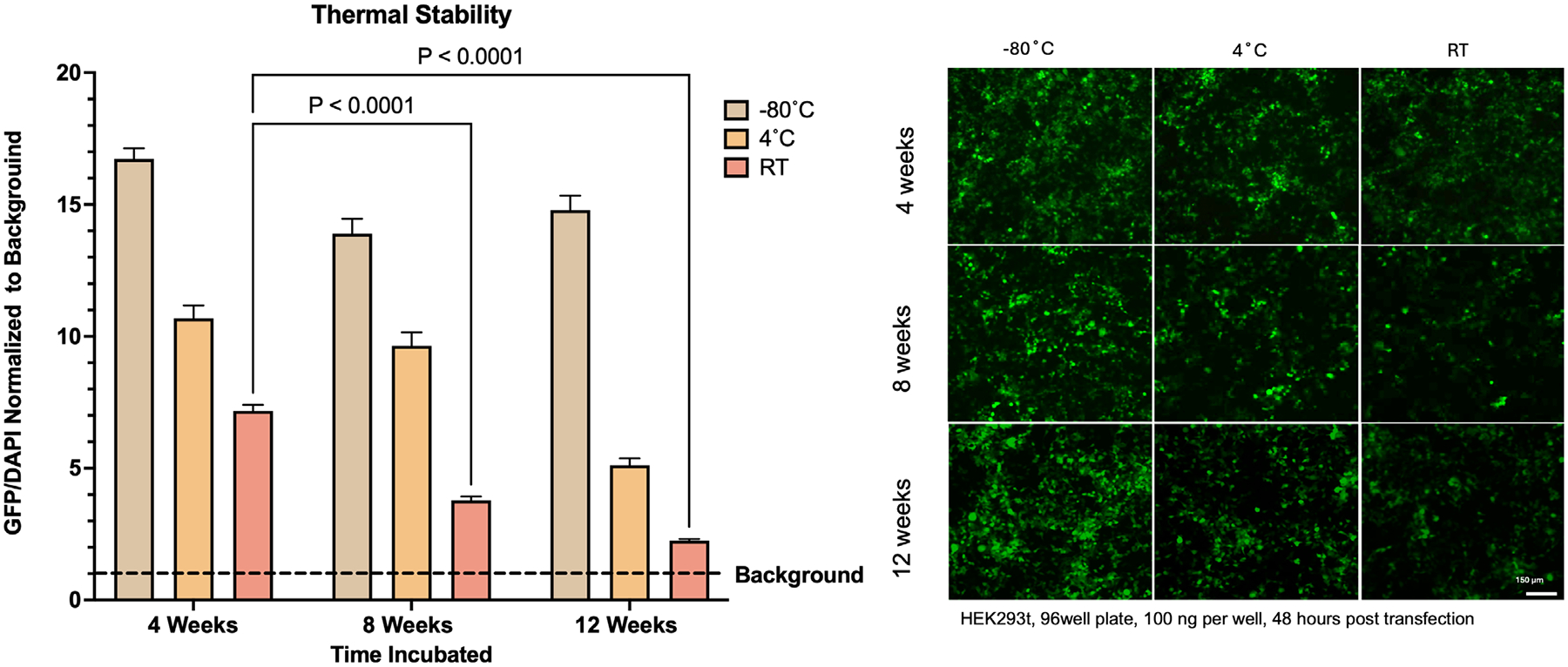
eGFP expression after transfecting HEK293t cells using mRPZ nanoparticles that were stored at −80 °C, 4 °C, and room temperature (RT) and for various times. Scale bar = 150 μm. Data presented as mean ± StdEM, sample sizes N ≥ 32, and statistical signficance (P) assessed with two-way ANOVA. Biological replicates performed.

The stability of an mRNA delivery system is critical for its practical applications. The greatest bottleneck for mRNA vaccines during the COVID-19 pandemic was the need for extremely cold storage temperatures in shipping.^[63]^ These temperatures were needed to ensure the viability of mRNA in the vaccine. Here in, we report the first long-term stability of mRNA encapsulated inside MOFs at various temperatures. We investigated the stability of the encapsulated mRNA over 12 weeks at room temperature, 4 °C, and −80 °C by taking mRPZ, drying it for 1 hour, and storing it. Room temperature storage condition was under vacuum as particles stored without vacuum had little transfection viability and formed cavities visible under TEM (Figure S35, Supporting Information). We believe the cavity formation is linked to the degradation of the mRNA, further validating the theory of a core-shell MOF particle. Interestingly, we found that after one month, the encapsulated mRNA transfected well across all temperatures, producing eGFP signals that were several folds above an untreated background sample (Figure 9). However, at 8 and 12 weeks, a significant drop in the eGFP signal was observed in mRPZ stored at 4 °C and room temperature. mRPZ maintained its ability to successfully transfect cells even after 12 weeks at room temperature. When focusing on the stability of mRNA in mRNA-PEI versus mRPZ, a clear degradation of mRNA in mRNA-PEI was evident by 8 weeks, rendering it unable to transfect cells regardless of storage time or temperature (Figures S36–S38, Supporting Information). The material stability of mRPZ was recorded over 3 months and compared to mRNA@ZIF-8 (Figure S39, Supporting Information). Notably, mRPZ and mRNA@ZIF-8 demonstrate a similar size and morphology at 1 month but more round morphologies at 3 months. The crystallinity was assessed using PXRD. mRPZ and mRNA@ZIF-8 demonstrate retention of the ZIF-8 diffraction pattern.

When comparing mRNA stored under the same thermal conditions and delivered with Lipo MM with mRPZ, we observed that mRNA was stable and able to transfect regardless of the time or temperatures tested. Therefore, we confirm the ZIF-8 coating on mRPZ does improve the stability of the mRNA-PEI complex but does not aid the stability of the mRNA for cell transfection. Interestingly, mRNA from mRNA@ZIF-8 stored at room temperature, exfoliated, and transfected using Lipo MM does illicit eGFP expression but is much lower than mRPZ or other Lipofectamine controls with fresh mRNA. Interestingly, these findings do not support the idea that ZIF-8 can thermally stabilize all biomolecules (specifically mRNA); however, the coprecipitation methods used here differ from the biomimetic mineralization methods used in previous works.^[14]^

The in vitro evaluation of mRPZ nanoparticles highlights their significant potential as an mRNA delivery platform. Fluorescence imaging and flow cytometry confirmed that mRPZ enables efficient cellular uptake and strong eGFP expression across multiple mammalian cell lines, outperforming mRNA@ZIF-8, mRNA-PEI complexes, and in some cases, commercial lipid-based transfection reagents. Specifically, mRPZ exhibited five- to tenfold greater transfection efficiency compared to mRNA-PEI at both conventional (N/P = 9.3) and optimized (N/P = 28–30) ratios. The mRPZ particles demonstrated high cellular uptake (>88%) and minimal cytotoxicity (>92% viability) in HEK293t, HeLa, and CHO cells, offering a favorable safety profile. The increased median fluorescence intensity per transfected cell suggests that the high mRNA loading capacity of mRPZ (≈33 strands per nanoparticle) contributes to enhanced intracellular protein expression, supporting the effectiveness of the platform at the single-cell level.

The superior performance of mRPZ can be attributed to the compounding effects of its structural components. Upon endosomal acidification, the partial dissolution of ZIF-8 releases zinc and imidazole precursors, which electrostatically shield the highly cationic PEI, promoting the release of mRNA into the cytoplasm.^[16,56]^ Additionally, PEI is known to destabilize lipid bilayers by stabilizing spontaneous membrane defects, facilitating the formation of stable pores or channels that allow for efficient mRNA escape into the cytosol.^[62]^ This combination of charge shielding and membrane destabilization likely enhances both the release and cytoplasmic availability of the delivered mRNA, leading to higher transfection efficiency and protein expression compared to PEI alone or ZIF-8 encapsulation alone. Moreover, the delayed kinetics of gene expression, reaching maximum eGFP levels at ≈72 h, aligns with the gradual dissolution of the ZIF-8 shell and controlled release of the mRNA cargo, offering potential benefits for applications requiring sustained gene expression.^[8]^

Importantly, storage stability studies demonstrated that mRPZ particles preserved functional mRNA delivery after 12 weeks at room temperature under vacuum, significantly outperforming mRNA-PEI complexes, which lost transfection capability by eight weeks. While ZIF-8 encapsulation enhanced the stability of the mRNA-PEI complex, it did not fully prevent mRNA degradation for direct lipid-mediated transfection, suggesting that the primary stabilization benefit lies in maintaining the structural integrity of the PEI-mRNA association. This enhanced stability may be attributed to the ZIF-8 shell physically supporting the macro-molecular structure of the mRNA-PEI complex—consistent with observations reported by Liang and colleagues with proteins—and to the ability to dry the particles without loss of function.^[14]^ Furthermore, the versatility of the mRPZ platform was demonstrated by its successful application to plasmid DNA delivery, highlighting its potential for broader nucleic acid therapeutic applications.

Given the strong in vitro performance, high loading capacity, controlled release behavior, and improved storage stability, we next sought to assess the capability of mRPZ for in vivo mRNA delivery. Building on these findings, we investigated whether the advantages observed in vitro could translate into functional, biocompatible mRNA delivery within a physiological environment.

### 3.4. In Vivo Application

**Figure 10.**
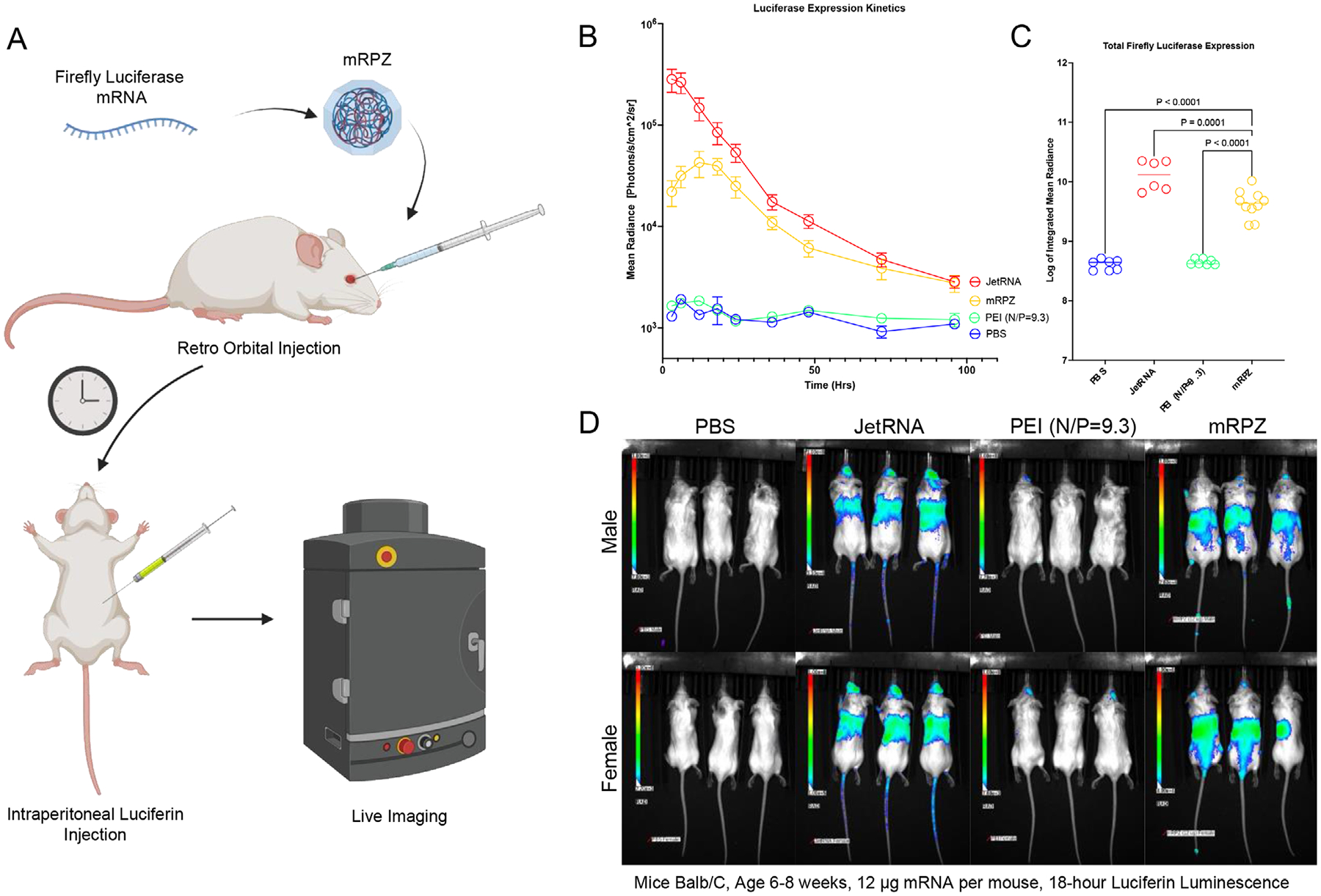
Firefly luciferase-expressing mRNA was encapsulated using our encapsulation for mRPZ and delivered to Balb/C mice intravenously. Luciferase expression was measured by luminescence resulting from intraperitoneal luciferin injection A). The luciferase expression kinetics were measured over 96 h (presented as mean ± StdEM) B) and the integrated luciferase activity, which represents total luciferase expression was calculated (presented as the median) C). Live images of the luciferase activity were taken at 18 h, in the maximum mRPZ expression time window D). Sample sizes are implied by points, and statistical significance (P) was assessed using one-way ANOVA.

After successful in vitro testing, we scaled and translated mRPZ into an in vivo gene delivery model using Balb/C mice. Instead of eGFP expressing mRNA, utilizing the same synthetic encapsulation process, we encapsulated 12 μg of firefly luciferase mRNA (1920 nt). Firefly luciferase generates bioluminescence in the presence of luciferin, which can be injected into mice to monitor luciferase expression (Figure 10A). The work performed here demonstrates one of the first applications of mRNA delivery using a MOF platform in vivo.

Balb/C mice were injected intravenously through the retro-orbital vein with mRPZ (N = 10), mRNA-PEI utilizing an N/P ratio of 9.3 (PEI N/P = 9.3) (N = 7), in vivo *JetRNA*+ (JetRNA) a commercial lipid reagent (N = 6), or PBS (N = 7). The expression of luciferase was monitored over 96 h by injecting mice in their intraperitoneal cavity with a luciferin DPBS solution and measuring the luminescence with an IVIS live imaging system. The images taken were analyzed using IVIS software and the mean radiance was collected measuring the entire body of each mouse. The kinetics of luciferase expression were tracked using the image data over 96 h (Figure 10B). mRPZ shows the same clear delayed maximum expression of mRNA in vivo as it does in vitro when compared to a commercial lipid reagent. mRPZ reaches a maximum expression between 12 to 18 h post-injection, while JetRNA reaches a maximum of 3 h post-injection, both gradually taper off over 96 h. PEI N/P = 9.3 showed some expression in vivo but not statistically above background (PBS), even when controlling for sex (Figure S40A, Supporting Information).

Trapezoidal integration approximation was used to find the area under the curve for each mouse, and the log of the integrated mean radiance, which represents the total luciferase expression was used to compare each group’s luciferase expression (Figure 10C). Notably, mRPZ, which contains the same amount of PEI, significantly outperforms PEI N/P = 9.3 over ten-fold. However, JetRNA significantly outperforms mRPZ by about three-fold in total expression. PEI N/P = 9.3 did express luciferase, as seen in Figure 10D, but luciferase expression was not statistically different from the background of the PBS mice. This holds even when controlling for sex (Figure S40B, Supporting Information). When controlling for sex, both mRPZ and JetRNA demonstrate significantly more expression in female mice compared to male mice. Smaller doses of mRPZ, 6 μg per mouse, interestingly yielded total luciferase expression levels that were not statistically different from the 12-μg dose (Figure S41, Supporting Information).

**Figure 11.**
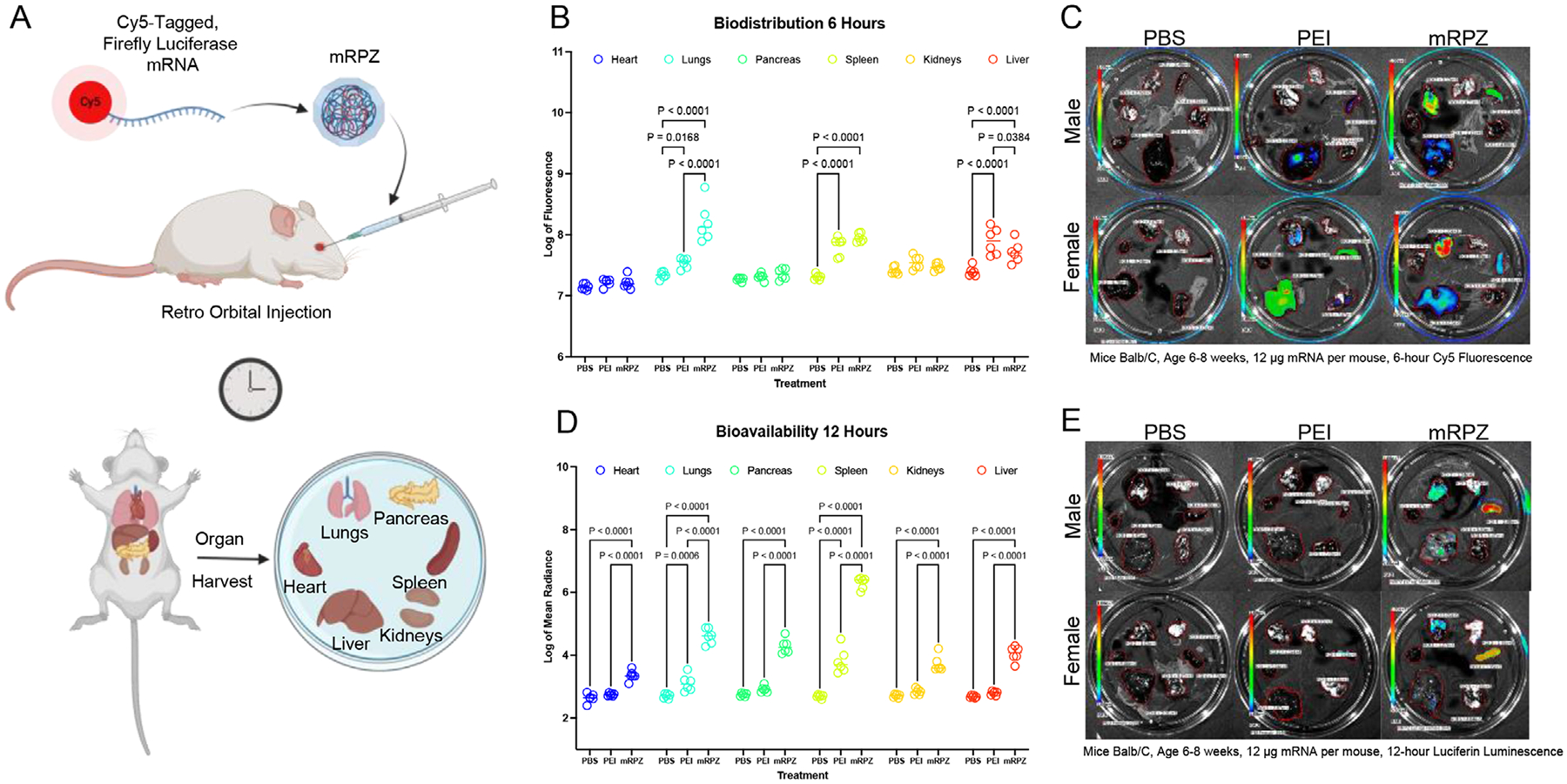
Cy5 tagged mRNA expressing luciferase was delivered to Balb/C mice, and after 6 h the mice organs were collected and imaged A). The biodistribution of the mRNA, and hence particles, was quantified B) and representative images are shown C). The bioavailability of the luciferase mRNA was quantified after 12 h D), and representative images are shown E). Data are presented as the median, sample size is N = 6 for all samples, and statistical significance (P) was assessed with two-way ANOVA.

The biodistribution of mRPZ (N = 6), compared to PEI N/P = 9.3 (N = 6) and PBS (N = 6), was determined by injecting particles encapsulating Cy5-tagged mRNA expressing luciferase into mice, waiting 6 h, then collecting their organs (heart, lungs, pancreas, spleen, kidneys, and liver) and imaging them for Cy5 fluorescence. Biodistribution, as determined by Cy5 localization, represents the location of the mRNA, and hence where the injected particle traffics in mice (Figure 11A). Our findings show the mRPZ particle traffics primarily to the lungs, spleen, and liver in the first 6 h when comparing the log of the Cy5 fluorescence against the PBS control (Figure 11B,C). This distribution pattern is consistent with the distribution of PEI N/P = 9.3. Interestingly, we observed mRPZ particles traffic significantly more to the lungs and significantly less to the liver compared to PEI N/P = 9.3 particles. This result is consistent across sex for the lungs, but not for the spleen and liver. When comparing the spleen, PEI N/P = 9.3 particles trafficked significantly less to the spleen than mRPZ in male mice while female mice showed no significant difference. On the other hand, PEI N/P = 9.3 particles trafficked significantly more to the liver compared to mRPZ in female mice, while male mice showed no statistical difference (Figure S42, Supporting Information).

The bioavailability represents the location in which the mRNA preferentially expresses luciferase compared to PEI N/P = 9.3 and PBS. Briefly, mRNA expressing firefly luciferase was encapsulated in mRPZ or delivered with PEI to Balb/c mice intravenously, after 12 h, mice were injected with luciferin and sacrificed, their organs harvested, and imaged (Figure 11D,E). The luminescence of the organs was measured with the IVIS imaging system, and the log mean radiance was compared. In comparison to PEI N/P = 9.3, mRPZ resulted in significantly higher expression in all collected organs, well above PBS background. These findings are consistent across sex (Figure S43, Supporting Information). PEI N/P = 9.3 mRNA was only expressed above background in the lungs and spleen.

**Figure 12.**
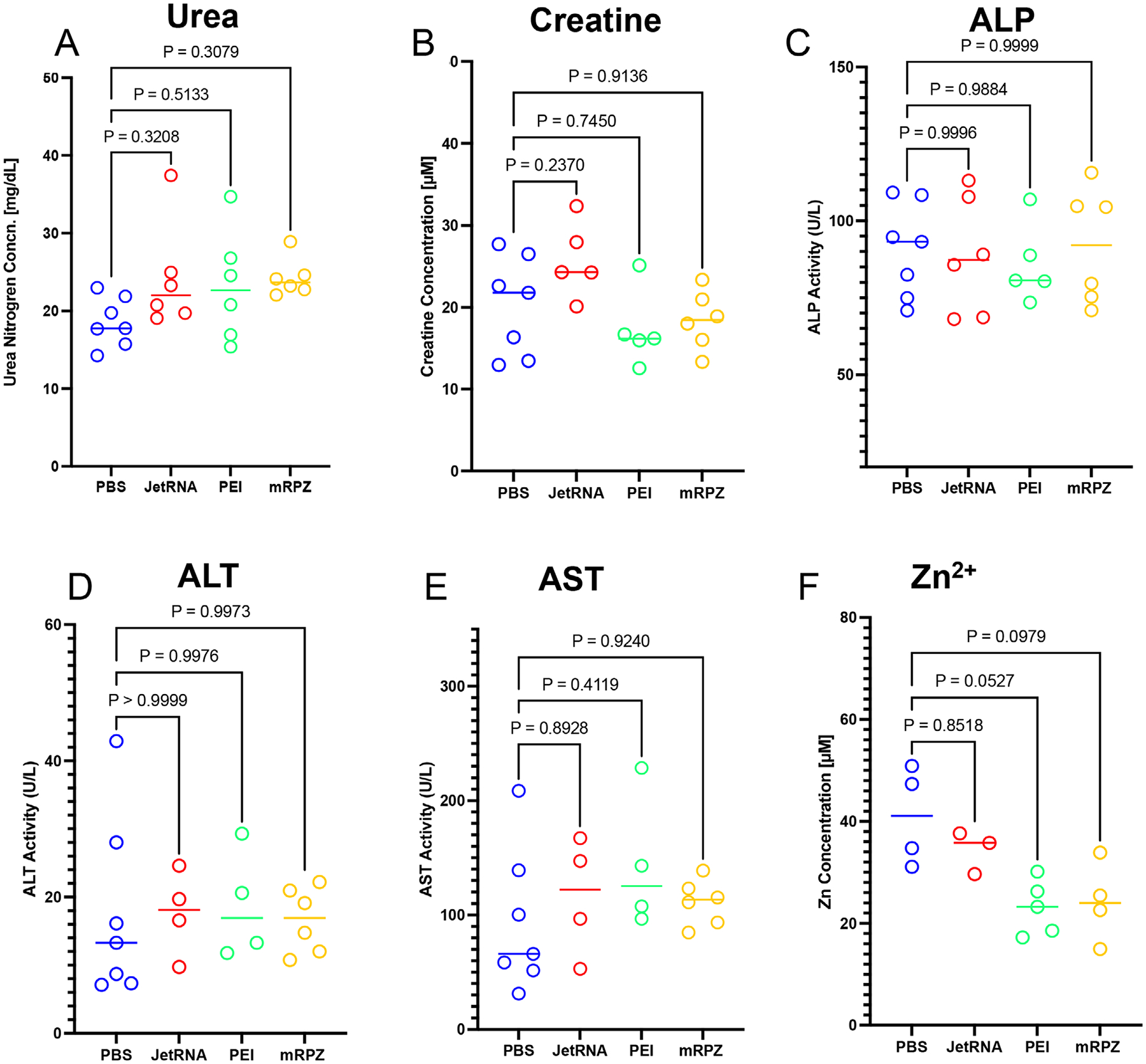
Mouse health was assessed by measuring Urea concentration A), Creatine concentration B), ALP activity C), ALT activity D), AST activity E), and Zinc concentration F). Data presented as median and statistical significance (P) was assessed with one-way ANVOA.

Toxicity of the particles was assessed using the collected serum from the sacrificed mice 7 days after injection, measuring the Urea, Creatine, and Zinc (Zn^2+^) concentrations as well as the Alkaline phosphatase (ALP), alanine transaminase (ALT), and aspartate transaminase activity (AST) (Figure 12). These serum factors are a measure of mouse health. Based on our observations, mRPZ (N = 6), PEI N/P = 9.3 (N = 6), and JetRNA (N = 6) caused no statistical difference in the serum factors measured compared to a healthy control (PBS). Samples processed incorrectly were removed from assay data set. Of particular interest was the serum zinc concentration, as mRPZ would theoretically release zinc ions upon degradation of the ZIF-8 Coating. Not only was the zinc concentration of mRPZ lower than the healthy control, but it was also equivalent to PEI N/P = 9.3 which contains no zinc. H&E (Hematoxylin and Eosin)-stained sections of major organs (liver, kidney, spleen, lung, and heart) collected one-week post-injection revealed no observable tissue damage, inflammatory infiltration, or architectural disruption, indicating good biocompatibility of the mRPZ formulation (Figure S44, Supporting Information). Overall, the mice showed minimal signs of toxicity when compared to a healthy control and are within healthy standards. It is important to note that mice being injected intravenously were very sensitive to the concentration and degree of resuspension of the mRPZ particles. pH changes caused by ZIF-8 were ruled out as a factor contributing to sensitivity, as buffered solutions maintained a stable pH upon the addition of mRPZ (Figure S45, Supporting Information). Some mice were visibly lethargic after injection but recovered quickly. We note that high concentrations of the mRPZ caused the death of 4 out of 6 mice at a 24-μg dose and mRPZ particles that were poorly resuspended caused the death of 2 mice at a 12-μg dose. The colloidal stability of the particles stands to be improved.

**Figure 13.**
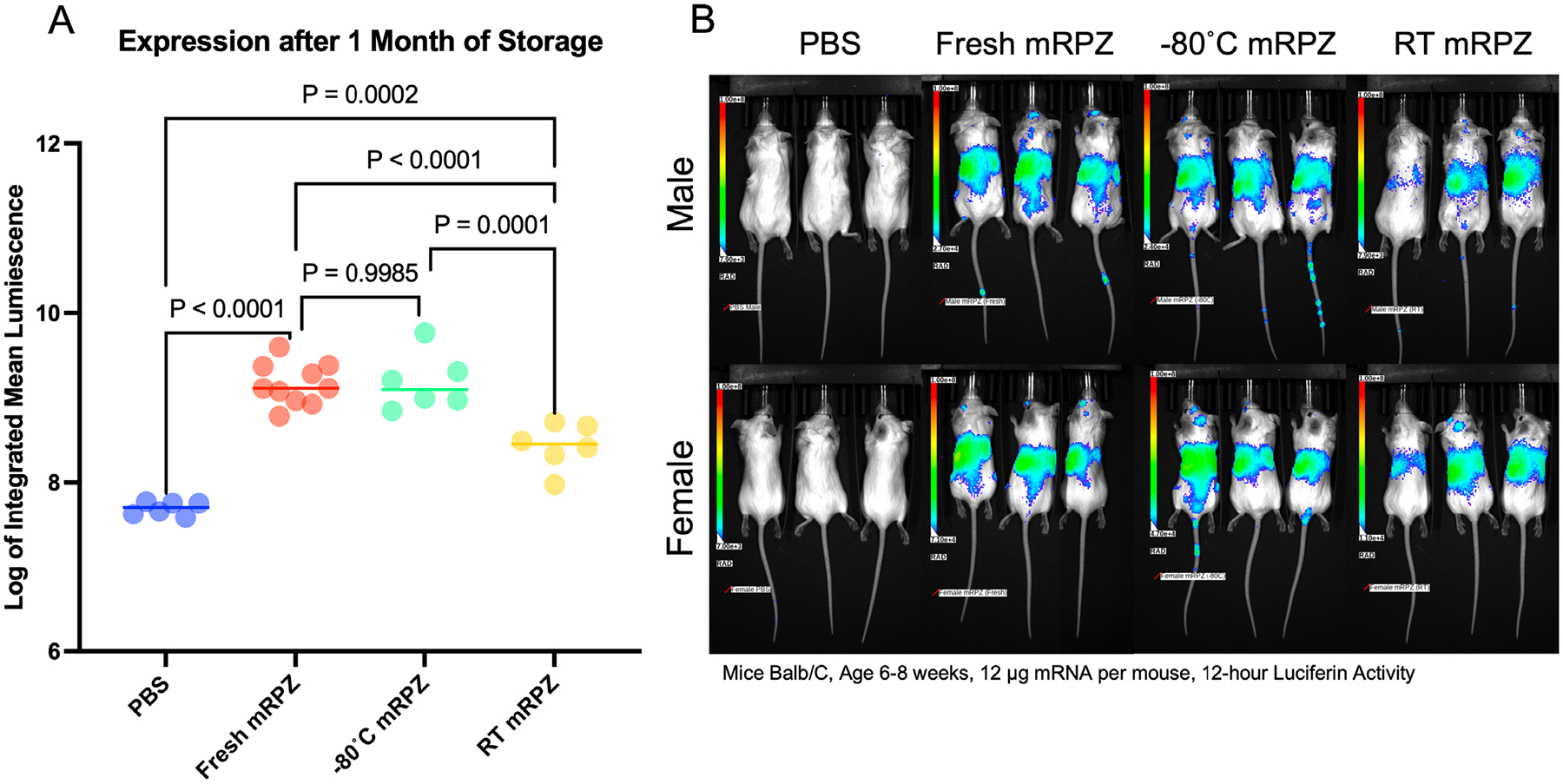
mRPZ stored at −80 °C and RT stored under vacuum were injected in mice and the total luciferase expression was measured A). Representative images of the mice (N = 6, per group) are shown B). Data presented as the median and statistical significance (P) assessed with one-way ANOVA.

The long-term stability of the particles was assessed at room temperature for one month, given the success of tested particles in vitro. Particles were synthesized and stored at room temperature under vacuum and injected intravenously in mice. The resulting luciferase activity was measured between 12 and 24 h, capturing the maximum expression time range identified in Figure 10B. The integrated mean radiance was used to find the luciferase expression at that time point and compared to mRPZ stored at −80 °C for a month and mRPZ freshly synthesized. A PBS control was included to determine background radiance (Figure 13). As expected freshly synthesized mRPZ performed equally to mRPZ stored at −80 °C for one month. mRPZ stored at room temperature produced luciferase expression that was significantly less than −80 °C stored mRPZ, but still significantly above background (PBS) over five-fold.

The successful translation of mRPZ from in vitro systems to an in vivo murine model represents a key milestone in the application of MOFs for mRNA delivery. Our results demonstrate, for the first time to our knowledge, that a MOF-based system can effectively encapsulate and deliver functional mRNA in vivo, resulting in quantifiable and sustained protein expression. When compared to both a PEI-based formulation (PEI N/P = 9.3) and a commercial lipid reagent (JetRNA), mRPZ displayed a distinct expression profile characterized by a delayed but sustained peak of luciferase expression, with maximum activity observed between 12–18 h post-injection. This delayed expression mirrors in vitro trends and may reflect the unique release kinetics conferred by the MOF matrix.

Despite containing an equivalent PEI content, mRPZ achieved over an order-of-magnitude greater cumulative expression than PEI N/P = 9.3, underscoring the critical role of the MOF shell in enhancing delivery efficiency and protecting cargo. While JetRNA induced a higher total expression (≈3-fold greater than mRPZ), the difference must be contextualized within the physicochemical and storage advantages mRPZ provides, including enhanced stability and potential for dry-state storage at ambient temperatures.

Biodistribution analysis further revealed that mRPZ traffics primarily to the lungs, spleen, and liver, with significantly enhanced pulmonary accumulation relative to PEI N/P = 9.3. These findings are noteworthy as they suggest potential utility for pulmonary-targeted gene therapies. Moreover, mRPZ-mediated delivery yielded significantly higher mRNA bioavailability across all major organs tested, compared to PEI, reinforcing the platform’s versatility for systemic delivery. Interestingly, organ-level expression and biodistribution displayed sex-dependent differences, which may warrant further investigation into hormonal or physiological contributors to nanoparticle trafficking and uptake.

Toxicity assessments revealed no significant deviations in liver (AST, ALT, and ALP levels) and kidney function markers (Urea and Creatine levels) or serum zinc levels relative to healthy controls. The absence of histological abnormalities further supports the biocompatibility of mRPZ. However, dose-dependent lethality observed at higher particle concentrations and with poorly resuspended formulations highlights a need for optimization in colloidal stability and formulation handling—a known limitation of ZIF-8-based systems.

Importantly, we demonstrate that mRPZ retains functional performance after one month of storage at room temperature under vacuum, with preserved luciferase expression well above background. This thermal stability may address critical barriers to RNA therapeutic distribution and storage, particularly in resource-limited settings where cold-chain logistics remain challenging.

In summary, mRPZ represents a promising alternative to lipid-based carriers for mRNA delivery, offering distinct advantages in stability, tunability, and biodistribution. While further optimization is required to enhance total expression levels and improve formulation stability, these in vivo results establish MOF-based nanocarriers as a viable and expandable platform for nucleic acid therapeutics. Our findings lay the groundwork for future exploration into targeted delivery, endosomal escape mechanisms, and therapeutic efficacy in disease models.

## 4. Conclusion

This study highlights ZIF-8′s potential as an effective mRNA delivery platform, building upon its established role in biomedical applications. Our work demonstrates the ethanol-based coprecipitation of mRNA and ZIF-8 which achieves both stability during synthesis and efficient loading of mRNA. Addressing concerns of mRNA leakage from ZIF-8, we introduced PEI into the synthesis process, enhancing mRNA stability and protection against nucleases and biological media exposure. Characterization analyses confirmed the successful integration of mRNA-PEI within ZIF-8, establishing the resultant mRPZ as a robust delivery system with high loading efficiency and capacity. In vitro experiments demonstrated mRPZ’s superior performance over conventional mRNA-PEI complexes, showcasing enhanced cellular uptake, minimal toxicity, and significant protein expression across diverse cell lines. Notably, mRPZ exhibited delayed but strong and sustained expression kinetics, indicative of prolonged mRNA release. Our in vivo work confirms our findings in vitro are translatable and the interesting properties demonstrated in vitro, like delayed release and improved expression compared to PEI alone, are consistent. We also noted interesting particle trafficking as the ZIF-8 coating increased particle uptake and expression in the lungs.

Furthermore, our study demonstrates the long-term stability of mRNA-PEI encapsulated within mRPZ at varying temperatures, which may be advantageous for mRNA vaccine storage and transportation. While ZIF-8 coating improves the stability of the mRNA-PEI complex, mRNA expressibility still decreases with storage time. Overall, our findings present a ZIF-8-based delivery system as a promising candidate for mRNA therapeutics, particularly vaccines, offering enhanced stability, efficient delivery, and prolonged-expression kinetics. Future work exploring immune responses and improving the colloidal stability of mRPZ is needed.

## 5. Experimental Section

### Materials and Reagents:

Zinc nitrate hexahydrate (Alfa Aesar, Haver-hill MA) and 2-methylimidazole (Sigma Aldrich, St. Louis MO) were used for ZIF-8 precursor solutions. Ethanol 200 Proof (Pharmco, Toronto Ontario), methanol (Sigma Aldrich, St. Louis MO), and nuclease-free water (Invitrogen, Waltham MA) were used as solvents. Linear 20 kDA polyethyleneimine, Linear 10 kDA PEI, and branched 25 kDA PEI were purchased by Sigma Aldrich (St. Louis, MO). Linear 40 kDa PEI was purchased from Polysciences (Warrington, PA). F12/DMEM cell culture media, Antibiotic-Antimycotic 100x, 0.05% Trypsin, and Opti-MEM were purchased from Gibco (Grand Island, NY). DMEM without phenol red and Fetal Bovine Serum were purchased from Cytiva (Marlborough, MA) and VWR (Radnor, PA), respectively. LIVE/DEAD^™^ Fixable Blue, 10x Tris-Borate Buffer, Lipofectamine 2000, Lipofectamine Messenger Max, and SyberSafe were purchased from Invitrogen (Waltham, MA). Triton X-100, DAPI, and Heparin Sulfate were purchased from Sigma Aldrich (St. Louis, MO). RNase A, RNA Loading dye, and ssRNA ladder were purchased from New England Biolab (Ipswich, MA). Quantifluor RNA Assay kit was purchased from Promega (Madison, WI). Paraformaldehyde was purchased from Thermo Scientific (Waltham, MA). Agarose was purchased from Fisher Scientific (Pittsburgh, PA). Ethylenediaminetetraacetic acid was purchased from Ambeed (Arlington, IL). eGFP-mRNA (5moU) and Firefly Luciferase-mRNA (5moU) were purchased from Tri-Link (San Diego CA), EZ CAP Cy5-eGFP-mRNA (5 mo UTP) and EZ Cap Cy5 Firefly Luciferase mRNA (5-moUTP) were purchased from APExBIO (Huston, TX) and eGFP-pDNA (13 031 pcDNA3-EGFP) was purchased from Addgene (Watertown, MA). D-Luciferin was purchased from Gold Biotechnology (St. Louis, MO). In vivo JetRNA+ controls were purchased from Poly-plus (Illkirch-Graffenstaden, France). Blood assays for creatine (ECRT-100), alkaline phosphatase (QFAP-100), aspartate transaminase (EASTR-100), alanine transaminase (EALT-100), and zinc concentration (DIZN-250) were obtained from BioAssay Systems (Hayward, CA). A Urea Nitrogen (Bun) assay detection kit was obtained from Arbor Assays (Ann Arbor, MI).

### Preparation of ZIF-8:

Solutions of zinc nitrate hexahydrate and 2-methylimidazole were prepared separately by dissolving 0.3 grams and 0.66 grams, respectively, in 14.3 mL methanol. The two solutions were mixed and stirred vigorously for 60 min. The particles were isolated by centrifugation at 10000 × g for 10 min. Particles were washed with methanol two times. The resultant ZIF-8 particles were vacuum dried at 75 °C overnight.

### Preparation of mRNA@ZIF-8:

Precursor solutions of zinc nitrate hexahydrate (11.9 mg mL^−1^) and 2-methylimidazole (13.15 mg mL^−1^) were prepared in methanol, ethanol, or nuclease-free water. mRNA (1 mg mL^−1^), 1 μg, was aliquoted into a centrifuge tube and mixed with 10 μL of 2-methyl imidazole solution. Another 10 μL of zinc nitrate was added and mixed for 30 s. The solution was incubated at room temperature for 2 h. Particles were isolated by centrifugation at 16000 × g for 5 min and washed with ethanol two times. Particles were stored at −80 °C until use.

### Preparation of mRNA-PEI@ZIF-8:

Precursor solutions of zinc nitrate hexahydrate (11.9 mg mL^−1^) and 2-methylimidazole (13.15 mg mL^−1^) were prepared in ethanol. mRNA (1 mg mL^−1^), 1 μg, was aliquoted into a centrifuge tube and mixed with 10 μL of 2-methyl imidazole solution. After thoroughly mixing the mRNA, 1 μL of Linear 20 kDA polyethyleneimine (1–2 mg mL^−1^) was added and mixed thoroughly. This solution was allowed to incubate for 15 min. After incubation, 10 μL of zinc nitrate was added and mixed for 30 s. The solution was incubated at room temperature for 2 h. Particles were isolated by centrifugation at 16000 × g for 5 min and washed with ethanol two times. Particles were stored at −80 °C until use. Procedure had been successfully scaled using 24 μg of mRNA maintaining the same weight ratios and precursor volumes.

### Gel Electrophoresis:

Equal volumes of samples were mixed with RNA loading dye. Samples containing PEI were additionally treated with 4 μL of heparin (25 mg mL^−1^). Samples were heated at 60 °C for 10 min. A native 1% agarose gel was prepared in 1x Tris-Borate EDTA Buffer with a 1:10000 dilution of SyberSafe. Samples were loaded into the gel and a potential of 80 V was applied for 45 min. Gels were imaged using a BioRad ChemiDock imaging system.

### RNA Loading Efficiency:

Samples were run using gel electrophoresis, as described above. The resulting image provided by the ChemiDock imaging system was analyzed using ImageJ software. The RNA band intensity, which was correlated with the amount of RNA, was quantified with ImageJ. A standard curve was used to determine the amount of RNA loaded. Loading efficiency was also determined using Promega Quantifluor assay. Samples were exfoliated with 100 mM EDTA (pH = 4.5) and treated with 100 μg of heparin. Samples were heated at 60 °C for 10 min. Quantifluor assay solution was prepared per the provided protocol. Samples (20 μL) were added to the assay solution and evaluated with a plate reader (SpectraMax i3) with 492 nm excitation and 540 nm emission.

### mRNA Leakage:

One microgram of Cy5-labeled mRNA was suspended in 100 μL PBS with 10% FBS and incubated at 37 °C on a hula shaker. At defined intervals (0–24 h), samples were centrifuged at 18000 × g for 5 min to separate released mRNA from nanoparticles. The supernatant was collected and stored at −80 °C, and the pellet was resuspended in fresh PBS with 10% FBS for continued incubation. After 24 h, all supernatants were thawed and Cy5 fluorescence was measured using a SpectraMax i3 plate reader (*λ*_ex_ = 650 nm, *λ*_em_ = 670 nm) to quantify mRNA release over time.

### RNase Treatment:

Samples (≈0.5 μg RNA) were mixed with 0.85 μg of RNase A (NEB) diluted in PBS and incubated at 37 °C for 2 h. Samples were centrifuged at 16000 × g for 5 min and the supernatant was removed and then washed with 1x PBS. One sample at a time, 10 μL of RNA loading dye was added to each MOF-containing sample then 10 μL of 100 mM EDTA was immediately added with 4 μL of heparin sulfate (25 mg mL^−1^). Samples were heated to 60 °C for 10 min. Samples were run on a native, Sybersafe, 1% agarose gel at 80 V for 40 min. The gel image was obtained using a Bio-Rad ChemiDock imager.

### Scanning Electron Microscopy and EDS:

Samples were suspended in ethanol, drop cast onto a silicon wafer, and mounted on an SEM stub using carbon tape. Samples were allowed to dry at room temperature. The samples were sputter-coated with 2 nm of platinum. Sample images were taken at 30 kV and a working distance of 10 mm or less on a Tescan Mira3 (Materials Characterization Facility, Carnegie Mellon University). Energy dispersive spectroscopy was performed at 10 kV, 3.2 nA current, and a working distance of 12 mm using a Thermofisher FEI Apreo equipped with APEX EDS (Nanoscale Fabrication and Characterization Facility, University of Pittsburgh).

### Transmission Electron Microscopy, Scanning Electron Microscopy, and EDS:

Samples suspended in ethanol were drop-cast onto a mesh carbon support film with copper and dried. TEM sample images were taken using an FEI Tecnai F20 TEM/STEM or Thermo Fisher Themis 200 G3 Aberration Correction (Materials Characterization Facility, Carnegie Mellon University) STEM at 80 kV with a spot size of 6. EDS/STEM was performed using the Thermo Fisher Themis with Super-X EDS detector at 200 kV and spot size 6. Element K-lines were used.

### Fourier Transform Infrared (FTIR) Spectroscopy:

FTIR was used to confirm the formation of MOF nanoparticles and determine functional groups of final components using a Perkin Elmer Frontier vibrational spectrophotometer (Spectrum 100, PerkinElmer Inc., USA). The particles were first lyophilized and then measured at room temperature in a wave number range of 4000–600 cm^−1^ with a resolution of 2 cm^−1^ against the KBr background.

### Dynamic Light Scattering and Nanoparticle Tracking Analysis:

Samples were resuspended in 1 mL of ethanol and sonicated. The zeta potential and particle size were determined using a Malvern Zetasizer. NTA Samples were suspended in 10 μL ethanol and diluted in PBS and run on a ZetaView instrument by Particle Metrix.

### Powder X-Ray Diffraction:

Samples were suspended in ethanol and drop-cast onto a silicon wafer. Samples were allowed to dry at room temperature. The crystalline structure of the particles was obtained using Malvern Panalytical Empyrean XRD (Materials Characterization Facility, Carnegie Mellon University) with Cu anode K-alpha 1 = 1.540 at 45 mV and 40 mA.

### Thermogravimetric Analysis:

Samples were resuspended in 100 μL of ethanol and sonicated to ensure most of the sample was removed from the tube. The suspended sample was added to a crucible and run using a Perkin Elmer TGA 4000 under a Nitrogen atmosphere. Samples were heated to 50 °C and held at 50 °C for 30 min to remove solvent. The sample was then ramp scanned from 50 °C to 800 °C at 10 °C min^−1^.

### RNA Molecular Probing:

Quantifluor dye was diluted in 1x PBS 1:400 and 180 μL were aliquoted on a 96-well plate. Samples were resuspended in 5 μL ethanol and sonicated. Samples were treated accordingly: EDTA treated: 5 μL of 100 mM EDTA + 95 μL PBS, Heparin treated: 5 μL of heparin (25 mg mL^−1^) + 95 μL PBS, and Dual treated: 5 μL of EDTA + 5 μL heparin + 90 μL PBS. Any samples treated with heparin were heated at 60 °C for 10 min. After EDTA/heparin treatment, 20 μL were collected as the suspension samples, then the rest of the sample was centrifuged at 16100 × g for 5 min. 20 μL of the supernatant was collected. Twenty microliters of the sample was added to a well with 180 μL of Quantifluor dye solution, mixed well, and incubated on a shaker for 5 min. Resultant fluorescence was evaluated with a plate reader (SpectraMax i3) using 492 nm excitation and 540 nm emission.

### Cell Culture:

Human embryonic kidney (HEK-293t) cells and Chinese Hamster Ovary (CHO) cells were provided graciously by the Ren Lab at Carnegie Mellon University. Human Cervical Cancer (HeLa) cells were graciously provided by the Wayne Lab at Carnegie Mellon University. A549, MDA-MB-231, and RAW264.7 cells were obtained from the American Type Culture Collection (ATCC). All cell lines, except CHO, were cultured with DMEM supplemented with 10% FBS and Antibiotic-Antimycotic. CHO cells were cultured in F12/DMEM (1:1) supplemented with 10% FBS and Antibiotic-Antimycotic. The cells were kept in a 100% humified atmosphere containing 5% CO_2_ at 37 °C. ATCC protocols were followed for culturing and handling.

### Transfection:

The particles were resuspended in 10 μL of ethanol and sonicated for 5 min then resuspended in Opti-MEM to a final concentration of 10 ng mRNA μL^−1^. The particles were added to cells in a 96-well plate using 100 ng per well. Lipofectamine 2000 and Messenger Max were prepared per the manufacturer’s protocol. mRNA-PEI complexes were prepared by suspending mRNA and PEI separately in Opti-MEM then mixing both solutions and incubating for 15 min. Cells were incubated with particles for 48 h, fixed using 4% paraformaldehyde in PBS, permeabilized with a 0.1% triton X-100 solution, and stained with DAPI. A lipofectamine-positive control was used. The fluorescence intensity was measured using a SpectraMax i3 plate reader at 475/515 nm for eGFP and 350/460 nm for DAPI. Imaging was performed using a Thermofisher EVOS M7000 with a DAPI, Cy5, and GFP filter cube. Due to Cy5 interrupting the expression of eGFP, Cy5-tagged eGFP mRNA was mixed with untagged eGFP mRNA in a 1:10 ratio.

### Flow Cytometry:

Two days post-transfection, media was removed. Cells were washed with PBS and then incubated in trypsin for 20–30 min. After incubation, PBS was added to each well, and cells were gently resuspended. Cells were moved to a 96-well u-bottom plate and centrifuged at 300 × g for 5 min. The supernatant was removed, and the pellet was resuspended in PBS containing a 1:10000 dilution of Live/Dead Fixable Blue and incubated for 15 min on ice in the dark. The plate was centrifuged again at 300 × g for 5 min and the staining buffer was removed. Cells were fixed with 4% paraformaldehyde in PBS on ice for 10 min. The cells were centrifuged at 300 × g for 5 min and washed 2x with PBS. Cells were resuspended in flow buffer and run on a NovoCyte Flow Cytometer.

### Cell Metabolic Activity:

HEK293t cells were plated at a seed density of 30000 cells per well in a black-walled, clear bottom 96-well plate, phenolred free media was used to reduce background, and one column of wells was left without cells. Resazurin was prepared for a final working concentration of 44 μm. Cells were treated with mRPZ, ZIF-8, Linear 20 kDa PEI, and lipid particles and allowed to incubate for 4–6 h. One column of wells was treated with 70% ethanol to kill all the cells. After incubation, Resazurin solution was added to the well, and the fluorescence (excitation 560 nm, emission 590 nm) was measured between 4–24 h after adding resazurin on the SpectraMax i3 plate reader.

### Time to Expression Assay:

The particles were resuspended in 10 μL of ethanol and sonicated for 5 min then resuspended in Opti-MEM to a final concentration of 10 ng mRNA μL^−1^. The particles were added to cells in a 96-well plate using 100 ng per well. Lipofectamine 2000 and Messenger Max were prepared per the manufacturer’s protocol. RNA-PEI complexes were prepared by suspending RNA and PEI separately in Opti-MEM then mixing both solutions and incubating for 15 min. Cells were maintained in phenol red-free media with 10% FBS. Fluorescence was measured at different time points using a SpectraMax i3 plate reader at 475/515 nm.

### Thermal Storage:

Samples were prepared as noted above. Samples were dried using a DNA drier at room temperature for 1 h and stored closed under a vacuum in descant. After a set amount of time, samples were resuspended and used to transfect cells. RNA and exfoliated (Ex) RNA from ZIF-8 were transfected using lipofectamine Messenger Max. Lipofectamine 2000 and Messenger Max were prepared with RNA stored at −80 °C as a control. Gel electrophoresis was performed on stored samples according to the previously mentioned protocol.

### mRPZ Induced pH Change:

Twelve micrograms of mRNA formulated in mRPZ nanoparticles was added per sample per well. Samples were incubated at 37 °C in 100 μL of different buffer solutions nuclease-free water, 1× PBS, serum-free media, and media supplemented with 10% fetal bovine serum (FBS). The pH was measured using pH paper.

### In Vivo mRNA Delivery and Efficiency:

All experiments with animals were approved by the Institutional Animal Care and Use Committee (IACUC) at Carnegie Mellon University under the protocol number IPROTO202400000004. Six- to 8-week-old Balb/c Mice (Male/Female) (000651) were purchased from Charles River and allowed to acclimate to vivarium conditions for at least 3 days before experiments were performed. Mice were anesthetized and injected in the retro-orbital vein with mRPZ particles generated with Firefly Luciferase expressing mRNA. Before injection, particles were suspended in 5 μL of 200 proof ethanol and sonicated, then DPBS was added stepwise, with repeated sonication, until a final volume of 170 μL was achieved. The PEI control was prepared by complexing mRNA and linear 20 kDA PEI in a 5% sterile glucose solution for 15 min followed by retro-orbital injections. In vivo, JetRNA+ was prepared per the manufacturer’s protocol. Luciferase expression kinetics were collected starting at 3 h post-injection. Mice were injected with 110 μL of D-Luciferin (30 mg mL^−1^ in DPBS) and luminescence was measured 10 to 15 min post-injection using IVIS (PerkinElmer, Waltham, MA). Particle bioavailability and biodistribution were assessed by sacrificing the mice after 12 and 6 h, respectively, and harvesting the heart, lungs, spleen, pancreas, kidneys, and liver. The organs’ fluorescence and luminescence were measured using the IVIS^®^ system. Major organs (heart, lungs, live, kidney, and spleen) were harvested, embedded in paraffin, sliced, and stained with H&E. Blood was collected from mice, then serum was isolated and assessed for nitrogen urea, creatine, alkaline phosphatase, aspartate transaminase, alanine transaminase, and zinc concentration. Blood samples were diluted following manufacturers protocol. If samples with multiple dilutions did not produce a value within the detection range of the protocol, they were removed from the data as they were not processed properly. Note particles generated using 24 μg of mRNA were unexpectedly hazardous to mice at the injected concentration with 2 of 6 mice surviving. Additionally, particles that were poorly resuspended caused the death of 2 mice at 12 μg doses.

### Graphing and Statistical Analysis:

All data were pre-processed by assessing for outliers using the ROUT method (Q = 2%) and tested for normality using the Shapiro–Wilk test. Where appropriate, data were log-transformed to correct for non-normal distributions. Results were presented as median or mean ± StdEM or SD as specified in figure legends. Sample sizes (N) are indicated in each figure or figure legend and refer to either biological replicates (i.e., individual mice or cell passages) or technical replicates. Statistical significance between groups was assessed using one-way or two-way ANOVA, as appropriate, with Browne, Forsythe and Welch and parametric tests run as indicated in the figure legend. A significance threshold of *α* = 0.05 was used for all tests. All statistical analyses were performed using GraphPad Prism v10.

## Supplementary Material

Supplemental Figures

Supporting Information is available from the Wiley Online Library or from the author.

## Figures and Tables

**Table 1.** Brief literature review of MOF-based nucleic acid delivery systems.

Long to Short	Nucleic Acid	Size	Therapeutic Application	MOF	Test System	Refs.
	siRNA	19–21 nt	Multidrug-Resistant Ovarian Cancer	UiO-Cis	Human Ovarian Cancer (SKOV-3)	[28]
	siRNA	22–23 nt	Multidrug-Resistant Breast Cancer	MIL-101	Human Breast Cancer (MCF-7/T)	[29]
	siRNA/miRNA	19–23 nt	Colorectal Cancer	MIL-100 MIL-101_NH_2_	Human Colorectal Cancer (SW480)	[30]
	miRNA	21–22 nt	Chemodynamic Therapy	ZIF-8	Breast Cancer (MDA-MB-231) Tumor Bearing BALB/C Mice	[35]
	miRNA	40 nt	Ischemic Stroke Therapy	Ca-MOF	Neuronal Stem Cells Mice	[36]
	ssDNA	11–53 nt	Immune Cell Transfection	Ni-IRMOF-74	Mouse Primary Immune (CD4+T, B Cells), Macrophage (RAW 264.7, THP-1)	[31]
	sgRNA	65 nt	CRISPR/Cas9 Genome Editing	ZIF-8	Chinese Hamster Ovary (CHO)	[37]
	mRNA	>1900 nt*	Proof of Concept (Luciferase)	PGMA(EA)-UiO-66	Glioblastoma (U-87) Human Umbilical Vein (HUVEC)	[34]
	pDNA	6549 bp	Proof of Concept (eGFP)	ZIF-8	Human Prostate Cancer (PC-3)	[8]
	pDNA	6549 bp	Proof of Concept (eGFP)	ZIF-8	Human Islet-Derived Progenitor Cells	[32]
	pDNA	≈4700 bp	Proof of Concept (eGFP)	ZIF-8	Human Breast Cancer (MCF-7)	[16]
	pDNA	≈9200 bp	Prostate Cancer	ZIF-8	Human Prostate Cancer (PC-3)	[33]
	pDNA	5448 and 9288 bp	CRISPR/Cas9 Genome Editing	ZIF-8	Sarcoma (U-2 OS)	[38]
	pDNA	10243 bp	Sperm Sperm-Mediated Gene Transfer	ZIF-8	Spermatozoa	[15]

* Not officially reported. *Abbreviations*: short interfering RNA (siRNA), microRNA (miRNA), short guide RNA (sgRNA), single-stranded DNA (ssDNA), messenger RNA (mRNA), and plasmid DNA (pDNA).

## Data Availability

The data that support the findings of this study are available from the corresponding author upon reasonable request.
