# Supplementary material for: Synthetic Strategy for mRNA Encapsulation and Gene Delivery with Nanoscale Metal-Organic Frameworks
Source: Adv Funct Mater. Author manuscript; Available in PMC 2026 May 23. (PMC13196661; doi:10.1002/adfm.202504465)
Supplement: Supplemental Figures [file NIHMS2176620-supplement-Supplemental_Figures.pdf]

ADVANCED FUNCTIONAL MATERIALS

Supporting Information

for *Adv. Funct. Mater.*, DOI 10.1002/adfm.202504465

Synthetic Strategy for mRNA Encapsulation and Gene Delivery with Nanoscale Metal-Organic Frameworks

Harrison Douglas Lawson, Huy Hoang Nguyen, Keng-Jung Lee, Nattarat Wongsuwan, Ayesha Tupe, Mengrou Lu, Mariah Lynn Arral, Anne Behre, Zihan Ling, Kathryn Ann Whitehead, Adam Walter Feinberg, Xi Ren and Si-Yang Zheng**

Supporting Information:
Synthetic Strategy for mRNA Encapsulation and Gene Delivery with Nanoscale Metal-Organic Frameworks

Harrison D. Lawson[1,2], Huy H. Nguyen[1] Keng-Jung, Lee[1], Nattarat Wongsuwan[1], Ayesha Y. Tupe[1,2], Mengrou Lu[1], Mariah L. Arral[2], Anne Behre[1], Zihan Ling[1], Kathryn A. Whitehead[1,2], Adam W. Feinberg[1,3], Xi Ren[1], and Si-Yang Zheng[1,4,]*

[1] Carnegie Mellon University, Biomedical Engineering Department, 5000 Forbes Avenue, Pittsburgh, PA 15213, United States
[2] Carnegie Mellon University, Chemical Engineering Department, 5000 Forbes Avenue, Pittsburgh, PA 15213, United States
[3] Carnegie Mellon University, Material Science and Engineering Department, 5000 Forbes Avenue, Pittsburgh, PA 15213, United States
[4] Carnegie Mellon University, Electrical and Computer Engineering Department, 5000 Forbes Avenue, Pittsburgh, PA 15213, United States

[*] Corresponding Author(s)

Corresponding Author(s):

Si-Yang Zheng
Department of Biomedical Engineering, and Electrical and Computer Engineering, Carnegie Mellon University
5000 Forbes Avenue
Scott Hall 4N211
Pittsburgh, PA 15213
siyangz@andrew.cmu.edu
(412)-268-3684

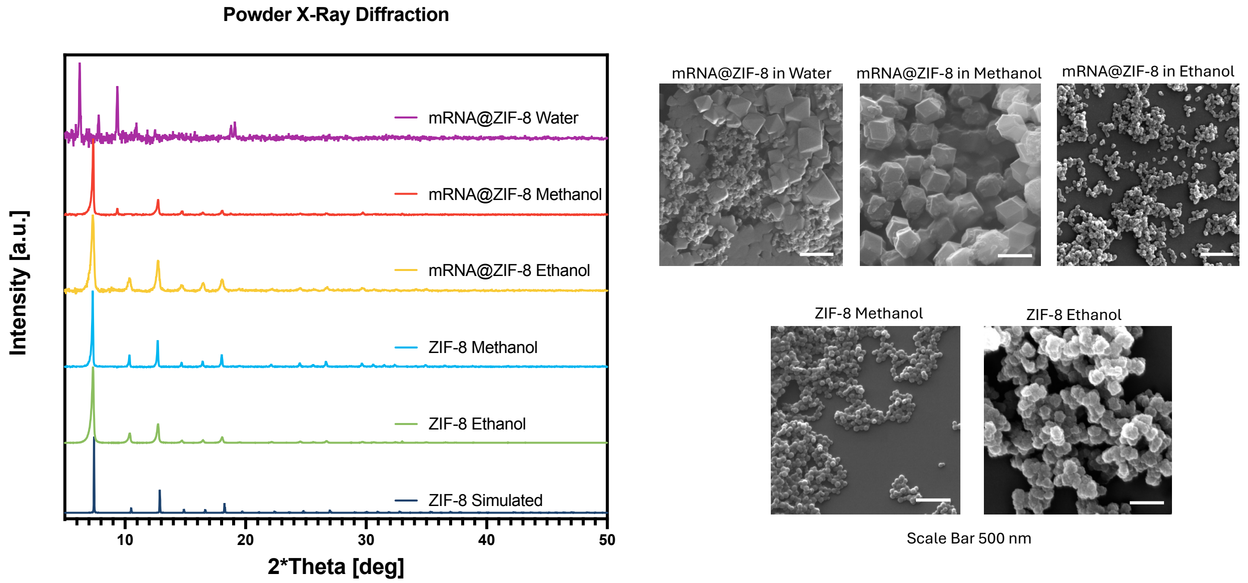

Figure S1. mRNA encapsulation within ZIF-8 was performed in water, ethanol, and methanol. The generated mRNA@ZIF-8 and literature-synthesized ZIF-8 were imaged, and their diffraction patterns were obtained. **(Left)** Powder X-ray Diffraction and matching **(Right)** SEM images of isolated ZIF-8 and mRNA@ZIF-8 particles. Scale bars = 500 nm. mRNA@ZIF-8 formed in methanol and ethanol shows the distinct dodecahedron shape of literature-synthesized ZIF-8, while water-synthesized mRNA@ZIF-8 demonstrates several morphologies. The PXRD patterns corroborate with our morphology findings, as ethanol and methanol synthesized mRNA@ZIF-8 have matching diffraction patterns, compared to simulated and literature-synthesized ZIF-8. On the other hand, mRNA@ZIF-8 synthesized in water does not match the reference ZIF-8 patterns.

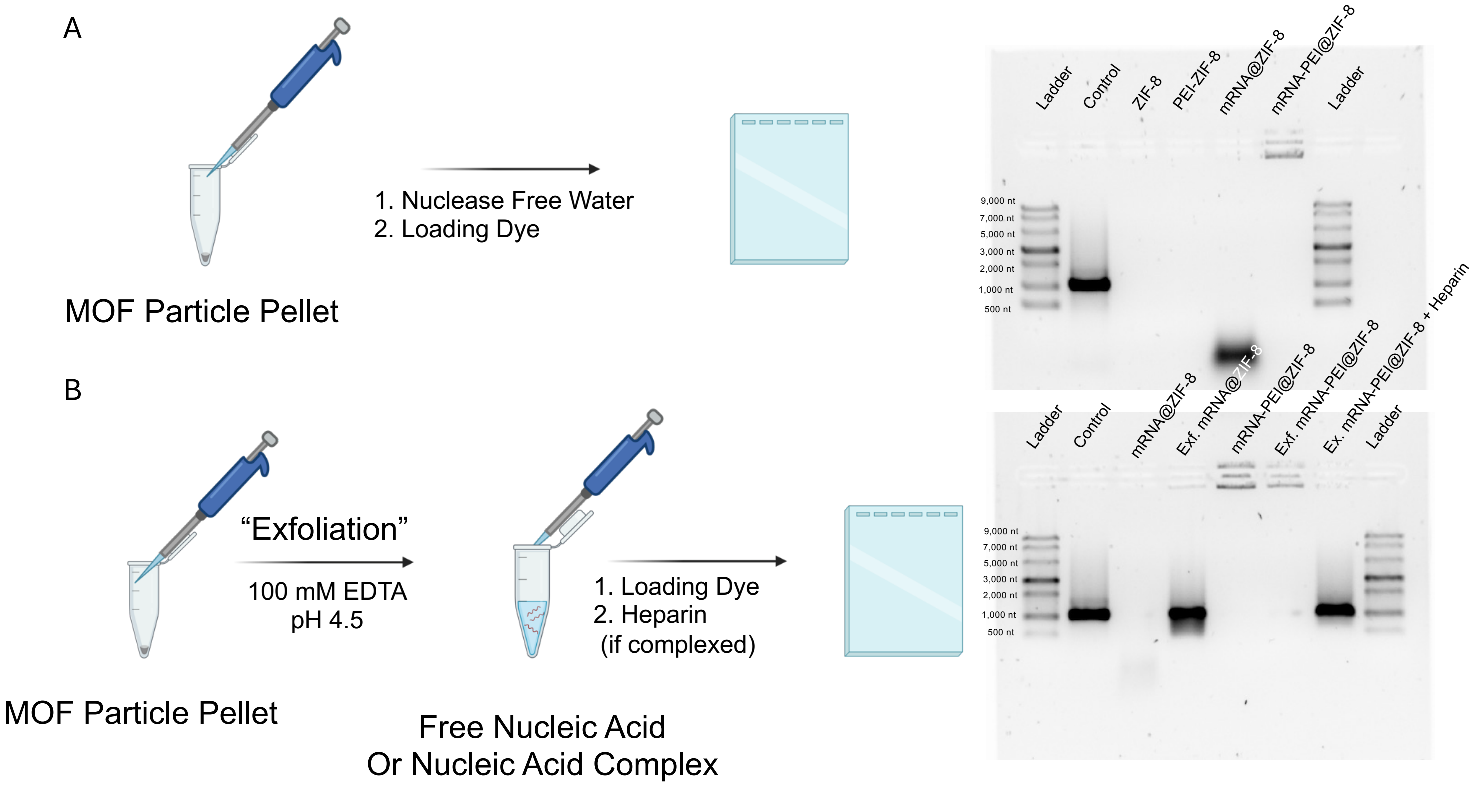

Figure S2. Exfoliation (Exf.) plays a vital role in preserving the integrity of mRNA when releasing it from mRNA@ZIF-8, as exposure to water alone leads to mRNA degradation in the presence of ZIF-8. Panel **(A)** shows the preparation of a MOF pellet for agarose gel electrophoresis, highlighting the resulting degradation of the mRNA in the absence of EDTA. Panel **(B)** illustrates the exfoliation process, where EDTA is used to strip the MOF coating from the mRNA before gel preparation. The use of EDTA is particularly important, as it prevents mRNA degradation during this process.

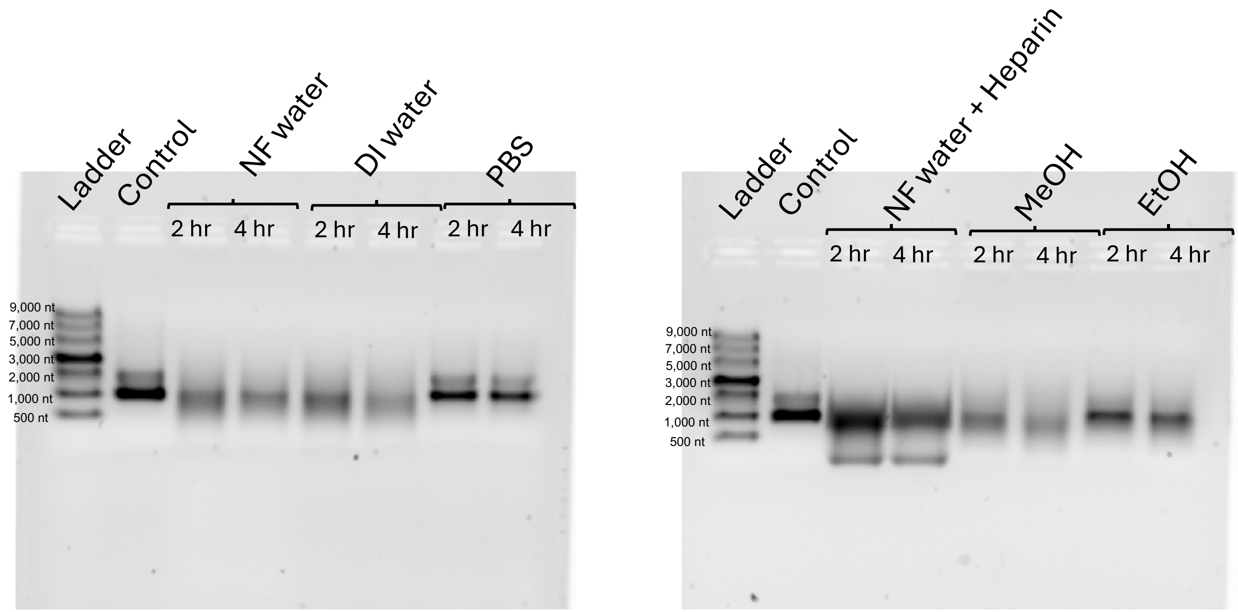

Figure S3. Native RNA gel after mRNA (1 μg) was incubated in various solvents for 2 and 4 hours in the presence of ZIF-8 (20 μg). Nuclease-free (NF) water and Deionized (DI) water both show degradation of mRNA. When adding heparin (50 μg), the mRNA shows slightly less degradation possibly due to the heparin blocking the mRNA from interacting with ZIF-8. Methanol (MeOH) also shows mRNA degradation in the presence of ZIF-8. Notably, PBS and EtOH preserve the mRNA length when used as the co-incubation solvent.

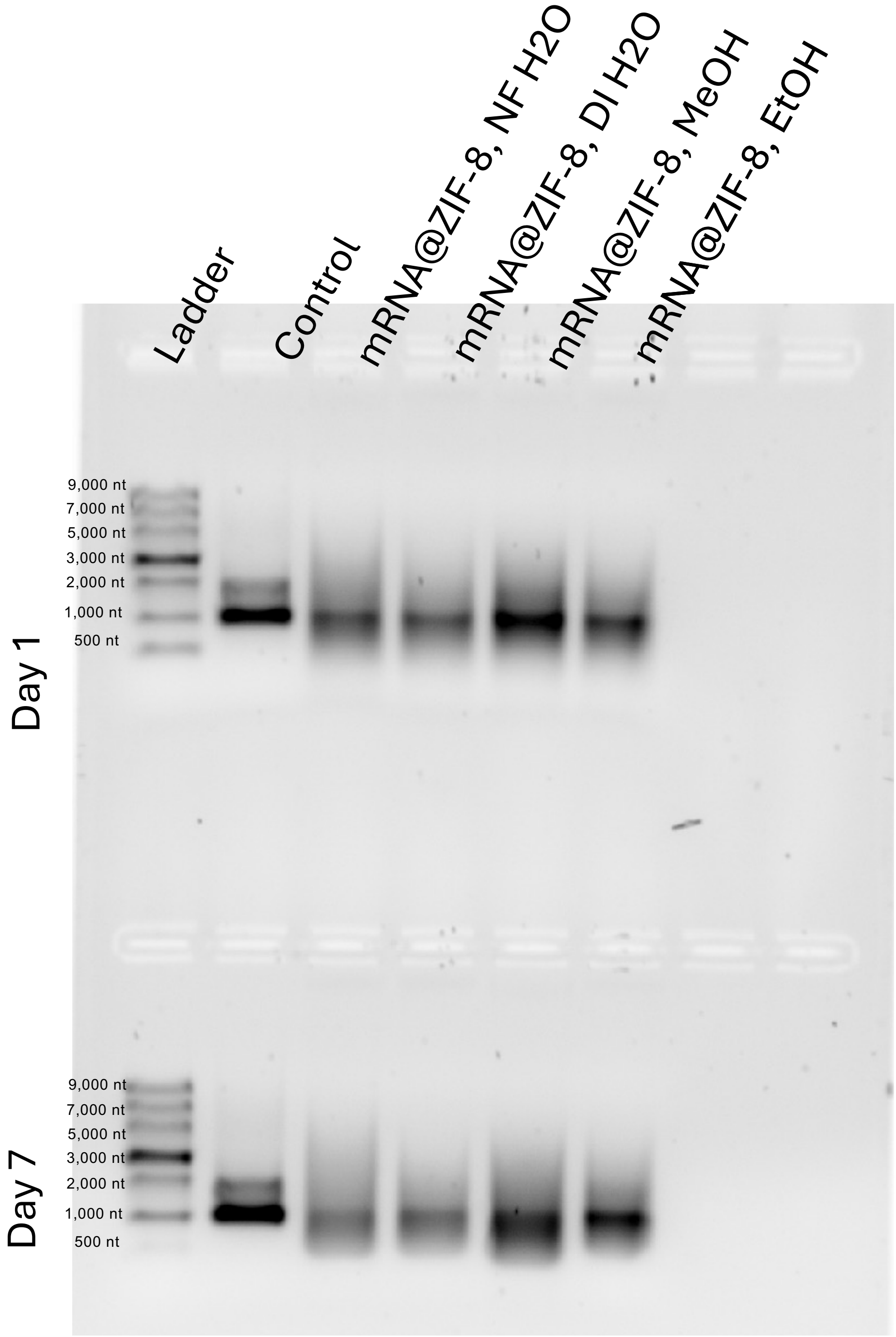

Figure S4. Native RNA gel after mRNA was encapsulated in ZIF-8 using NF water, DI water, MeOH, or EtOH then dried and stored under a vacuum at room temperature. The gel shows significant mRNA degradation in all solvents except ethanol.

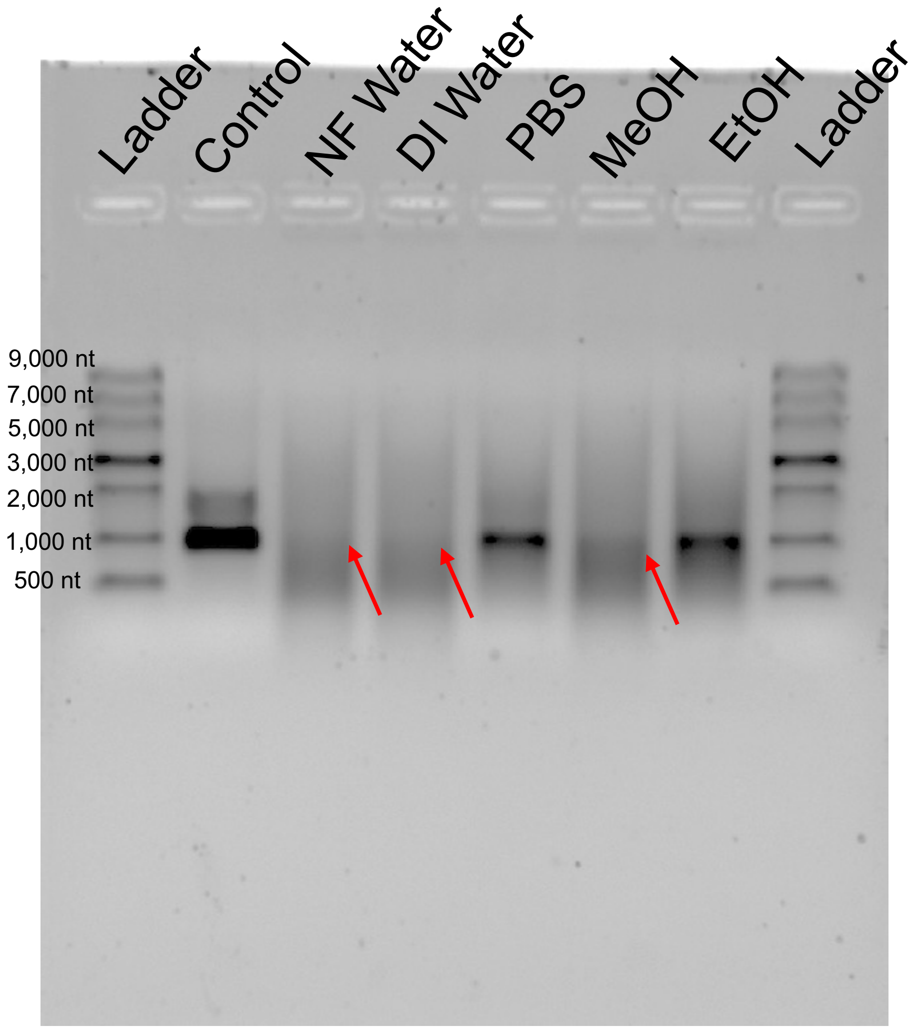

Figure S5. Native RNA gel after mRNA@ZIF-8 was exposed to various solvents for 2 hours and then exfoliated with EDTA. mRNA inside of mRNA@ZIF-8 is not stable in unbuffered aqueous conditions and MeOH (degradation noted with red arrows). mRNA inside mRNA@ZIF-8 is stable in PBS as well as EtOH.

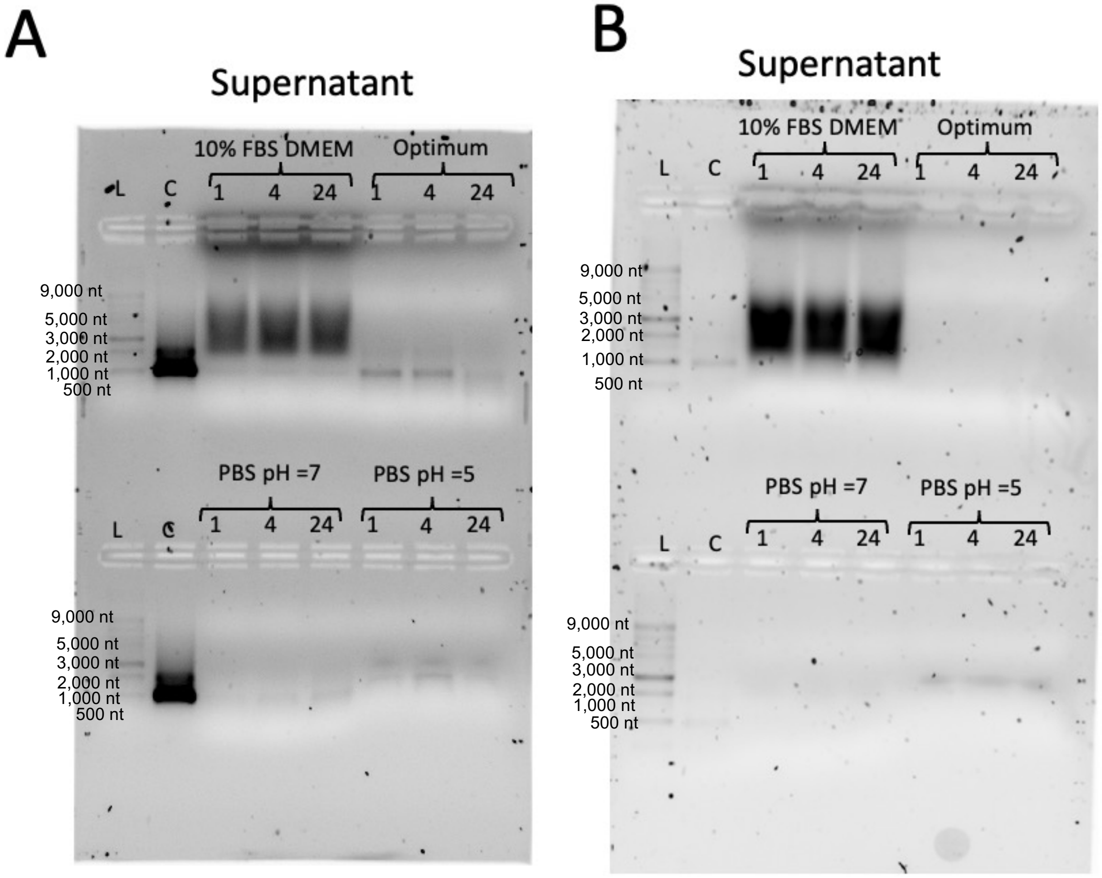

Figure S6. Native RNA gel of biological medium supernatants after **(A)** mRNA@ZIF-8 and **(B)** mRNA-PEI@ZIF-8 being incubated with 10% FBS DMEM, Opti-MEM, and PBS pH 7 and pH 5. Note that those high molecular weight bands are from the nucleic acids inside the FBS. mRNA@ZIF-8 supernatants show a weak, but distinct band at the control mRNA's length. Interestingly, PBS at pH 7 shows the weakest band intensities. In comparison, mRPZ showed minimal mRNA leakage, as only light bands appeared in the supernatant fluid of pH 5 PBS. This alludes to mRPZ retaining some pH-triggered release due to its ZIF-8 coating but possibly PEI incorporation improving the stability of the ZIF-8 shell against Zn binders.

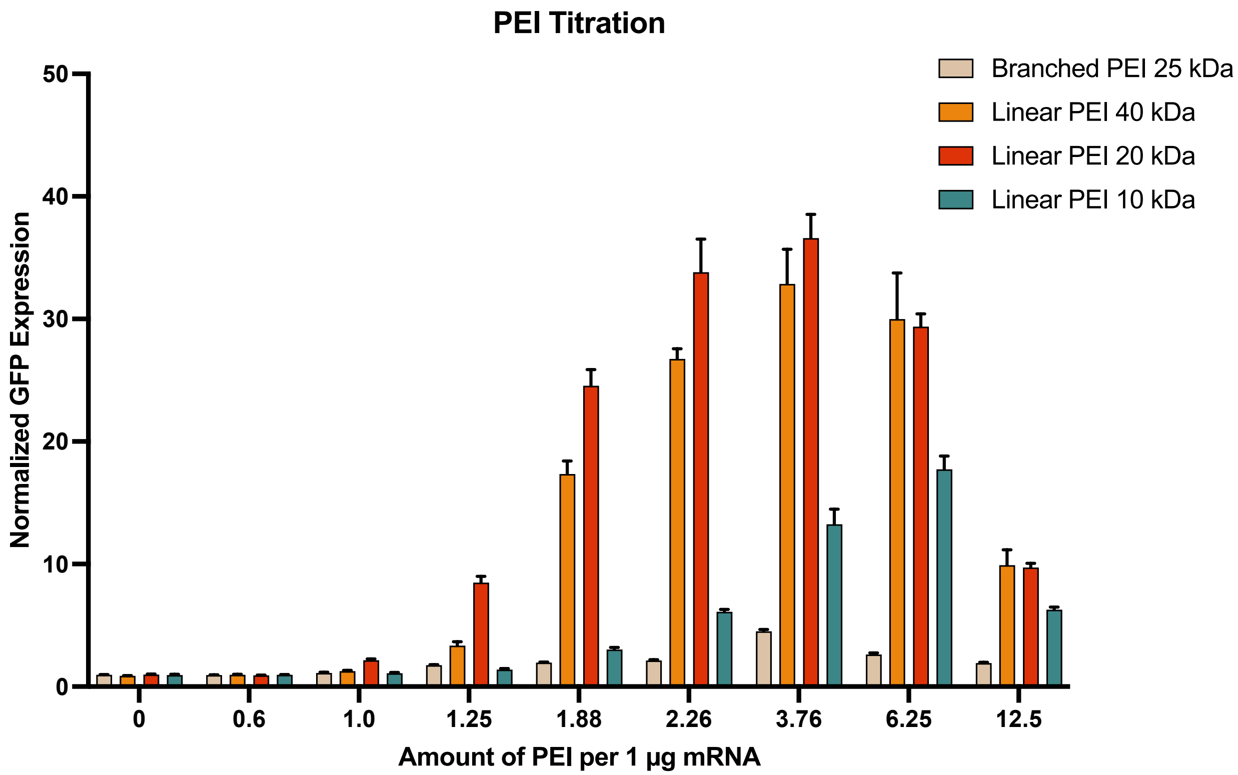

Figure S7. HEK293t cells were transfected in serum media with mRNA complexed with varying amounts of PEI isoforms complexed in Opti-MEM for 10-15 min then applied to cells in a 96-well plate with 100 ng mRNA per well. The cells were fixed and GFP expression was measured at 48 hours post-transfection and normalized to the DAPI signal. Results demonstrate that 20 and 40 kDa Linear PEI perform better than 10 kDa Linear and 25 kDa Branched PEI. The best range for GFP expression is between a weight ratio of 2.26 and 3.76 µg of PEI per µg mRNA. Data presented as mean +/- standard error of the mean (StdEM) and sample sizes are N ≥ 7.

1 µg mRNA

mRNA Strand

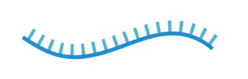

$$ssRNA\ Molecular\ Weight = Length\ (nt) * 320.5 + 159.0\ [\frac{g}{mol}]$$

*Assumes 1 nt = Phosphate

$$Phosphorous = \left[RNA\ MW * \frac{1\ mol}{6.022e23\ molecules} * \frac{10^6\ \mu g}{1\ g} * \frac{1\ molecule}{\#\ of\ Phosphates}\right]^{-1} \left[\frac{\text{Phosphorous}}{\mu\text{g RNA}}\right]$$

1 µg Linear PEI (monomer weight 43 g/mol)

PEI Monomer

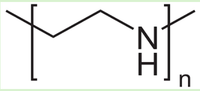

C – 2
N – 1
H – 5
MW = 43 g/mol

MW	Monomers	Nitrogen
20,000 kDa ->	465.1 monomers ->	1.40e16 Nitrogen
25,000 kDa ->	581.4 monomers ->	1.40e16 Nitrogen

$$Nitrogen = \#Monomers * \frac{6.022e23}{MW\ of\ Poylmer} \left[\frac{Nitrogen}{\mu g\ PEI}\right]$$

1 µg Branched PEI (MW = 533.8 g/mol, PubChem, C24H63N13)

Branched PEI Monomer

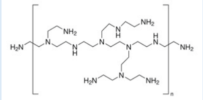

C – 24
N – 13
H – 63
MW 533.8 = g/mol

MW	Nitrogen
25,000 kDa ->	1.47e16 Nitrogen

$$Nitrogen = \mu g\ PEI * 1.47e16 \left[\frac{Nitrogen}{\mu g\ PEI}\right]$$

$$Nitrogen\ to\ Phosphate\ Ratio = \frac{(\#\ Nitrogen\ in\ 1\ \mu g\ PEI)(Weight\ of\ PEI\ in\ \mu g)}{(\#\ Phosphorous\ in\ 1\ \mu g\ ssRNA) * (Weight\ of\ ssRNA\ in\ \mu g)}$$

Calculation 1. Calculation describing the arithmetic behind the nitrogen-to-phosphate (or phosphorous) ratio.

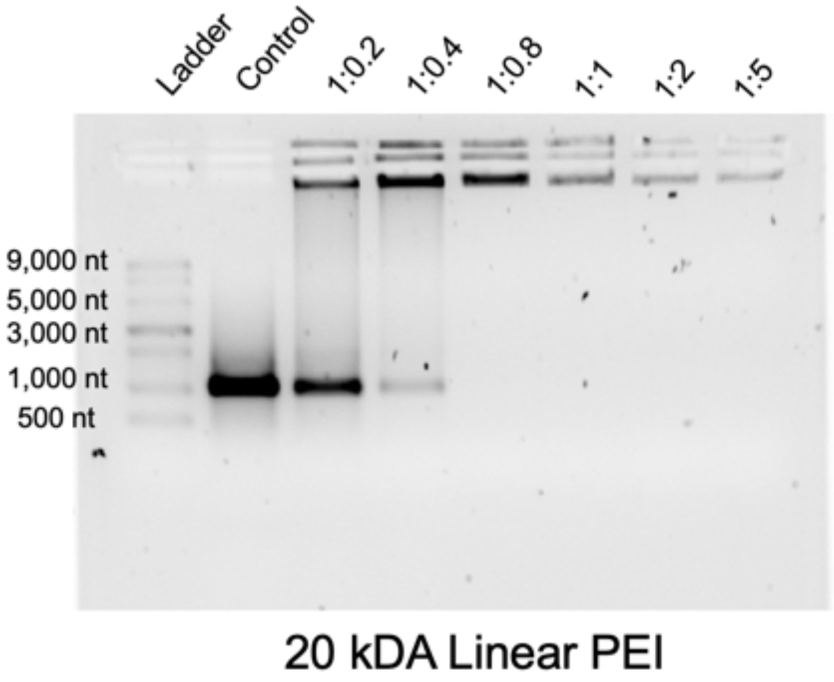

Figure S8. Retardation of mRNA with various weight ratios of RNA: PEI demonstrates the minimum amount of PEI needed to bind mRNA. Briefly, RNA was mixed with PEI and incubated for 15 mins. The complexes were run on a native RNA gel. Fully bound RNA is retained in the wells, as seen in all samples except the control. At a weight ratio of (RNA: PEI) 1:0.8, all RNA is retained in the well. This weight ratio represents a nitrogen-to-phosphate ratio of 6.

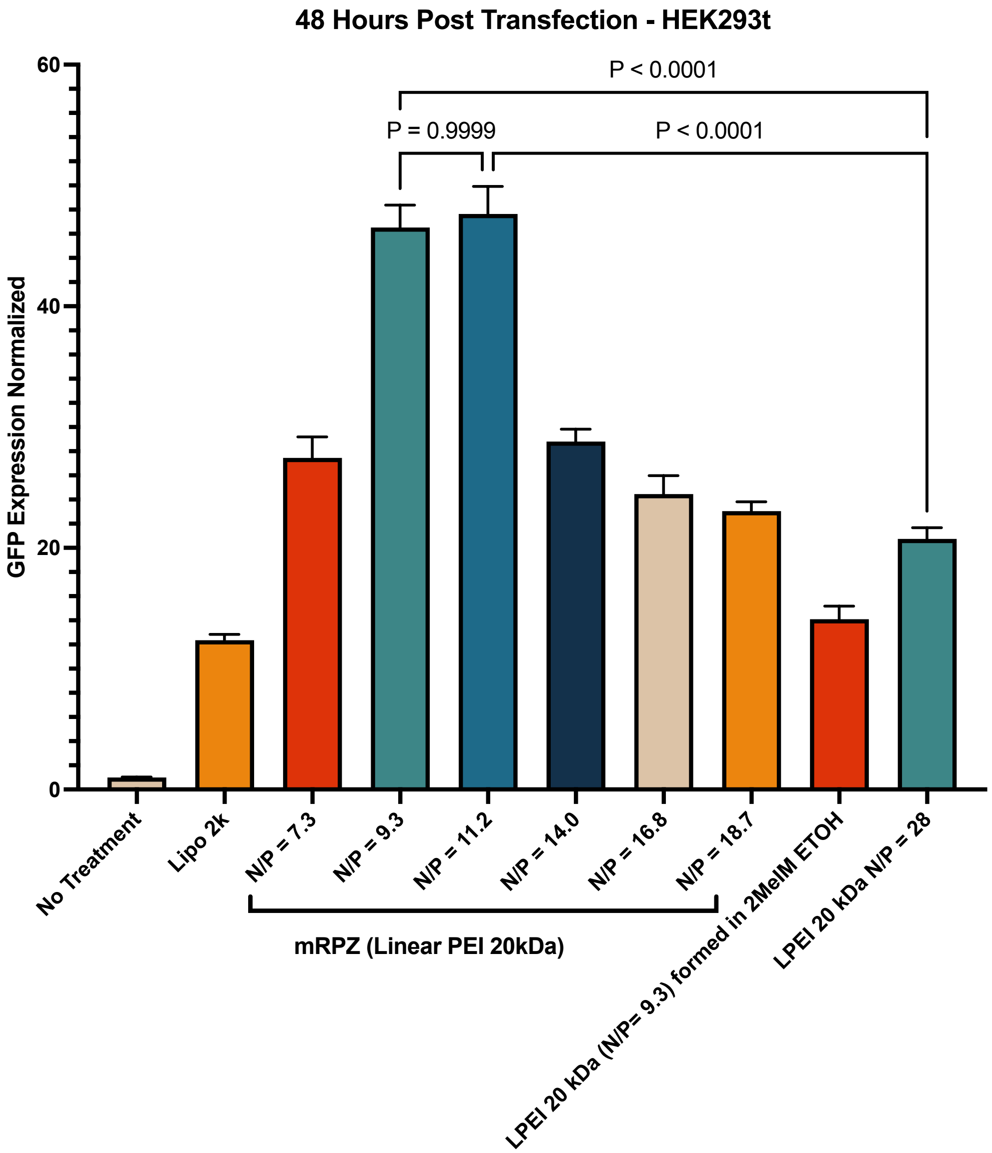

Figure S9. *In vitro,* GFP expression titration with PEI in mRNA-PEI@ZIF-8 (mRPZ) expressed as nitrogen-to-phosphate ratios (N/P). mRNA-PEI@ZIF-8, with different N/P ratios, were suspended in Opti-MEM and delivered to HEK293t cells plated in a 96-well plate with 100 ng of mRNA per well. Encapsulation within ZIF-8 improves mRNA expression significantly over the best-performing mRNA-PEI complex (N/P ratio of 28, 3.76 µg of PEI per µg mRNA) at a lower N/P ratio of 9.3 (1.25 µg PEI per µg mRNA). Additionally, we show that the complexation of mRNA-PEI in ZIF-8 2-MeIM precursor does not elicit a high GFP expression, aiding support for the ZIF-8 coating improved the delivery of mRNA. Data presented as mean +/- StdEM, the sample size is N = 16 except for Lipo 2k control N=7, and statistical significance (P) was assessed using one-way ANOVA.

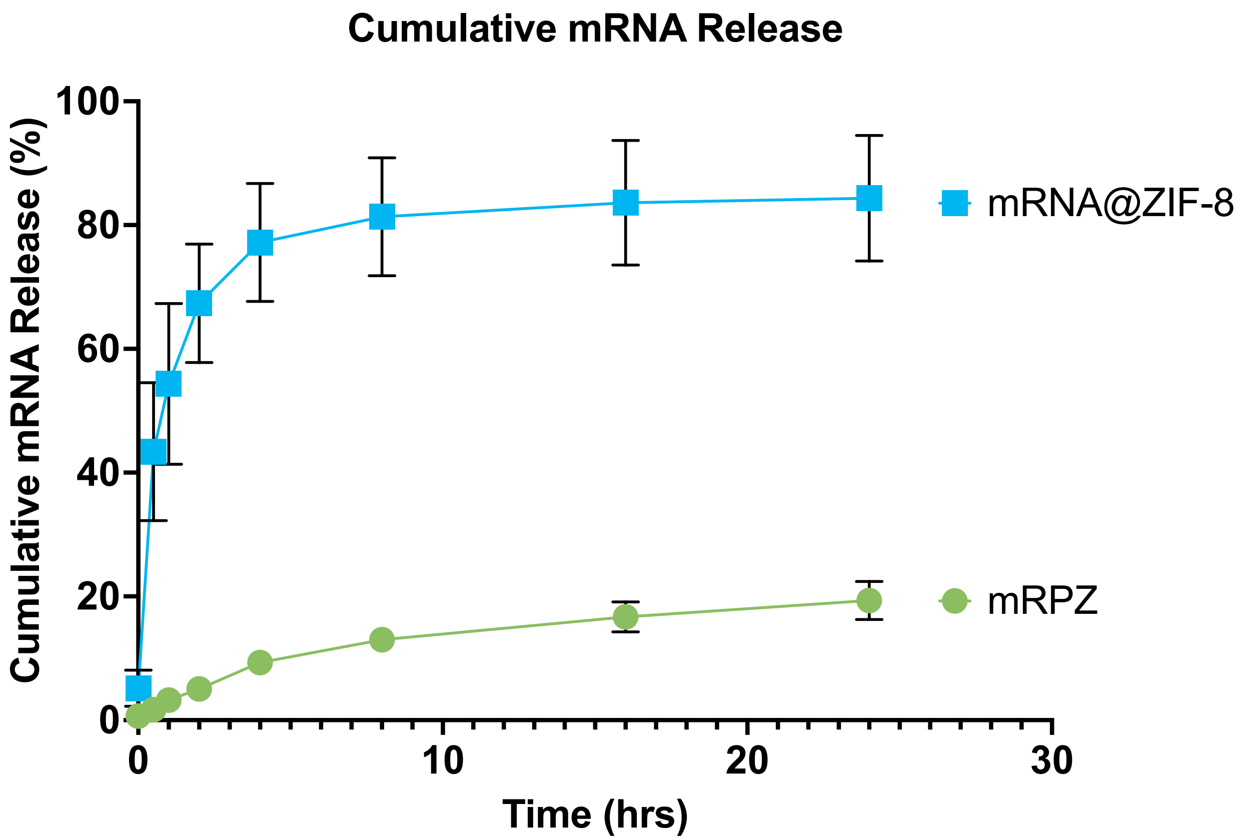

Figure S10: Cumulative mRNA release from mRNA@ZIF-8 and mRPZ over 24 hours in PBS supplemented with 10% FBS. Cy5 fluorescence was used to quantify mRNA released into the supernatant. Data represent mean ± StdEM (N=3).

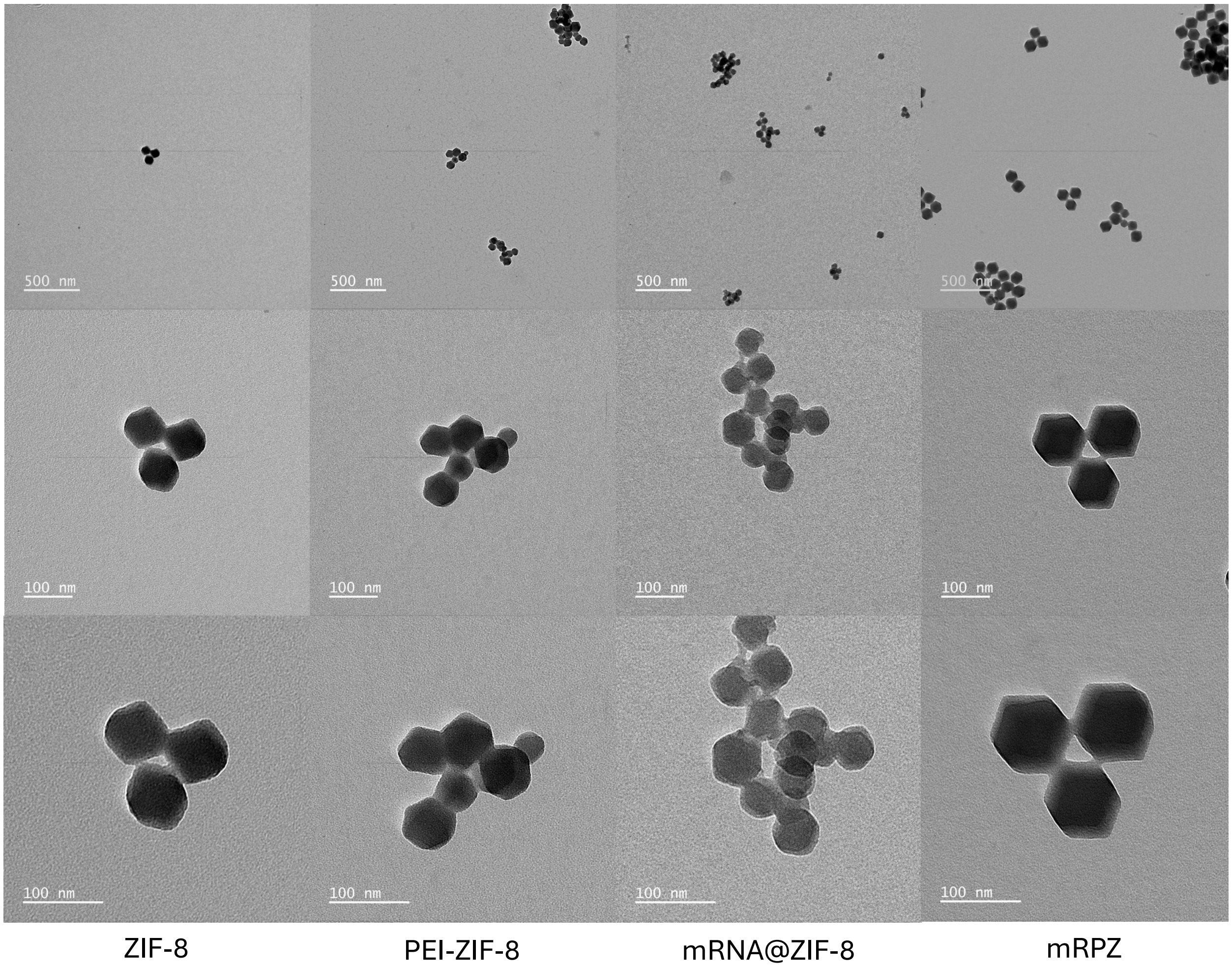

Figure S11. Transmission electron microscopy (TEM) images were collected of ZIF-8, PEI-ZIF-8, mRNA@ZIF-8, and mRPZ. TEM was performed at 80 kV with a spot size of 6 to reduce beam damage to the particle. The top row scale bar is 500 nm, and the subsequent rows have a 100 nm scale bar. TEM micrographs show the distinct dodecahedron shape of ZIF-8 as well as a uniform inner atomic weight distribution (based on intensity). Interestingly, PEI appeared to improve the stability of ZIF-8 under the TEM beam.

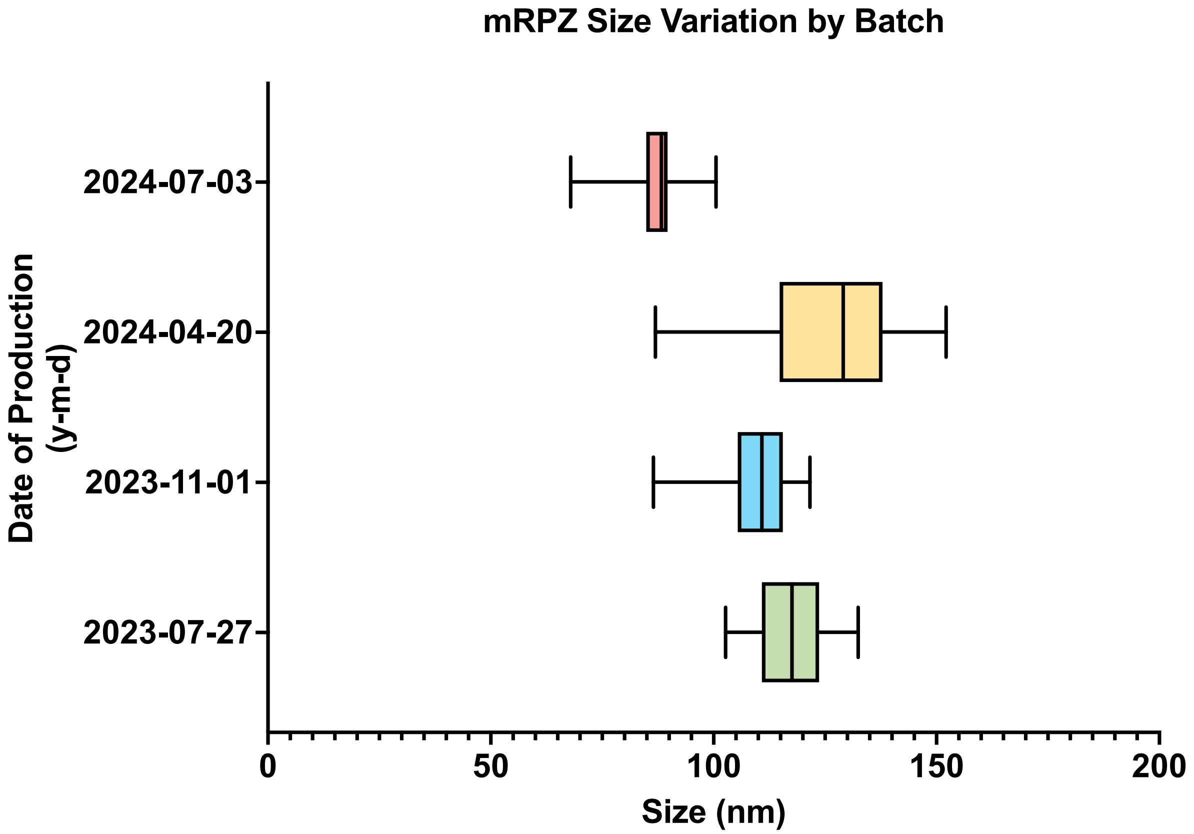

Figure S12. The size distribution measured by SEM demonstrates some batch-to-batch variability in the particle size. The data are presented as the minimum, 25th percentile, median, 75th percentile, and maximum with a sample size of N=20.

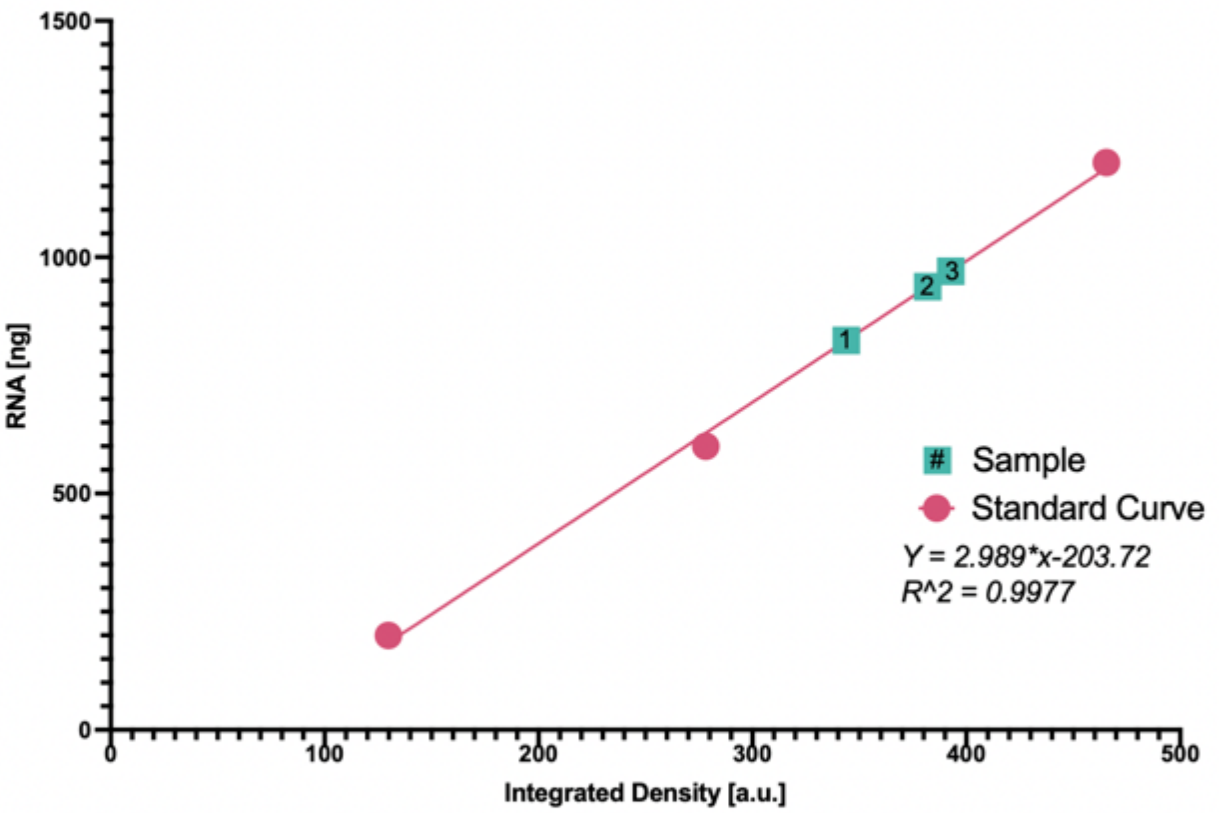

Figure S13. The loading efficiency was determined using the Native RNA Gel featured in **Figure 4A** after exfoliation and release of mRNA from mRPZ. The intensity of the resultant mRNA standard was used to create a standard curve. The linear regression equation was used to calculate the amount of mRNA successfully encapsulated in mRPZ. The amount encapsulated was used to estimate a loading efficiency of 91.1% +/- 7.7% for mRPZ.

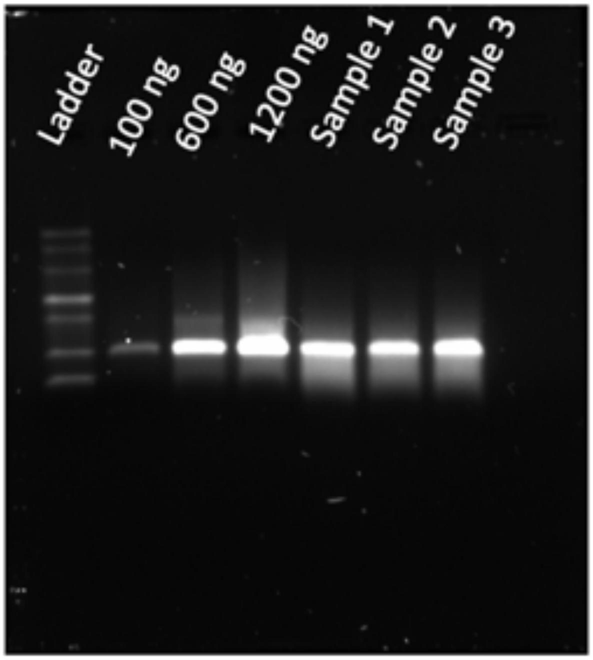

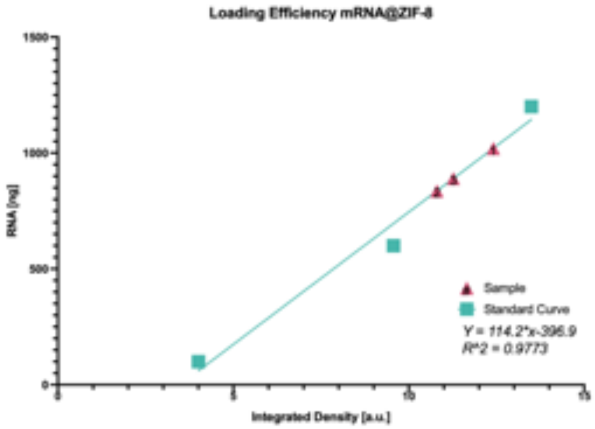

Sample	RNA [ng]	Loading Efficiency (%)
1	890	89.0%
2	837	83.7%
3	1020	102.0%
**Average**	**915 +/- 94.6**	**91.5% +/- 9.5%**

Figure S14. Loading efficiency of mRNA@ZIF-8 determined by image analysis of (**Left**) native RNA gel using (**Right**) a standard curve. The loading efficiency calculated based on gel intensities was 91.5%.

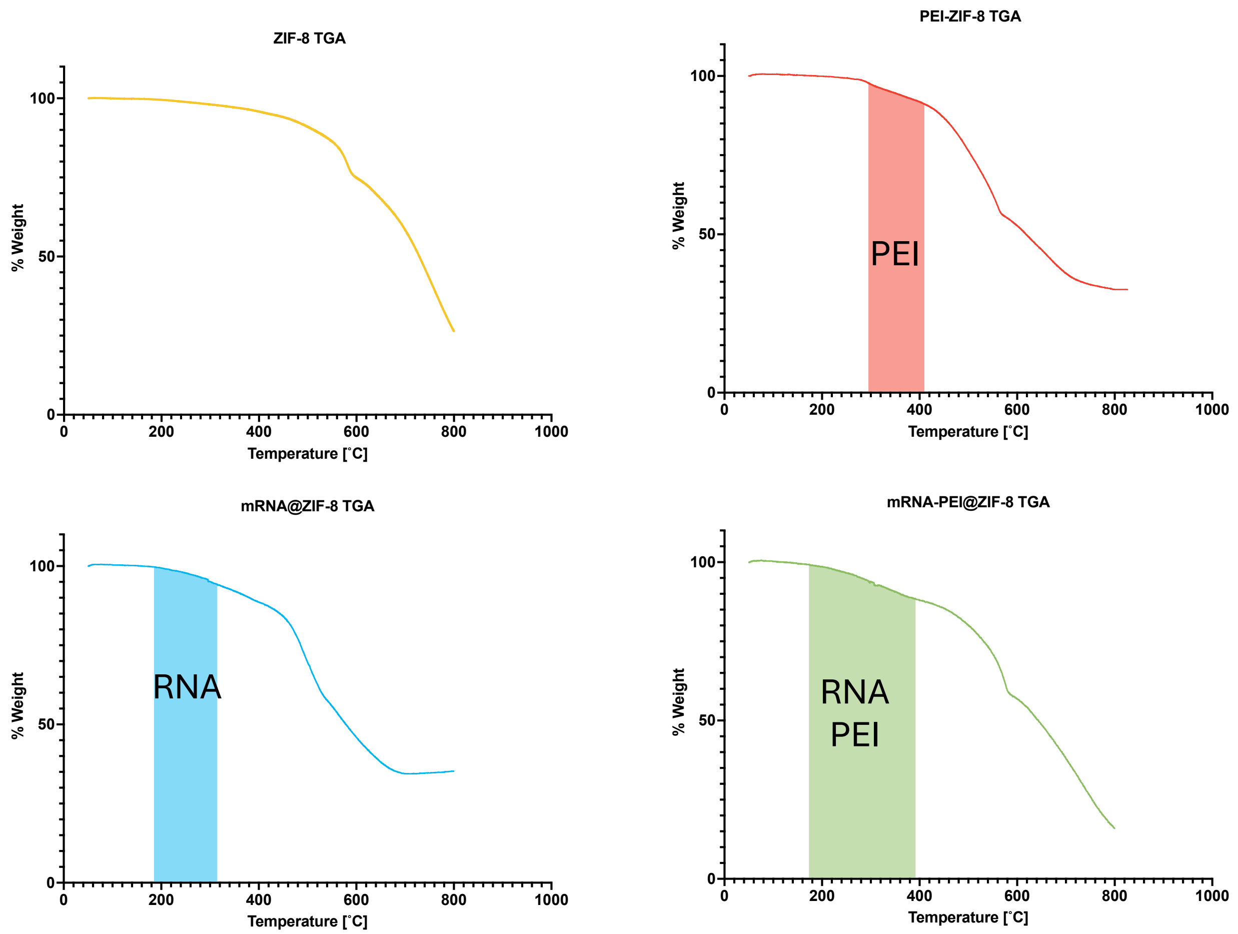

Figure S15. Thermogravimetric Analysis (TGA) of ZIF-8 composite particles with individual components labeled.

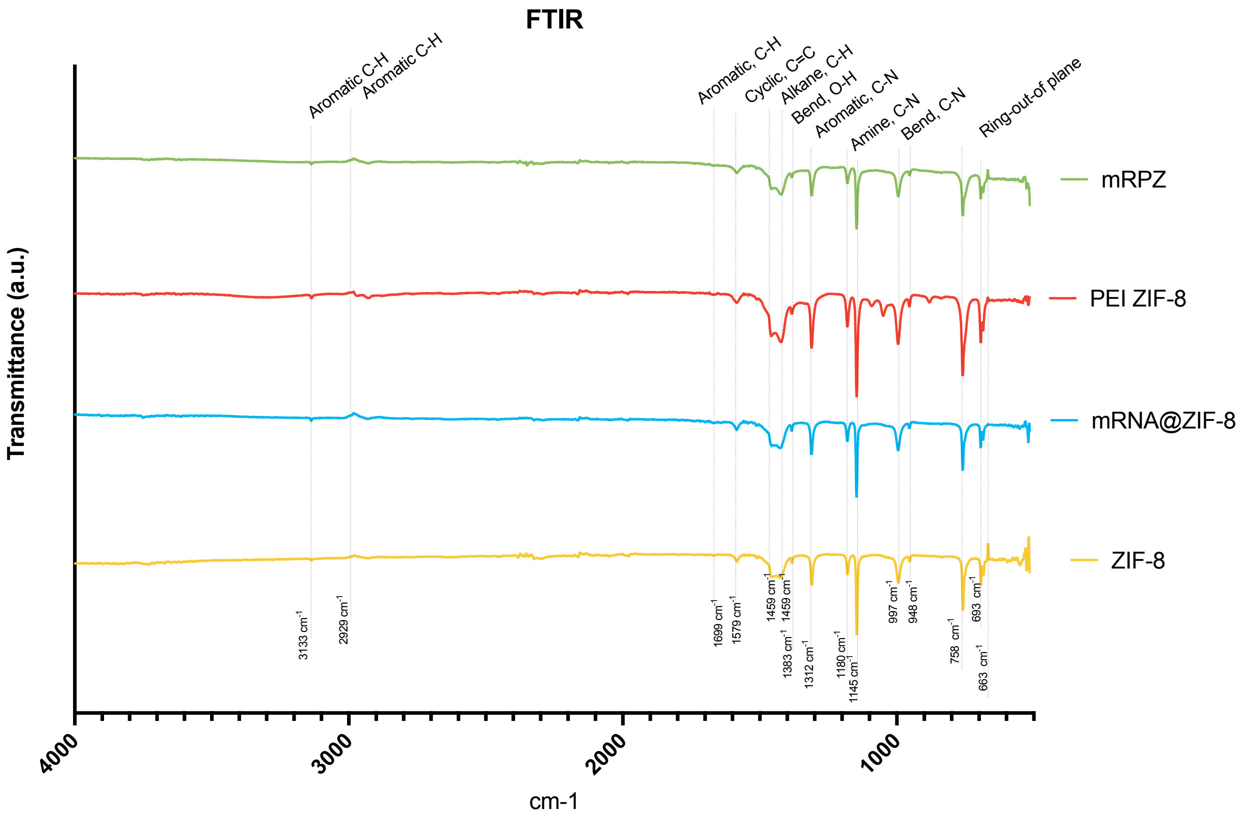

Figure S16. Fourier Infrared Spectroscopy was performed on mRPZ and comparison particles. All demonstrate a similar composition. Interestingly, peaks are observed in the PEI-ZIF-8 fingerprint region that are absent in mRPZ. These may indicate the loss of bond stretching due to the binding of mRNA to PEI.

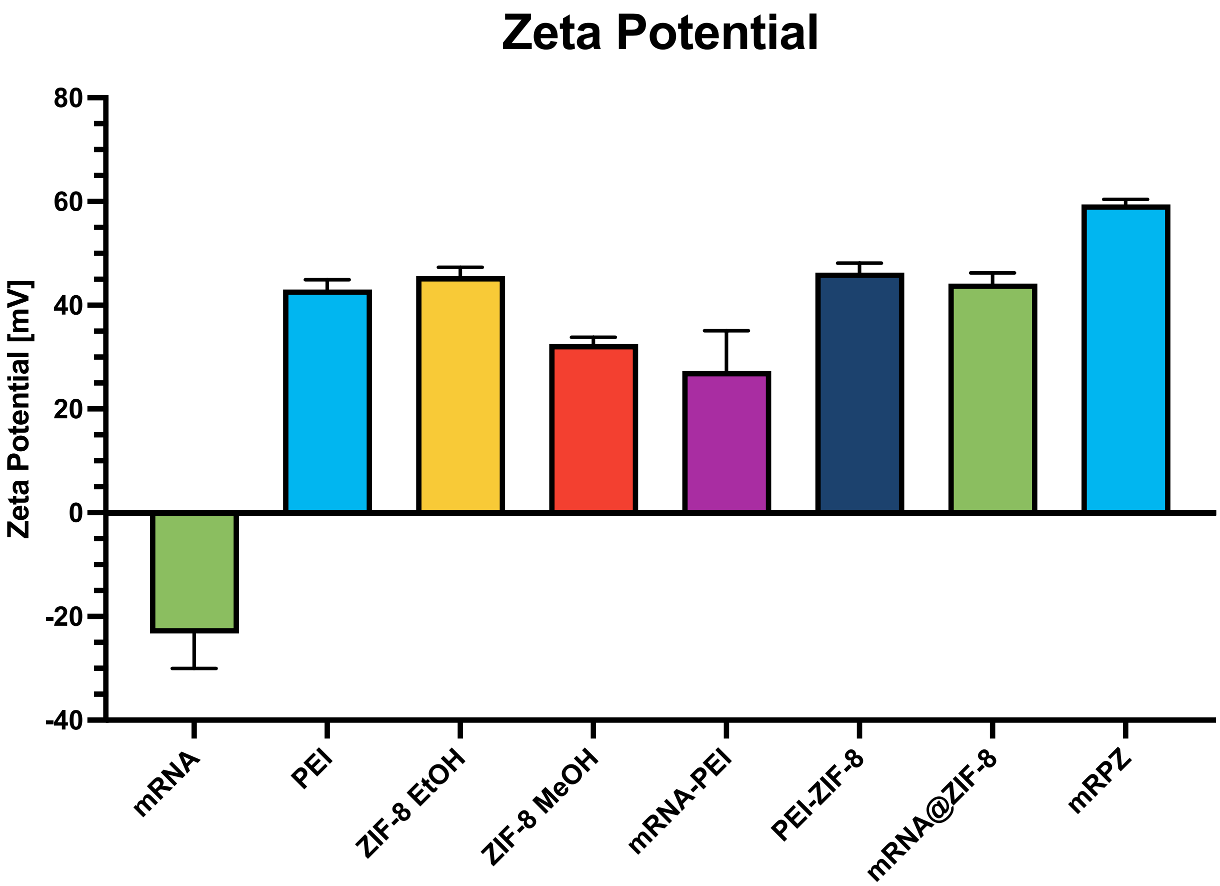

Figure S17. Zeta potential measures of individual components as well as composite ZIF-8 particles. Data presented as mean +/- StdEM and sample sizes are N ≥ 3.

A

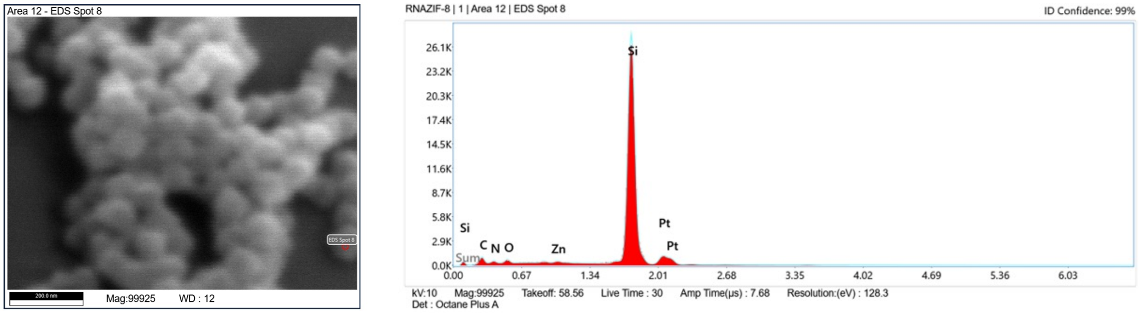

B

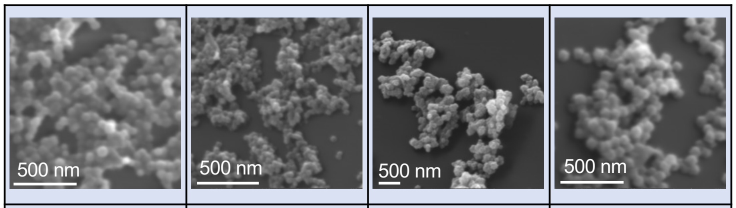

	ZIF-8	mRNA@ZIF-8	PEI-ZIF-8	mRPZ
**Carbon**	40.2 ± 5.6	47.8 ± 3.0	56.1 ± 2.4	46.0 ± 6.7
**Nitrogen**	2.8 ± 1.1	8.2 ± 1.1	12.2 ± 1.9	8.4 ± 3.1
**Zinc**	0.5 ± 0.1	0.4 ± 0.1	0.8 ± 0.1	0.5 ± 0.2
**Oxygen**	4.3 ± 0.8	3.8 ± 0.5	3.8 ± 0.6	4.2 ± 0.9
**Silicon**	49.9 ± 6.7	37.9 ± 3.9	25.8 ± 4.6	38.8 ± 10.9
**Platinum**	2.3 ± 0.4	1.9 ± 0.4	1.3 ± 0.2	2.2 ± 0.6

C

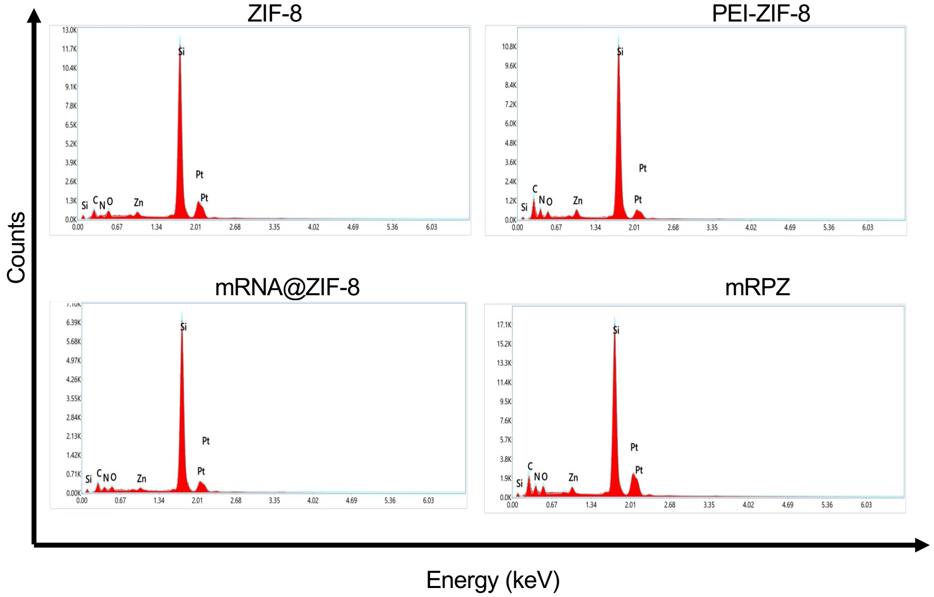

Figure S18. Energy dispersive spectroscopy spectrum produced from analyzing a single spot of mRNA-PEI@ZIF-8 drop cast on a silicon wafer and sputter coated with Platinum, **(A)** scale bar = 200 nm. The atomic percentage +/- error percentage was obtained for each composite particle type **(B)** as well as their EDS spectrum **(C)**. The SEM inlays above the atomic percentage data have a 500 nm scale bar.

A

Atomic % +/- Error

Element	ZIF-8	mRNA@ZIF-8	PEI-ZIF-8	mRPZ
Carbon	75.69 +/- 2.15	90.47 +/- 7.14	73.57 +/- 2.36	85.44 +/- 5.20
Nitrogen	11.93 +/- 2.12	4.37 +/- 1.00	14.49 +/- 2.49	10.02 +/- 2.08
Zinc	6.41 +/- 0.82	3.48 +/- 0.53	6.97 +/- 0.89	2.47 +/- 0.36
Phosphorous	0.06 +/- 0.06	0.07 +/- 0.07	0.05 +/- 0.02	0.02 +/- 0.00
Oxygen	5.92 +/- 1.18	1.62 +/- 0.38	4.92 +/- 0.95	1.37 +/- 0.29

B

Sample Spectrum, TEM EDS

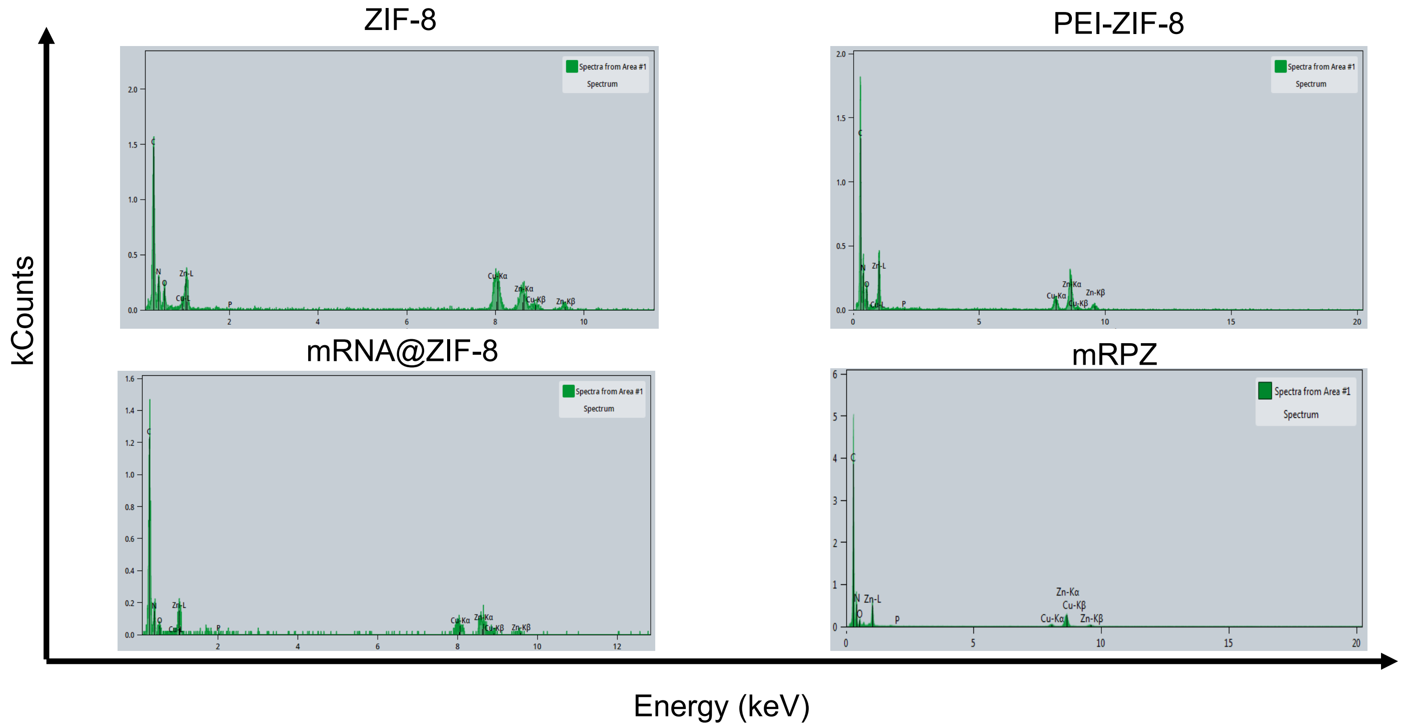

Figure S19. Energy dispersive spectroscopy atomic percentage (**A**) and spectrum (**B**) are produced by analyzing a single particle drop cast onto a carbon mesh grid.

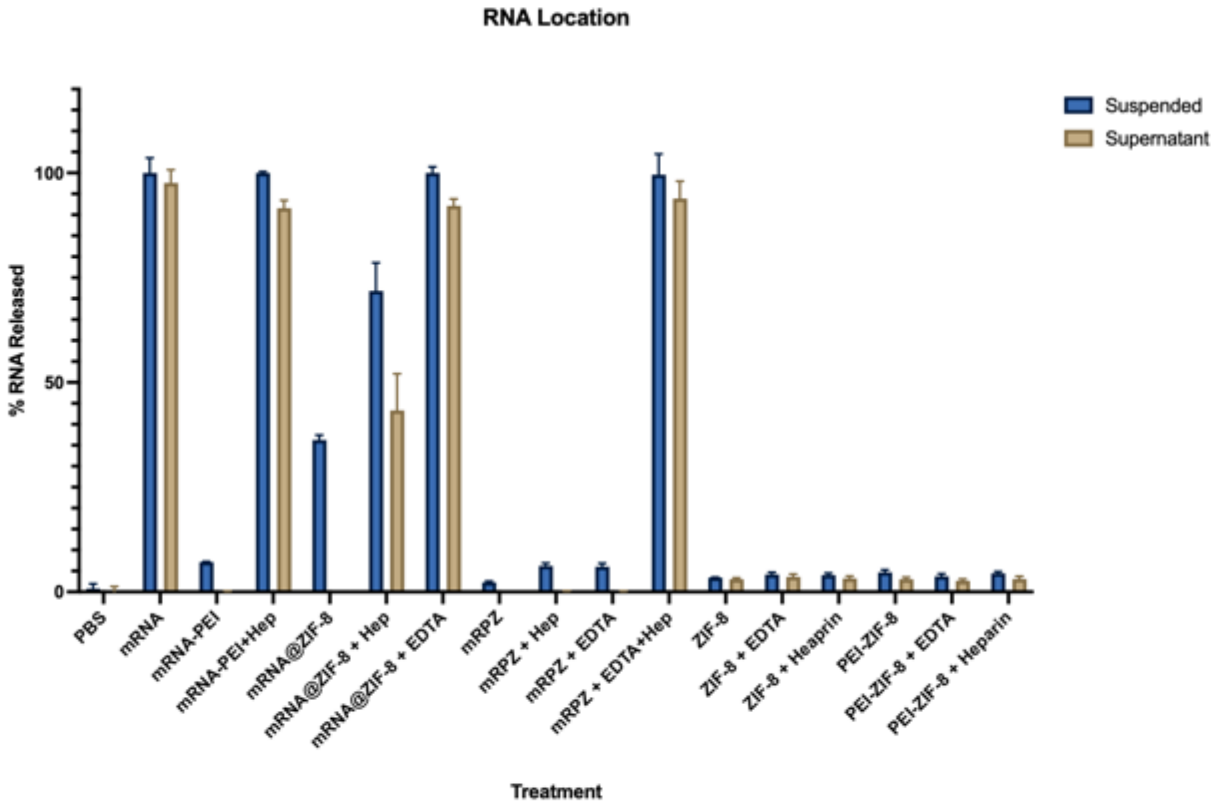

Figure S20. A molecular mRNA probe assay with complete controls showing little to no background signal comes from ZIF-8 or PEI-ZIF-8 in combination with treatments. Data presented as mean +/- StdEM and sample sizes are N=6, Experiment was completed in duplicate.

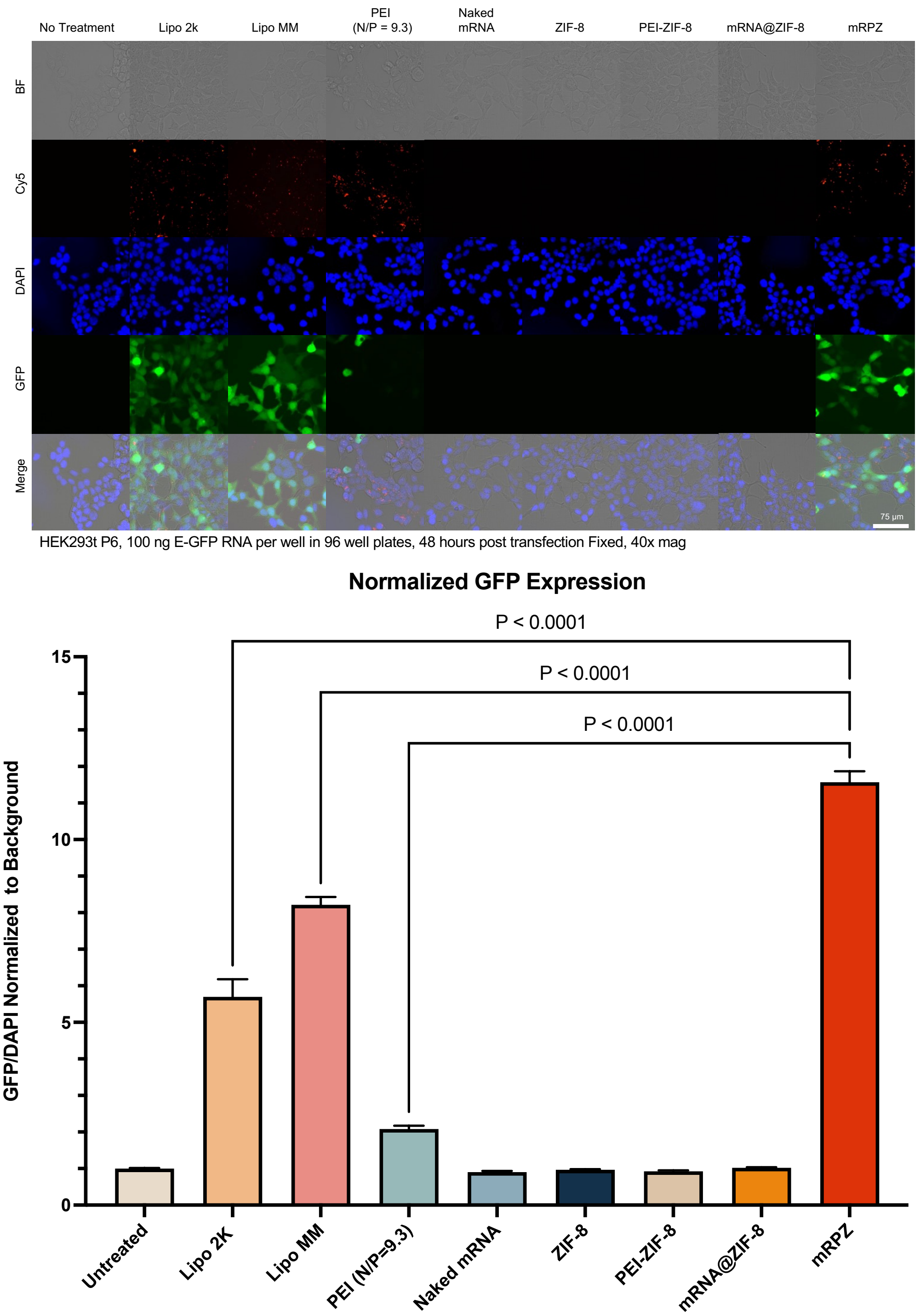

Figure S21. Transfection of HEK293t cells with controls imaged with fluorescence microscopy (**Top**) with GFP expression quantified with a plate reader (**Bottom**). Scale bars = 75 μm. Biological replicates were performed. Data presented as mean +/- StdEM, sample sizes are N ≥ 32, and statistical significance (P) was assessed using a one-way ANOVA.

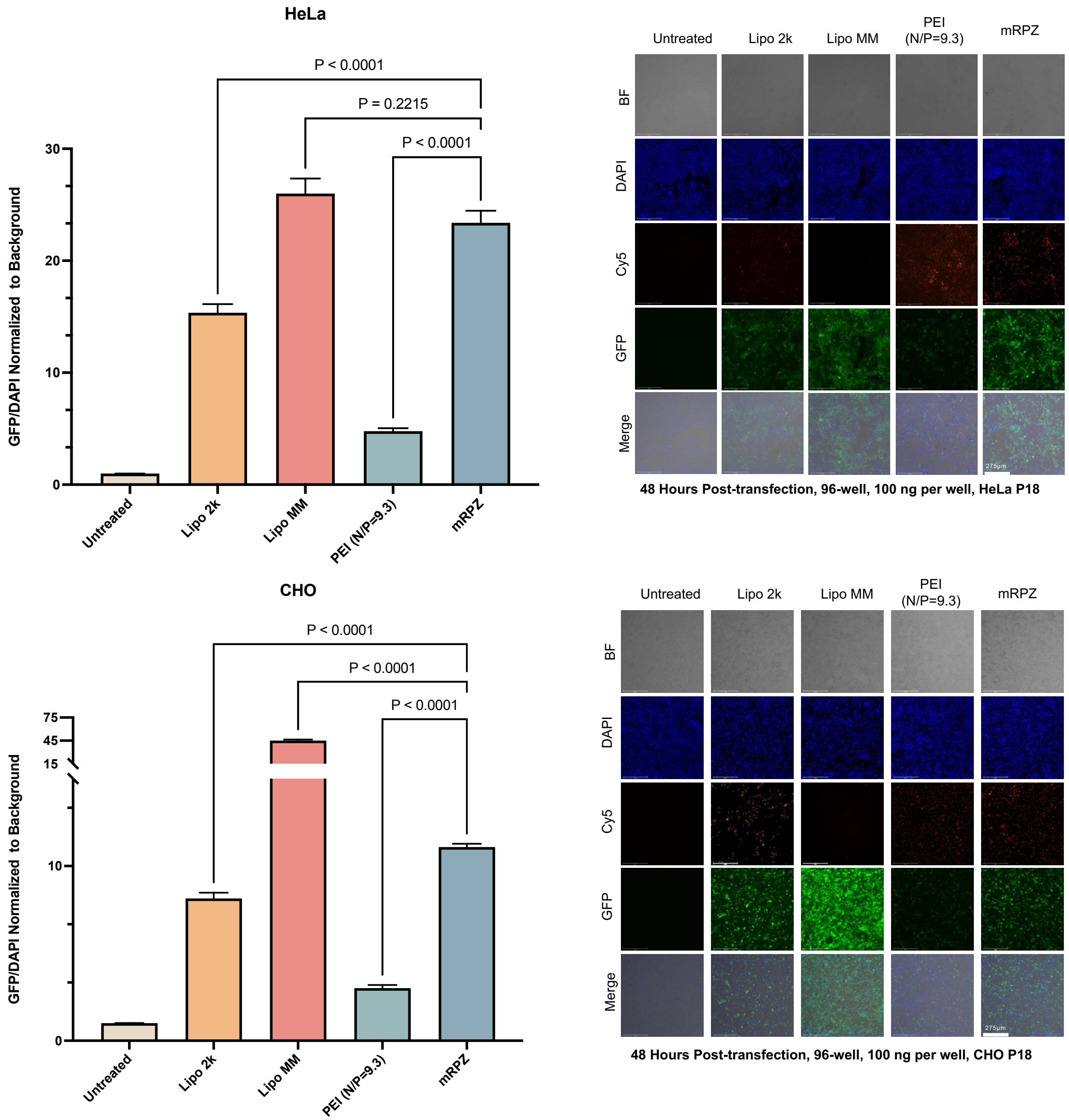

Figure S22. *In vitro* transfection with mRPZ particles loaded with eGFP-encoding mRNA. GFP expression was measured with a plate reader and images were taken with fluorescence microscopy for (**Top**) HeLa and (**Bottom**) CHO cells. Scale bars = 275 μm. Biological replicates were performed. Data presented as mean +/- SEM, sample sizes are as follows: untreated and mRPZ (N=32), all others (N=16), statistical significance (P) assessed with one-way ANOVA.

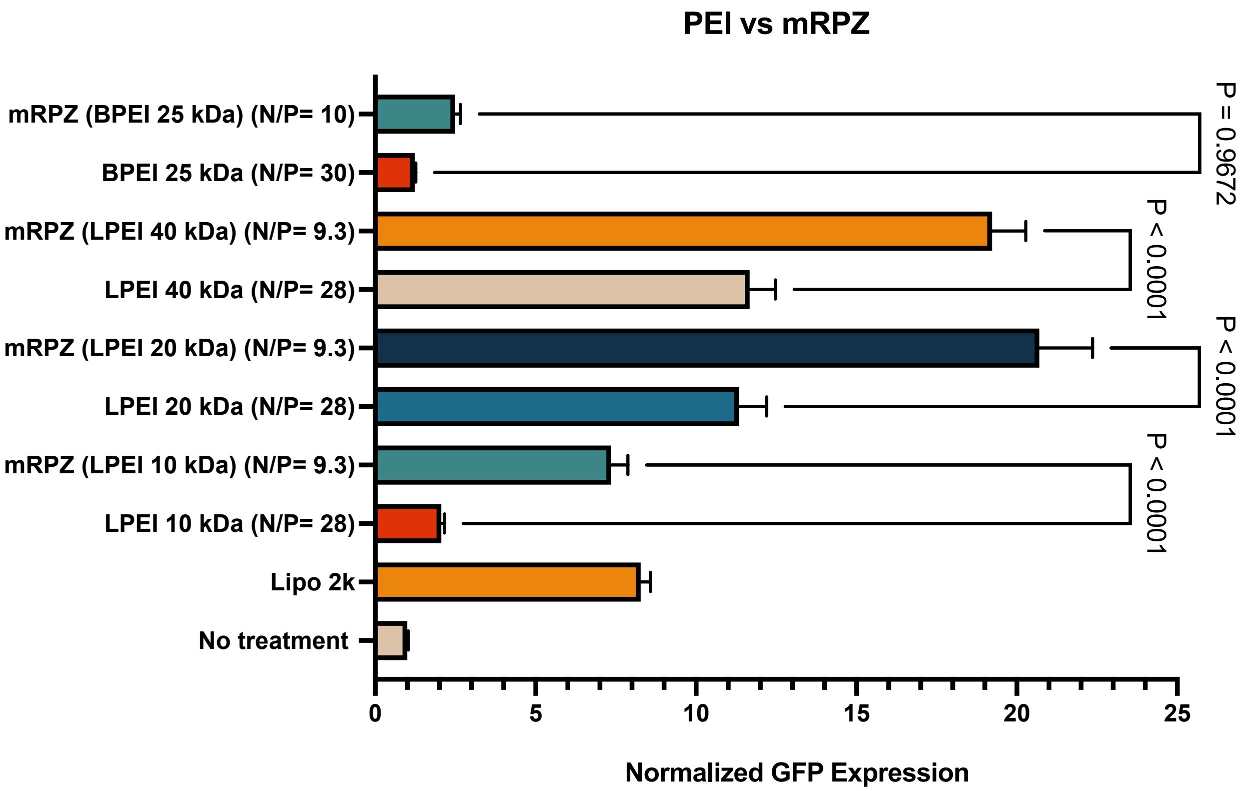

Figure S23. *In vitro* transfection with mRPZ particles loaded with eGFP-encoding mRNA with an optimized N/P ratio of 9.3-10 compared to mRNA-PEI particles containing different isoforms of PEI and their optimized N/P ratio of 28-30. mRPZ significantly outperforms its optimized isoforms, except with branched 25 kDa PEI, which has poor transfection efficiency alone and in mRPZ. Data presented as mean +/- StdEM, the sample size of each sample is N=16, and statistical significance (P) was assessed using one-way ANOVA.

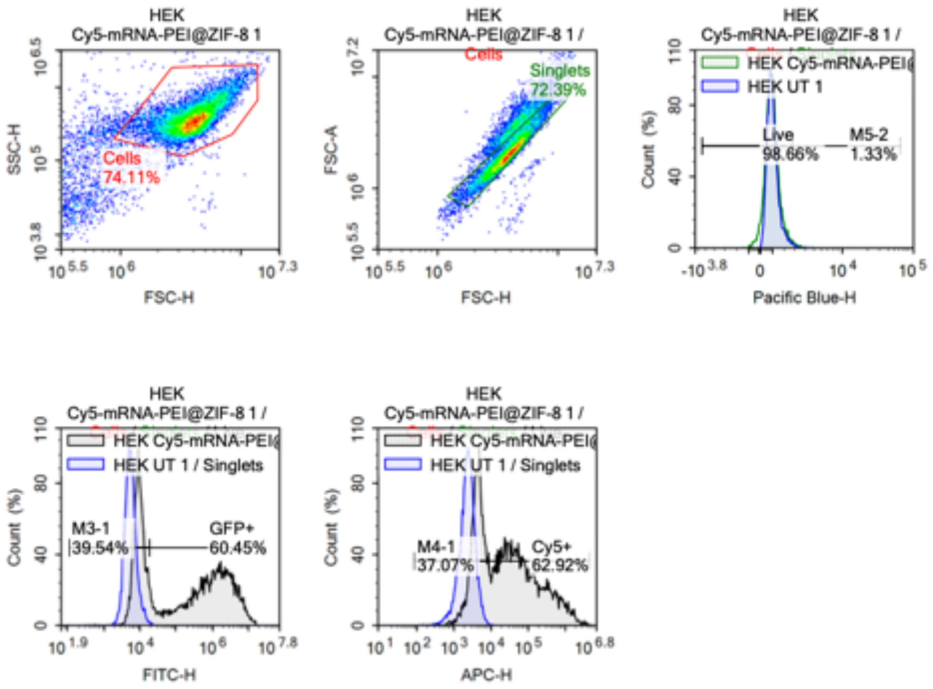

Sample Statistics of HEK Cy5-mRNA-PEI@ZIF-8 1

Gate	Count	% Parent	X	Y	Median X	Median Y
All	18,496					
Cells	13,707	74.11 %	FSC-H	SSC-H	3,939,024	360,393
Singlets	9,922	72.39 %	FSC-H	FSC-A	3,756,250	2,029,058
Live	9,789	98.66 %	Pacific Blue-H	Count	957	
M3-1	3,871	39.54 %	FITC-H	Count	9,569	
GFP+	5,917	60.45 %	FITC-H	Count	1,078,596	
M4-1	3,629	37.07 %	APC-H	Count	4,482	
Cy5+	6,159	62.92 %	APC-H	Count	60,451	
M5-2	132	1.33 %	Pacific Blue-H	Count	3,242	

Figure S24. Flow cytometry report printout for HEK293t cells transfected with Cy5-labeled eGFP-encoding mRPZ nanoparticles. The same gating strategy was used across cell lines. Cells were gated by first identifying the cell population among the debris, then singlet cells were selected. Of those remaining, singlet cells were gated by comparing viable cells to an untreated group. The live, single-cell populations were then measured for GFP and Cy5 intensity.

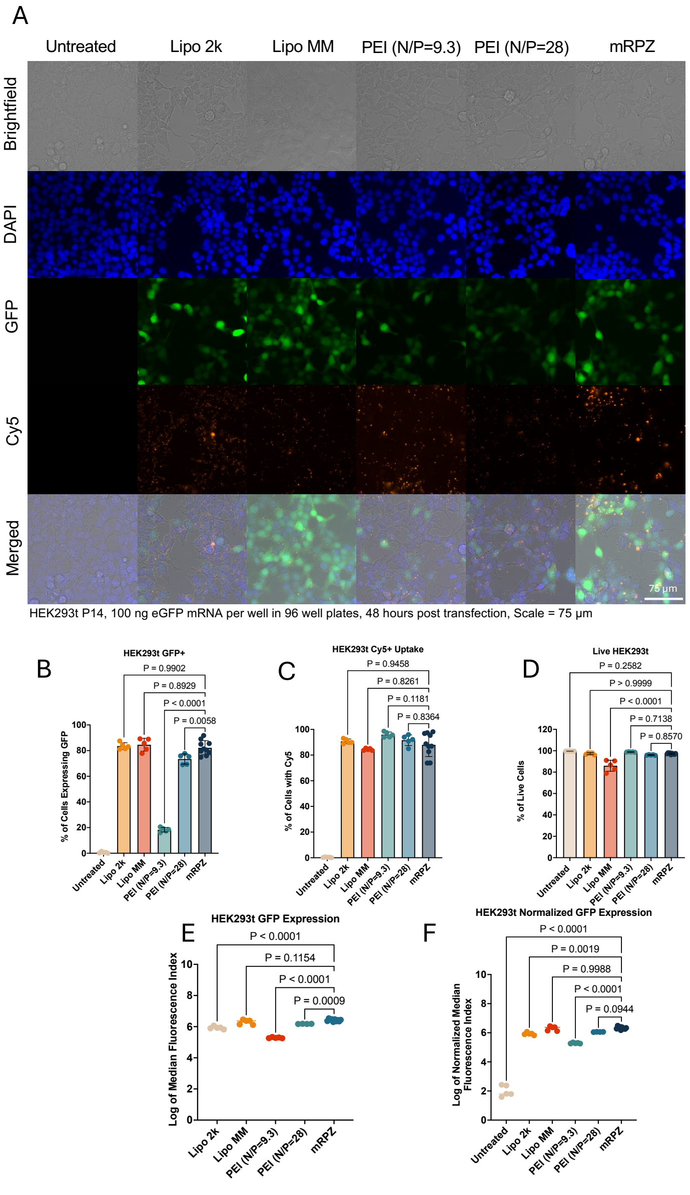

HEK293t P17, 100 ng eGFP mRNA per well in 96 well plates, 48 hours post transfection

Figure S25. HEK293t cells were treated with Cy5-Tagged, eGFP-expressing mRNA carried by mRPZ and various controls, at 48 hours post-transfection the cells were fixed, stained, imaged, and analyzed with flow cytometry. Microscopy images **(A)** confirm successful uptake (Cy5) and expression (GFP) of the mRNA in cells stained with a nuclear stain (DAPI), scale bar = 75 µm. The resultant expression **(B)**, uptake **(C)**, and cell viability **(D)** were quantified with flow cytometry. The median fluorescent index (MFI) **(E)** and MFI normalized to cells that were GFP+ **(F)** were also quantified. Data represented as mean +/- standard deviation, sample sizes were N = 5, except mRPZ N=10, and statistical significance (P) was assessed with one-way ANOVA. Biological replicates were performed.

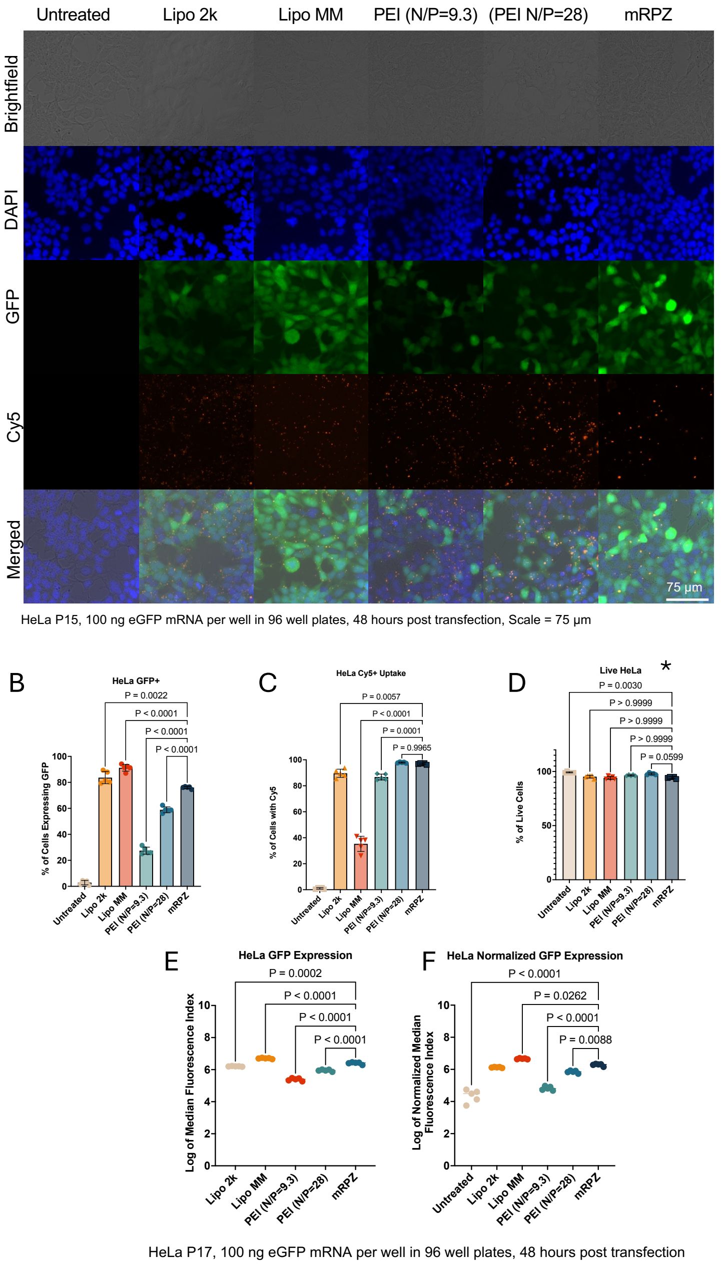

Figure S26. HeLa cells were treated with Cy5-Tagged, eGFP-expressing mRNA carried by mRPZ and various controls, at 48 hours post-transfection the cells were fixed, stained, imaged, and analyzed with flow cytometry. Microscopy images **(A)** confirm successful uptake (Cy5) and expression (GFP) of the mRNA in cells stained with a nuclear stain (DAPI), scale bar = 75 µm. The resultant expression **(B)**, uptake **(C)**, and cell viability **(D)** were quantified with flow cytometry. The median fluorescent index (MFI) **(E)** and MFI normalized to cells that were GFP+ **(F)** were also quantified. Data presented as mean +/- standard deviation, sample sizes were N = 5, and statistical significance (P) was assessed with one-way ANOVA. Asterisks (*) denote statistics that were run with parametric comparisons due to non-normally distributed data. Biological replicates were performed.

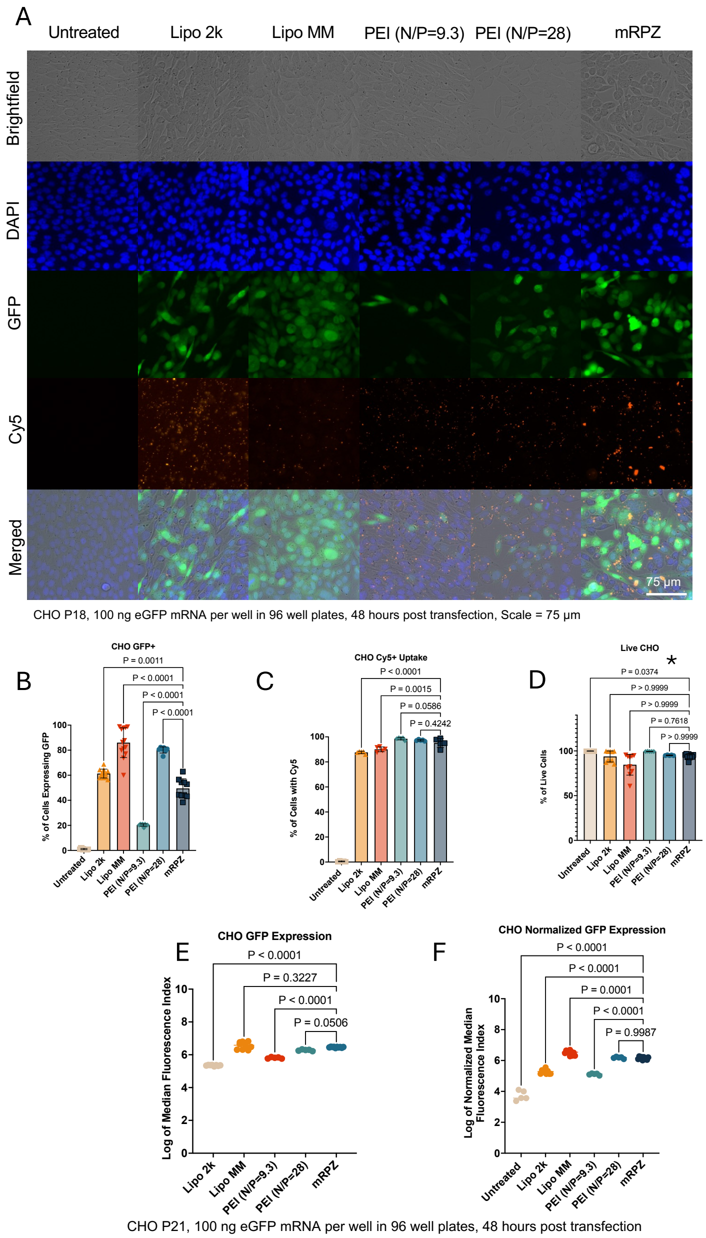

Figure S27. CHO cells were treated with Cy5-Tagged, eGFP-expressing mRNA carried by mRPZ and various controls, at 48 hours post-transfection the cells were fixed, stained, imaged, and analyzed with flow cytometry. Microscopy images **(A)** confirm successful uptake (Cy5) and expression (GFP) of the mRNA in cells stained with a nuclear stain (DAPI), scale bar = 75 µm. The resultant expression **(B)**, uptake **(C)**, and cell viability **(D)** were quantified with flow cytometry. The median fluorescent index (MFI) **(E)** and MFI normalized to cells that were GFP+ **(F)** were also quantified. Data presented as mean +/- standard deviation, sample sizes were N = 10 (except for PEI samples N =5), and statistical significance (P) was assessed with one-way ANOVA. Asterisks (*) denote statistics that were run with parametric comparisons due to non-normally distributed data. Biological replicates were performed.

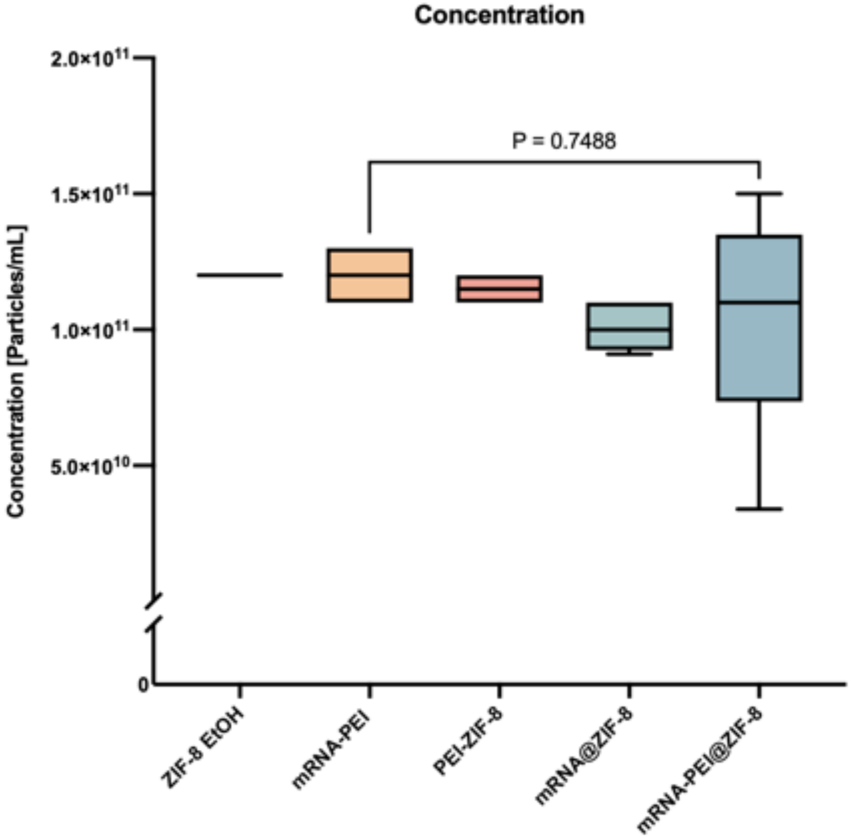

	mRNA-PEI	mRNA-PEI@ZIF-8	mRNA@ZIF-8	PEI-ZIF-8	ZIF-8 EtOH
Number of values	6	10	5	4	2
Minimum	1.10E+11	3.40E+10	9.10E+10	1.10E+11	1.20E+11
Maximum	1.30E+11	1.50E+11	1.10E+11	1.20E+11	1.20E+11
Range	2.00E+10	1.16E+11	1.90E+10	1.00E+10	0.00E+00
Mean	1.20E+11	1.03E+11	1.01E+11	1.15E+11	1.20E+11
Std. Deviation	8.94E+09	4.11E+10	8.83E+09	5.77E+09	0.00E+00
Std. Error of Mean	3.65E+09	1.30E+10	3.95E+09	2.89E+09	0.00E+00

Figure S28. Nanoparticle tracking shows the concentration of particles produced using 2 µg of mRNA (**Top**). Considering a nominal loading efficiency of 90%, the number of mRNAs per particle was calculated for each type of RNA-containing particle **(Bottom)**. The number of values represents the number of tests run. Data presented as min, median, and max, sample sizes are "number of values" stated in the table, and statistical significance (P) was assessed using one-way ANOVA.

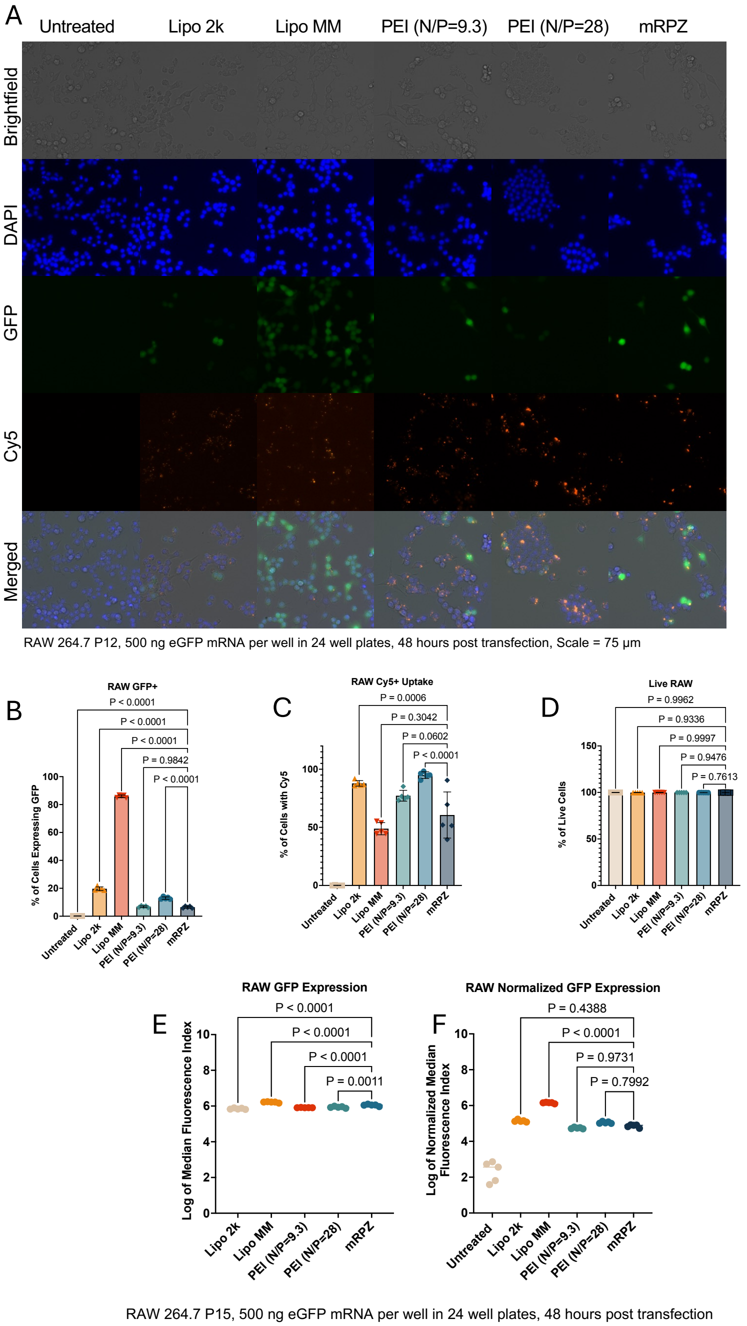

Figure S29. RAW264.7 cells were treated with Cy5-Tagged, eGFP-expressing mRNA carried by mRPZ and various controls, at 48 hours post-transfection the cells were fixed, stained, imaged, and analyzed with flow cytometry. Microscopy images **(A)** confirm successful uptake (Cy5) and expression (GFP) of the mRNA in cells stained with a nuclear stain (DAPI), scale bar = 75 μm. The resultant expression **(B)**, uptake **(C)**, and cell viability **(D)** were quantified with flow cytometry. The median fluorescent index (MFI) **(E)** and MFI normalized to cells that were GFP+ **(F)** were also quantified. Data presented as mean +/- standard deviation, sample sizes were N =5, and statistical significance (P) was assessed with one-way ANOVA.

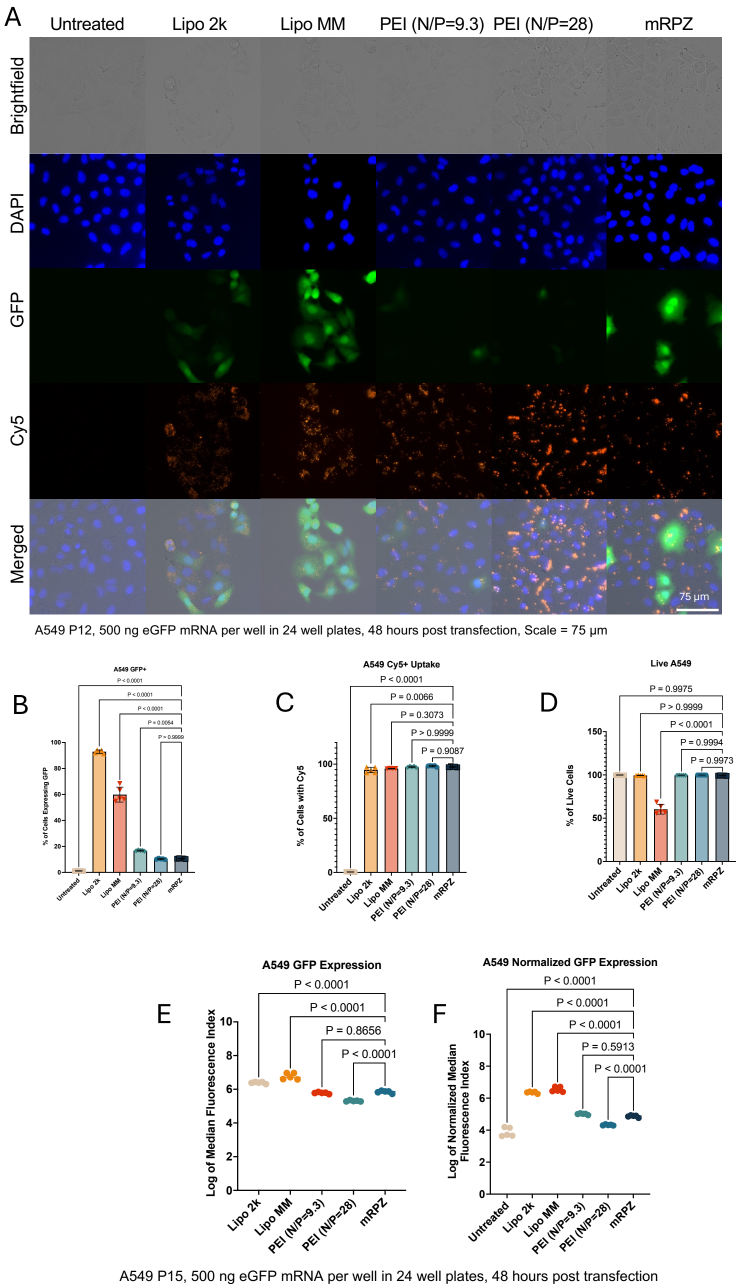

Figure S30. A549 cells were treated with Cy5-Tagged, eGFP-expressing mRNA carried by mRPZ and various controls, at 48 hours post-transfection the cells were fixed, stained, imaged, and analyzed with flow cytometry. Microscopy images **(A)** confirm successful uptake (Cy5) and expression (GFP) of the mRNA in cells stained with a nuclear stain (DAPI), scale bar = 75 µm. The resultant expression **(B)**, uptake **(C)**, and cell viability **(D)** were quantified with flow cytometry. The median fluorescent index (MFI) **(E)** and MFI normalized to cells that were GFP+ **(F)** were also quantified. Data presented as mean +/- standard deviation, sample sizes were N =5, and statistical significance (P) was assessed with one-way ANOVA.

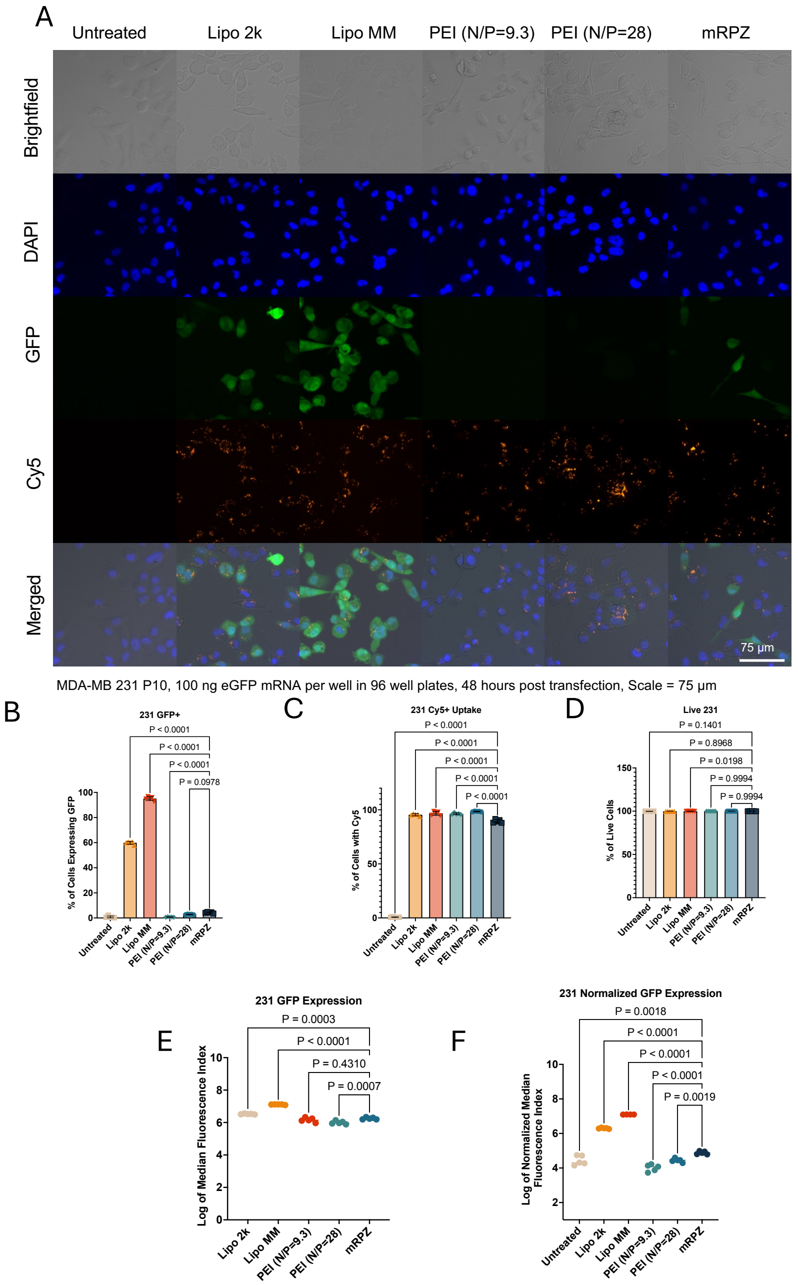

MDA-MB 231 P15, 100 ng eGFP mRNA per well in 96 well plates, 48 hours post transfection

Figure S31. MDA-MB-231 cells were treated with Cy5-Tagged, eGFP-expressing mRNA carried by mRPZ and various controls, at 48 hours post-transfection the cells were fixed, stained, imaged, and analyzed with flow cytometry. Microscopy images **(A)** confirm successful uptake (Cy5) and expression (GFP) of the mRNA in cells stained with a nuclear stain (DAPI), scale bar = 75 µm. The resultant expression **(B)**, uptake **(C)**, and cell viability **(D)** were quantified with flow cytometry. The median fluorescent index (MFI) **(E)** and MFI normalized to cells that were GFP+ **(F)** were also quantified. Data presented as mean +/- standard deviation, sample sizes were N =5, and statistical significance (P) was assessed with one-way ANOVA.

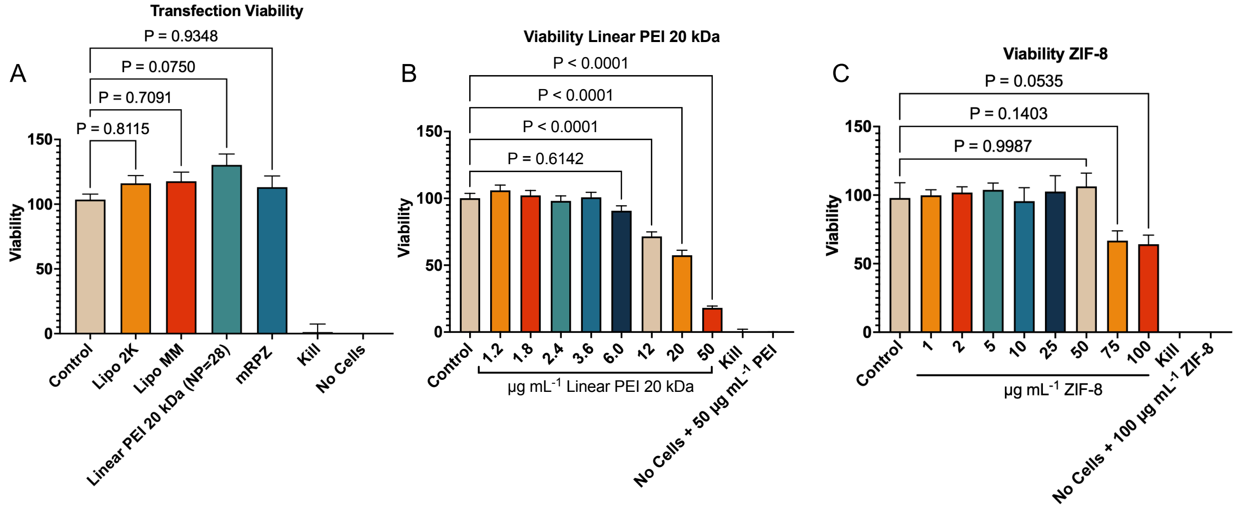

Figure S32. HEK293t P22 cells were treated with mRPZ, components, and transfection reagents. After a 4–6-hour exposure, the metabolic activity of the cells was measured by resazurin conversion for 8 hours. The fluorescence produced from the reacted resazurin was measured using a plate reader, normalized, and compared to transfection reagents **(A)**, Linear 20 kDa PEI alone **(B)**, and ZIF-8 alone **(C)**. Biological replicates were performed. Data presented as mean +/- StdEM, sample sizes are N≥5, and statistical significance (P) assessed with one-way ANOVA.

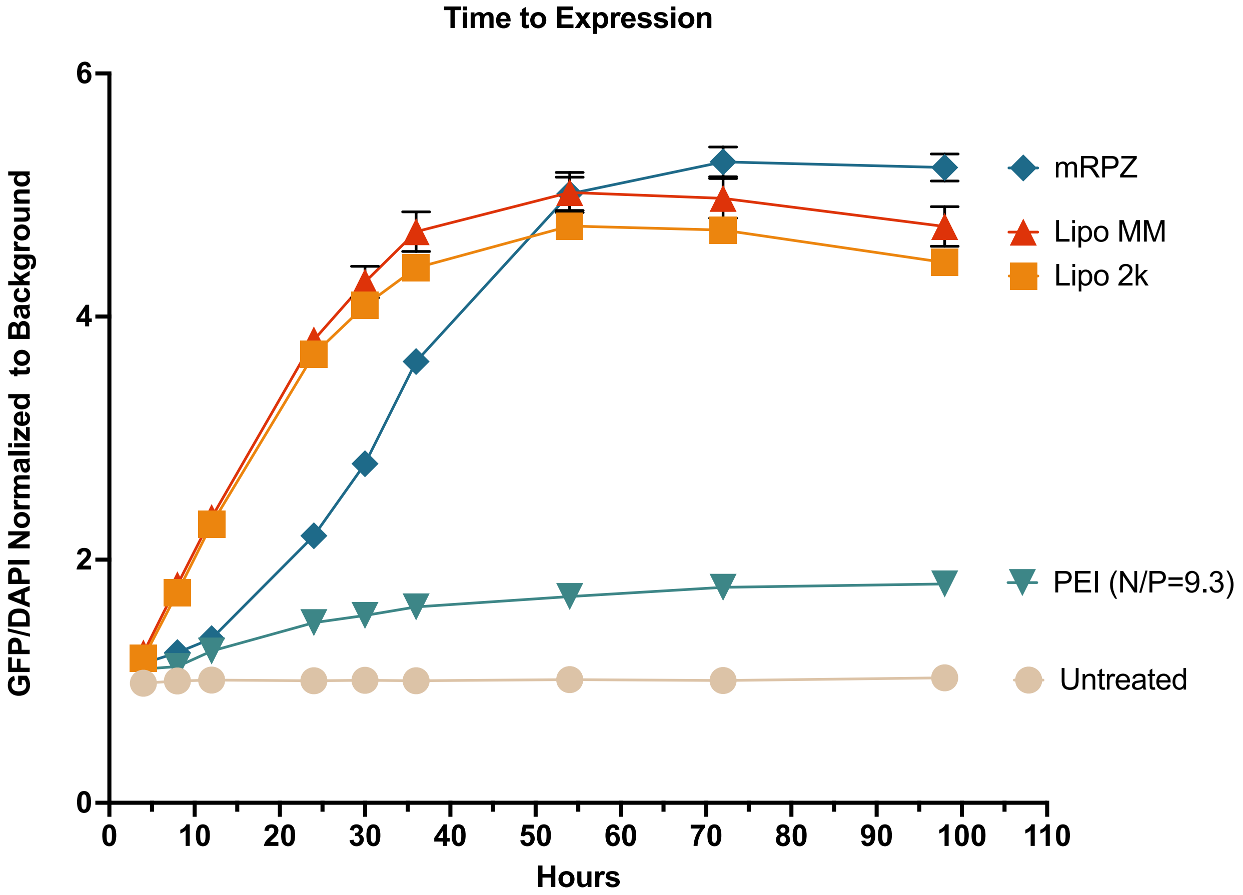

Figure S33. eGFP expression over 96 hours of HEK293t cells after transfection of eGFP mRNA with mRPZ (mRNA-PEI@ZIF-8), RNA-PEI, and Lipo2k and Lipo MM as positive controls. Data presented as mean +/- StdEM. Biological replicate performed and included in data.

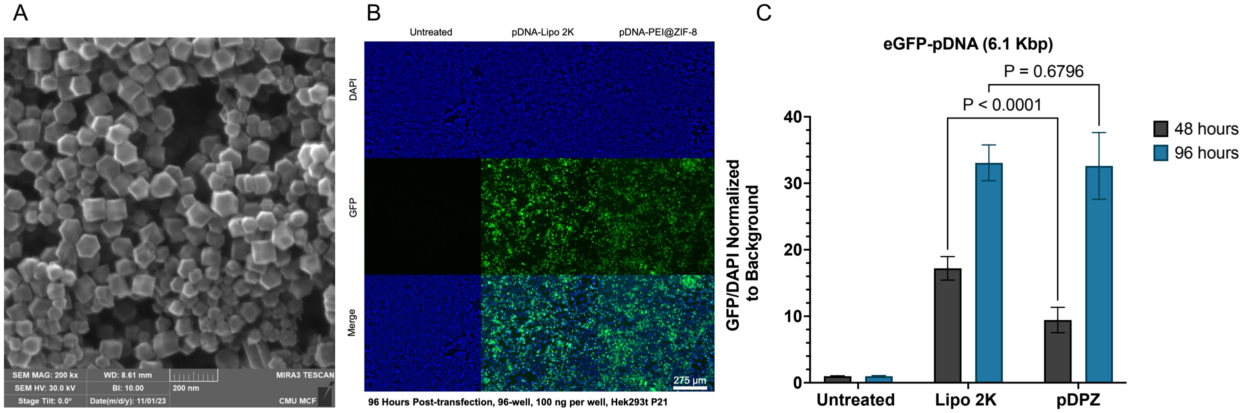

Figure S34. pDNA-PEI@ZIF-8 was prepared and imaged using **(A)** SEM (scale bar = 200 nm), then applied **(B)** transfect HEK293t cells *in vitro* with (scale bar = 275 μm) **(C)** fluorescence quantified with a plate reader. Data presented as mean +/- standard deviation, and the sample sizes are as follows: untreated and mRPZ N=16 and Lipo 2k N=8, the statistical significance (P) was assessed using a two-way ANOVA.

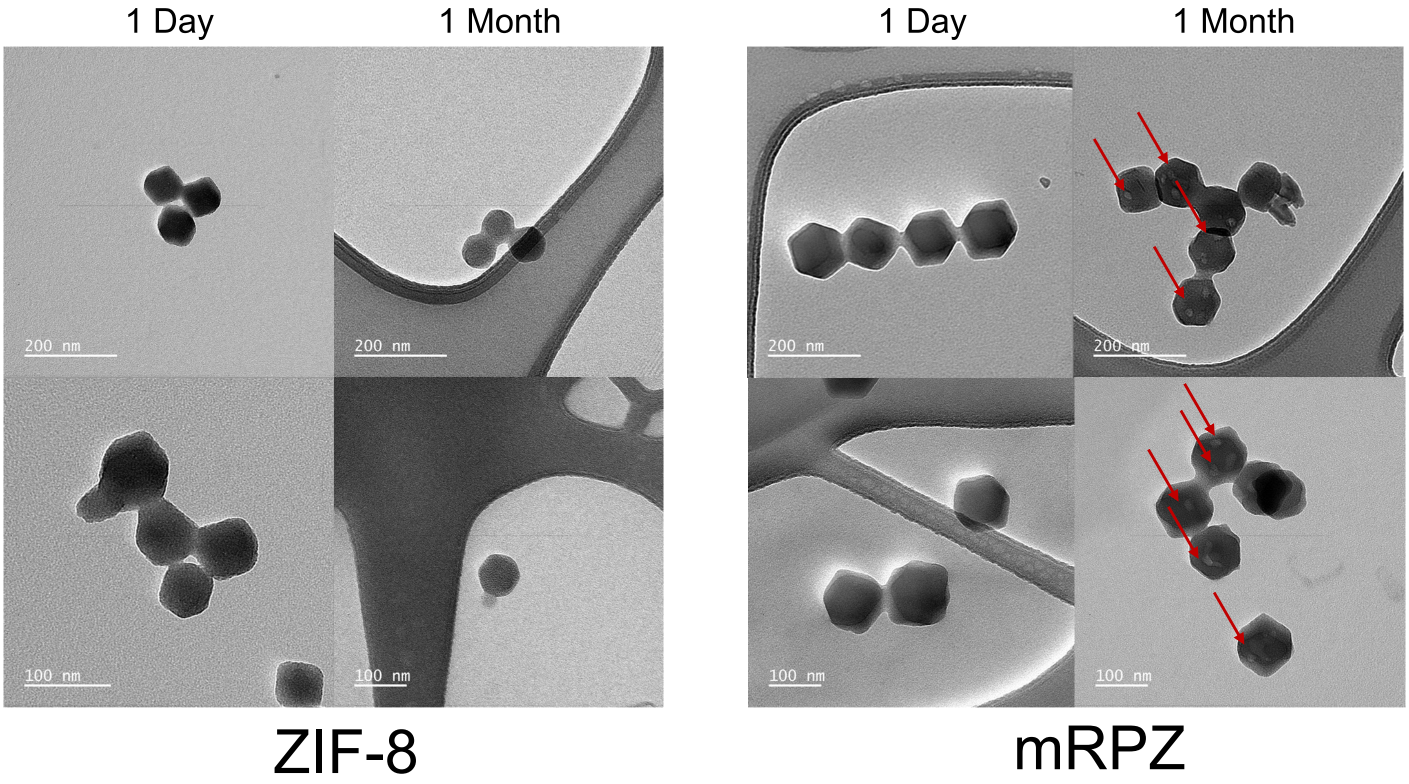

Figure S35. ZIF-8 and mRPZ were imaged under TEM 1 day and 1 month after being synthesized and stored at room temperature. ZIF-8 demonstrated a clear dodecahedron shape on day 1 and gradually became more spherical at 1 month. mRPZ maintains its dodecahedron shape but develops cavities (red arrows) within itself. The top row scale bar = 200 nm and the bottom row scale bar = 100 nm.

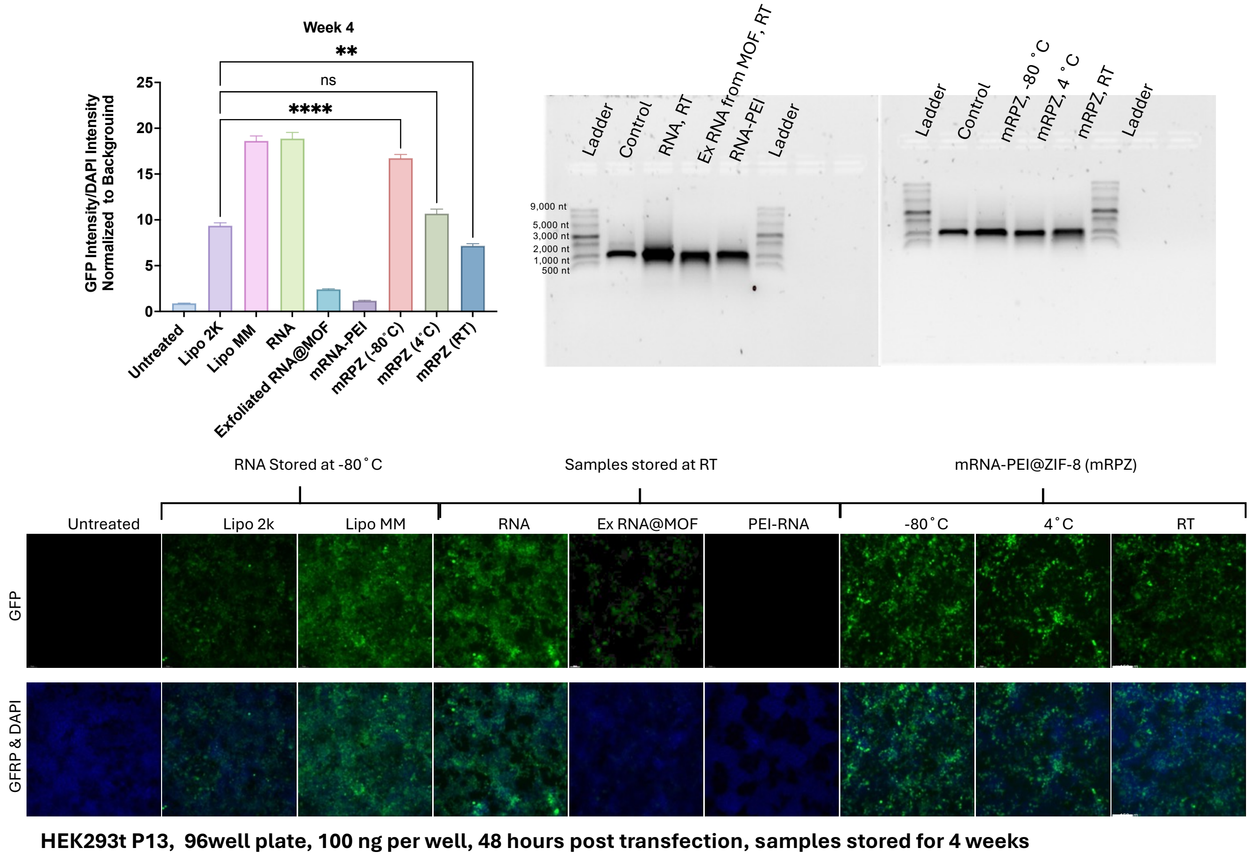

Figure S36. After storage at various temperatures for 4 weeks, mRPZ was applied *in vitro* along with controls. mRPZ (mRNA-PEI@ZIF-8) samples were stored at -80˚C, 4˚C, and room temperature (RT). Lipo 2k and Lipo MM RNA were stored at -80˚C. Dry mRNA (RNA), exfoliated mRNA from mRNA@MOF (Ex RNA@MOF), and PEI-mRNA (PEI-RNA) samples were stored at RT. The expression was quantified using a plate reader **(Top Left)**. The length of the mRNA was assessed using a native RNA gel **(Top Right)**. Fluorescence images after cell transfection were obtained for the green channel (eGFP) and blue channel (DAPI) **(Bottom)**. Statistical significance (P) assessed by one-way ANOVA (N=32) is described as follows: ns – not statistically significant, * for P <0.05, ** for P <0.01, *** for P <0.005, and **** for P <0.001. Scale Bar 150 μm. Performed using biological triplicates with technical replicates.

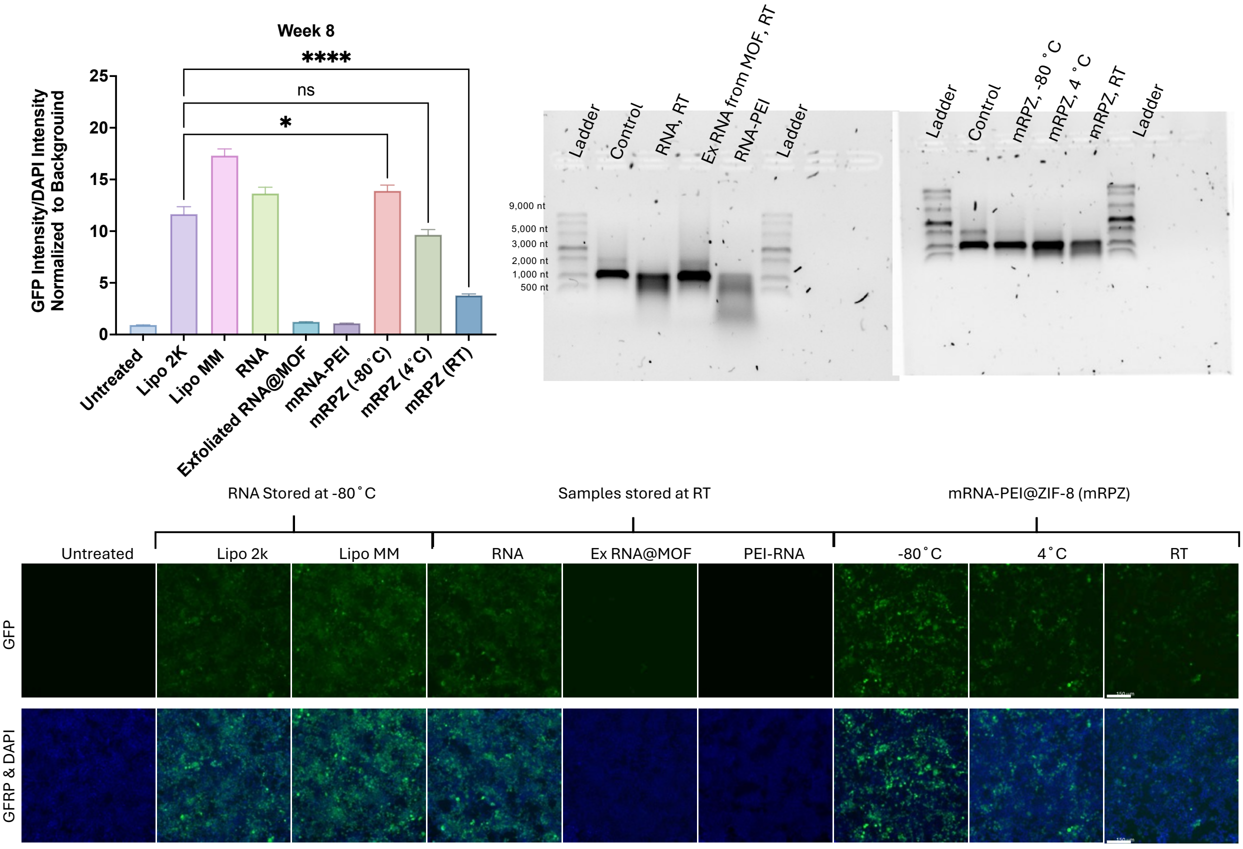

HEK293t P21, 96well plate, 100 ng per well, 48 hours post transfection, samples stored for 8 weeks

Figure S37. After storage at various temperatures for 8 weeks, mRPZ was applied *in vitro* along with controls. mRPZ (mRNA-PEI@ZIF-8) samples were stored at -80˚C, 4˚C, and room temperature (RT). Lipo 2k and Lipo MM RNA were stored at -80˚C. Dry mRNA (RNA), exfoliated mRNA from mRNA@MOF (Ex RNA@MOF), and PEI-mRNA (PEI-RNA) samples were stored at RT. The expression was quantified using a plate reader **(Top Left)**. The length of the mRNA was assessed using a native RNA gel **(Top Right)**. Fluorescence images after cell transfection were obtained for the green channel (eGFP) and blue channel (DAPI) **(Bottom)**. Statistical significance (P) assessed by one-way ANOVA (N=45) is described as follows: ns – not statistically significant, * for P <0.05, ** for P <0.01, *** for P <0.005, and **** for P <0.001. Scale Bar 150 µm. Performed using biological triplicates with technical replicates.

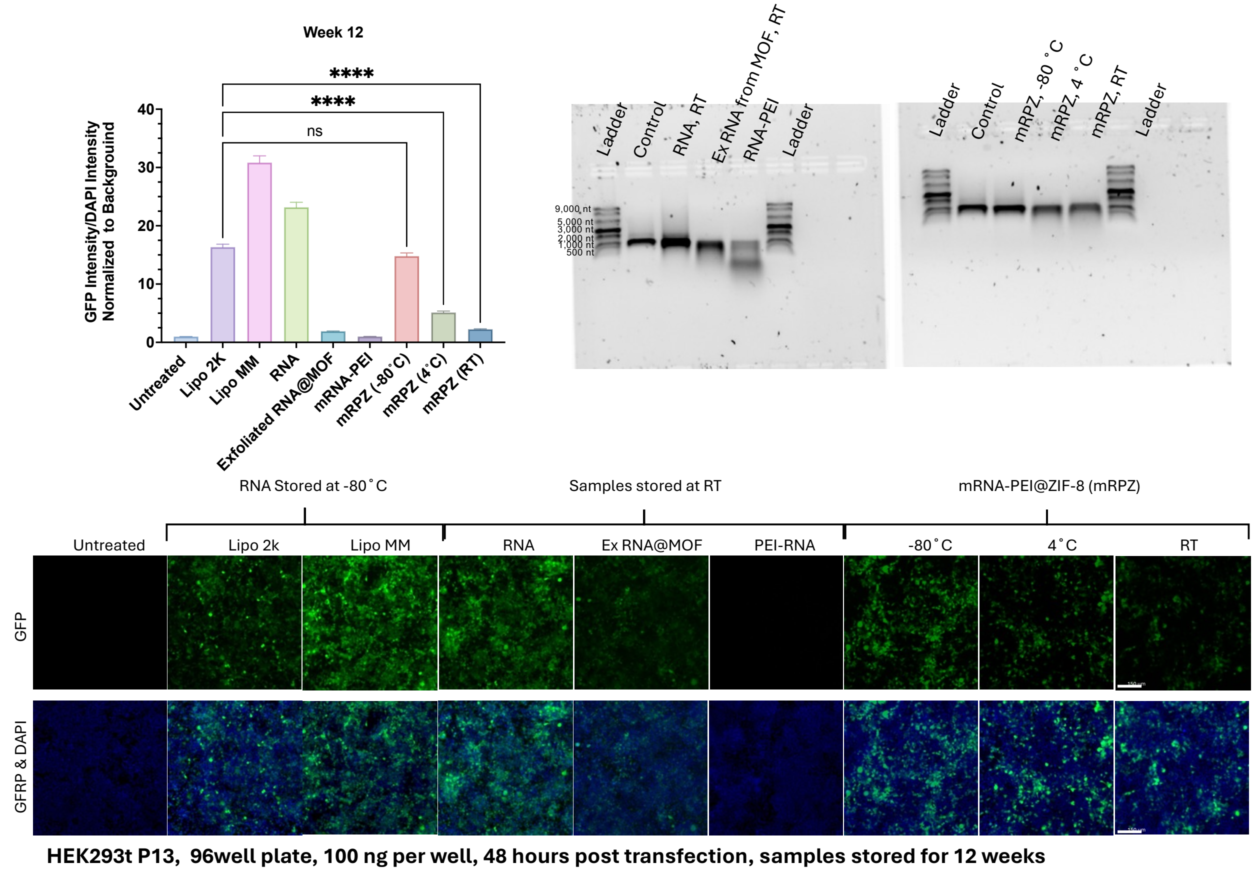

Figure S38. After storage at various temperatures for 12 weeks, mRPZ was applied *in vitro* along with controls. mRPZ (mRNA-PEI@ZIF-8) samples were stored at -80˚C, 4˚C, and room temperature (RT). Lipo 2k and Lipo MM RNA were stored at -80˚C. Dry mRNA (RNA), exfoliated mRNA from mRNA@MOF (Ex RNA@MOF), and PEI-mRNA (PEI-RNA) samples were stored at RT. The expression was quantified using a plate reader **(Top Left)**. The length of the mRNA was assessed using a native RNA gel **(Top Right)**. Fluorescence images after cell transfection were obtained for the green channel (eGFP) and blue channel (DAPI) **(Bottom)**. Statistical significance (P) assessed by one-way ANOVA (N=45) is described as follows: ns – not statistically significant, * for P <0.05, ** for P <0.01, *** for P <0.005, and **** for P <0.001. Scale Bar 150 µm. Performed using biological triplicates with technical replicates.

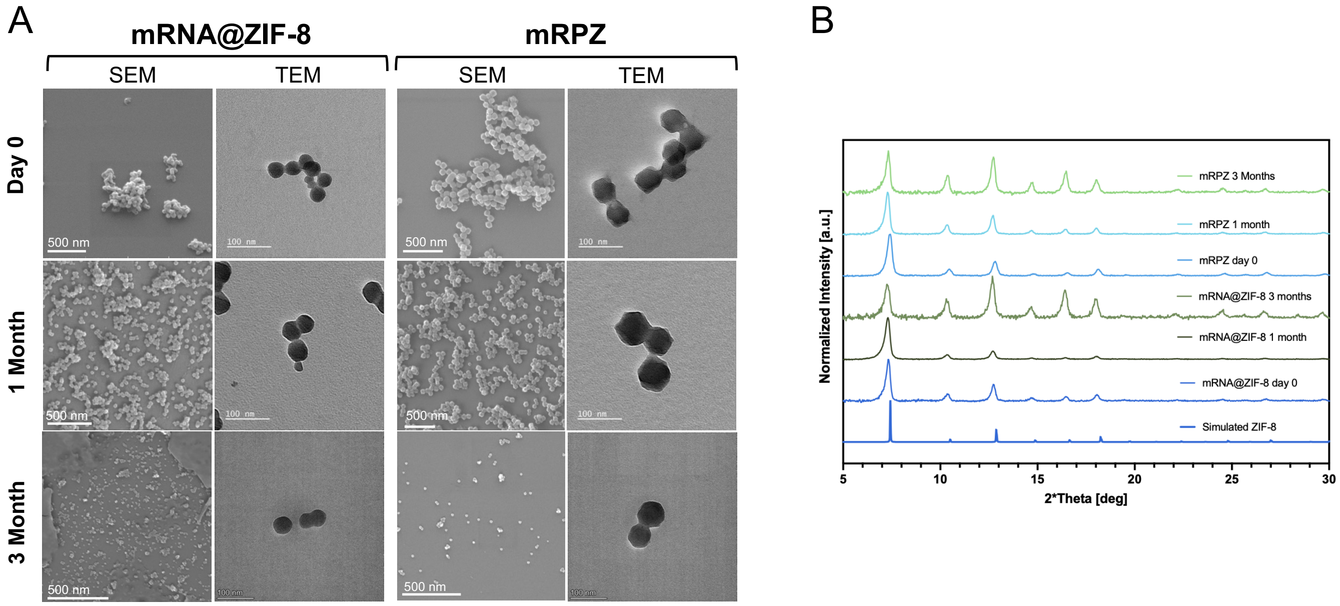

Figure S39. mRNA@ZIF-8 and mRPZ material size, morphology **(A)**, and crystallinity **(B)** were tracked over 3 months using SEM (Scale bars = 500 nm), TEM (Scale bars = 100 nm), and PXRD.

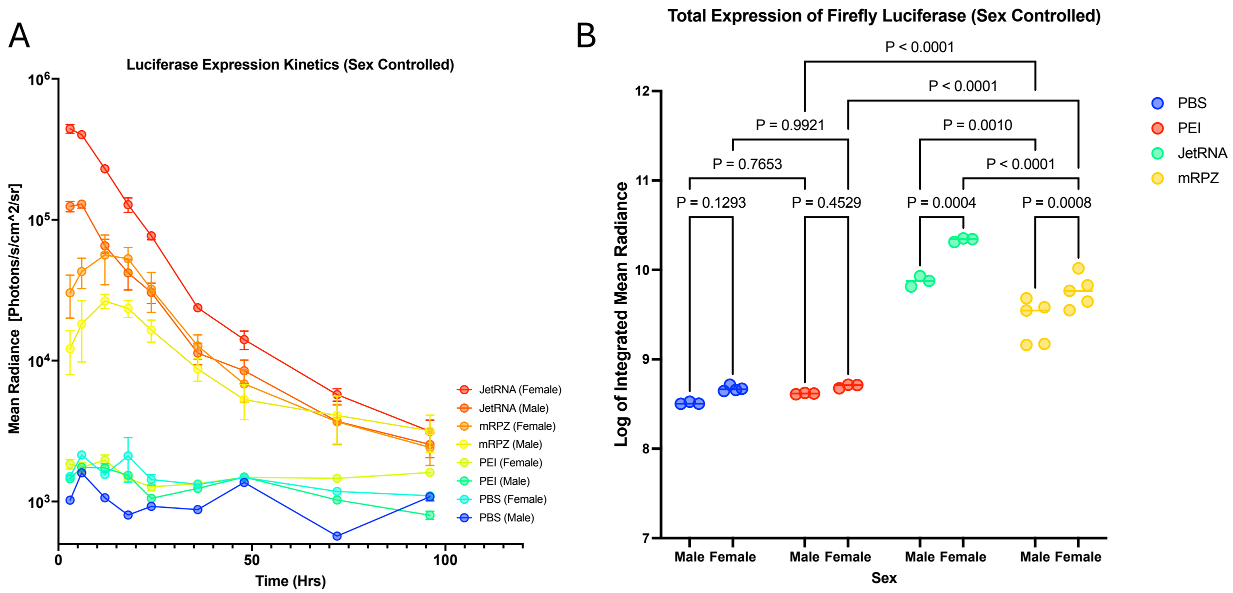

Figure S40. Firefly luciferase expressing mRNA was encapsulated using our novel encapsulation procedure inside a composite PEI/ZIF-8 particle and delivered to Balb/C mice intravenously. Luciferase expression was measured by luminescence resulting from intraperitoneal luciferin injection. The luciferase activity kinetics were measured over 96 hours and the data shown represent mean +/- StdEM **(A).** The integrated luciferase activity, which correlates to total luciferase expression was calculated and the data shown represent the median **(B)** The sample size of each group is as follows: all are N=3-4 except mRPZ were both male and female were N=5. The statistical significance (P) was assessed using a two-way ANOVA.

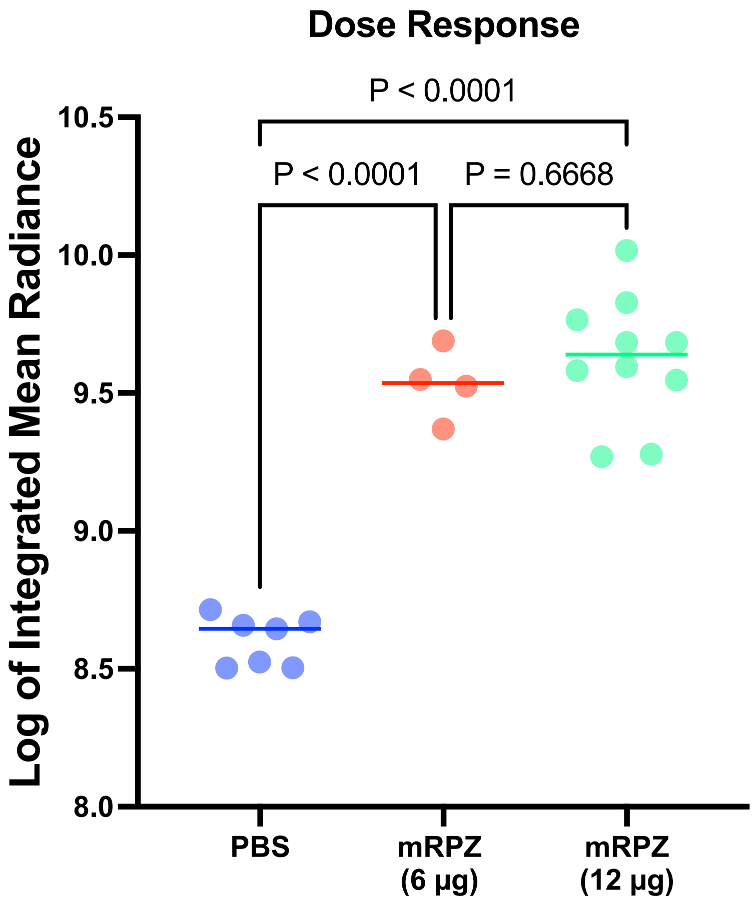

Figure S41. Varying dosages of mRNA were delivered *in vivo* using mRPZ. Data presented as median, sample sizes are as follows PBS (N=6), mRPZ 6 µg (N=4), and mRPZ 12 µg (N=10), statistical significance (P) was assessed using a one-way ANOVA.

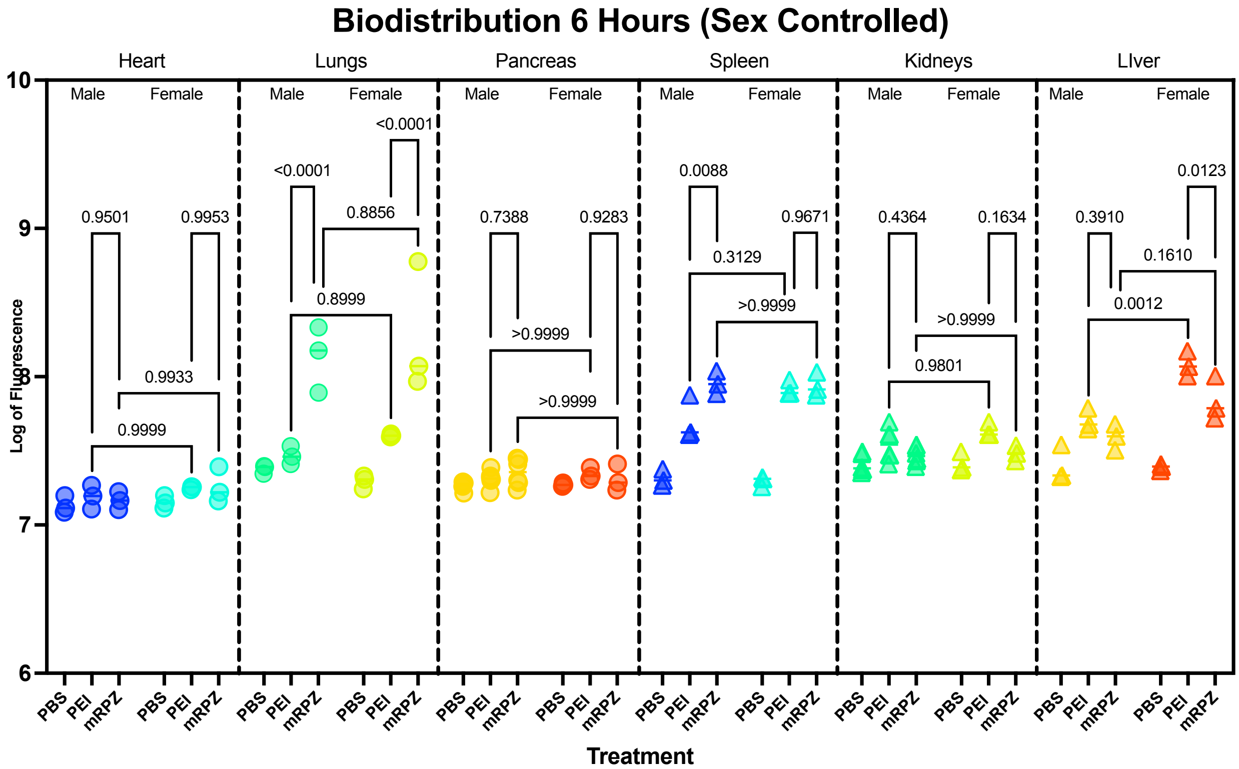

Figure S42. Cy5 tagged mRNA expressing luciferase was delivered to Balb/C mice, and after 6 hours the mice organs were collected and imaged. The biodistribution of the mRNA, and hence particles, was quantified, shown here separated by sex. Data is represented as the median, sample size is N=3 for all samples, and statistical significance (P) was assessed with two-way ANOVA.

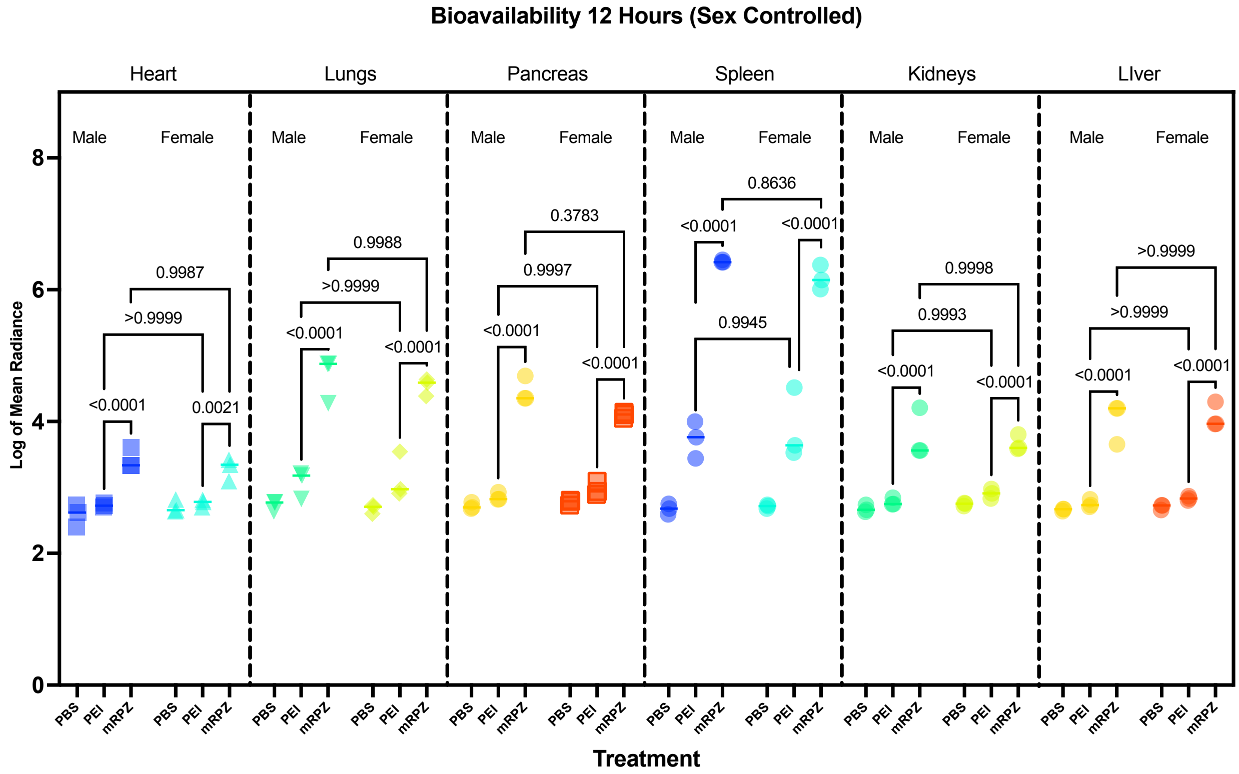

Figure S43. The bioavailability of the luciferase mRNA was quantified after 12 hours, shown here separated by sex. Data is represented as the median, sample size is N=3 for all samples, and statistical significance (P) was assessed with two-way ANOVA.

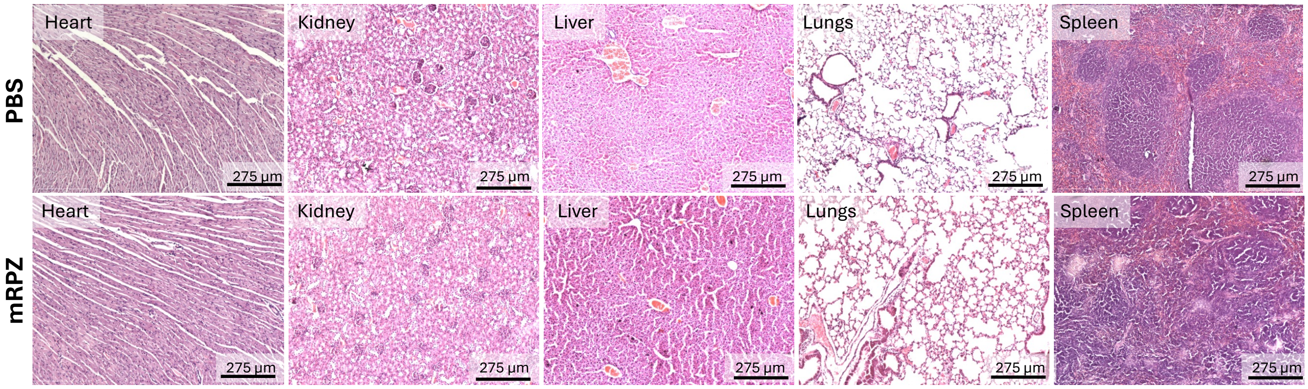

Figure S44. Hematoxylin and eosin (H&E) staining of major organs (heart, kidney, liver, lung, and spleen) collected one week post-administration of mRPZ. No signs of inflammation, necrosis, or structural abnormalities were observed in treated tissues compared to PBS controls. Tissue architecture remained intact, with well-defined nuclei (hematoxylin, blue) and cytoplasmic components (eosin, pink), indicating minimal acute toxicity. Scale bars = 275 μm.

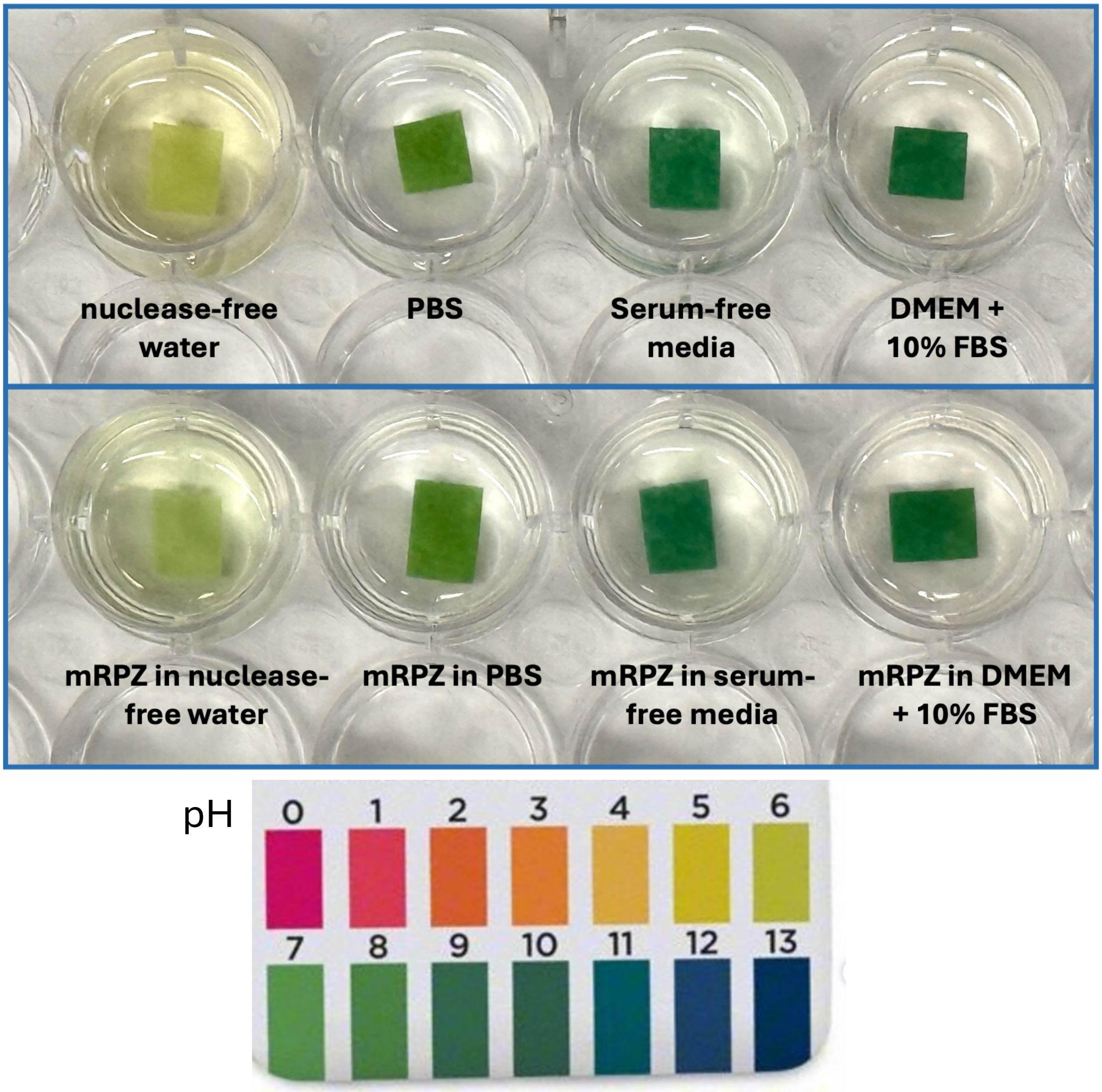

Figure S45. pH stability of mRPZ-formulated mRNA in various biologically relevant buffers. Samples containing 12 µg of mRNA encapsulated in mRPZ nanoparticles were incubated at 37 °C in 100 µL of nuclease-free water, 1× PBS, serum-free media, or media supplemented with 10% fetal bovine serum (FBS). After incubation, the pH of each solution was measured using pH indicator paper to assess buffering effects and potential pH shifts induced by nanoparticle components.
